# HPLC, quantitative NMR and HRMS spectroscopic data of nusbiarylins as a new class of antimicrobial agents

**DOI:** 10.1016/j.dib.2020.105313

**Published:** 2020-02-21

**Authors:** Yangyi Qiu, Cong Ma

**Affiliations:** State Key Laboratory of Chemical Biology and Drug Discovery, Department of Applied Biology and Chemical Technology, The Hong Kong Polytechnic University, Kowloon, Hong Kong Special Administrative Region

**Keywords:** Inhibitor, Bacterial transcription, NusB-NusE interaction, HPLC, qNMR, HRMS

## Abstract

Bacterial transcription is a valid but underutilized target for antimicrobial agent discovery [1]. Nusbiarylins are the first-in-class bacterial ribosomal RNA synthesis inhibitors that possess potent activity against various types of multidrug-resistant bacteria with a novel mode of action by targeting the interaction of bacterial transcription factors NusB and NusE [2]. To facilitate the characterization of nusbiarylin derivatives produced by other researchers, high-performance liquid chromatography (HPLC) profiles, quantitative nuclear magnetic resonance (qNMR) and high-resolution mass spectrometry (HRMS) spectroscopic data were presented for the quick determination of purity and characterization of 95 nusbiarylin compounds. The data presented in this article supplement the ^1^H and ^13^C NMR data provided previously [3,4], and assist the reproduction of nusbiarylins for chemical, biological and drug discovery research.

**Table:** Specifications Table

Subject	Chemistry
Specific subject area	Organic chemistry Analytical chemistry
Type of data	Table Figure
How data were acquired	Agilent 1100 series and 1260 infinity system Bruker ultrashield™ NMR spectrometer 600 MHz Agilent Technologies 6520 Accurate-Mass Q-TOF LC/MS spectrometer
Data format	Raw (as supplementary file) Analyzed
Parameters for data collection	The purified compounds were subjected to HPLC and qNMR analysis. The mobile phase for HPLC analysis were acetonitrile and water. The ratio was specified in the “Experimental Design, Materials, and Methods” section. The flow rate was set as 1.000 mL/min. Compounds were dissolved in *d*-DMSO prior to qNMR analysis. The parameters for qNMR analysis were adjusted according to the literature [5].
Description of data collection	HPLC profiles of 95 novel compounds were recorded on and exported from an Agilent 1100 series and 1260 infinity system. Area% and RetTime stands for purity and retention time, respectively. qNMR spectra data of 95 novel compounds were recorded on and exported form a Bruker ultrashield™ NMR spectroscope 600 MHz spectrometer using standard Bruker pulse programs. Chemical shifts were shown as δ-values. Positive- and negative-ion HRESI-TOF-MS of 95 novel compounds were recorded on and exported from an Agilent Technologies 6520 Accurate-Mass Q-TOF LC/MS spectrometer.
Data source location	Department of Applied Biology and Chemical Technology, the Hong Kong Polytechnic University, Hong Kong SAR
Data accessibility	Data are available with the article

**Table:** 

**Value of the Data** • Nusbiarylins are the first-in-class bacterial ribosomal RNA synthesis inhibitors that possess potent activity against various types of multidrug-resistant bacteria with a novel mode of action • Spectral data of nusbiarylins are useful for elucidating their purity • The HPLC profiles, qNMR and HRMS spectroscopic data are of 95 unreported compounds and could be useful for the characterization by other researchers

## 1 Data

Bacterial transcription is a valid but underutilized target for antimicrobial agent discovery [1]. NusB and NusE are bacteria-specific transcription factors essential for cell viability [1,2]. Inhibitors of the NusB-NusE interaction were discovered and named nusbiarylins. The name was derived from the target protein NusB and their biaryl structure [[2], [3], [4]]. The dataset contains high-performance liquid chromatography (HPLC) profiles of 90 compounds, quantitative nuclear magnetic resonance (qNMR) spectroscopic data of 5 compounds and high-resolution mass spectrometry (HRMS) profiles of all 95 compounds [3,4]. The data file (HPLC, qNMR and HRMS spectra) is available publicly within this data article as a supplementary file. The compound structures were presented in Table 1, purities in Table 2 and HRMS data in Table 3. The testing methods and parameters of different compounds by HPLC, HRMS and qNMR were also described.

**Table 1:**
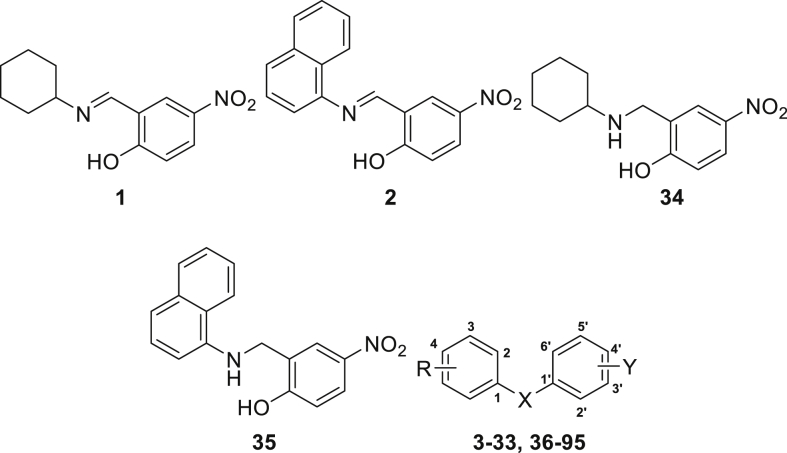

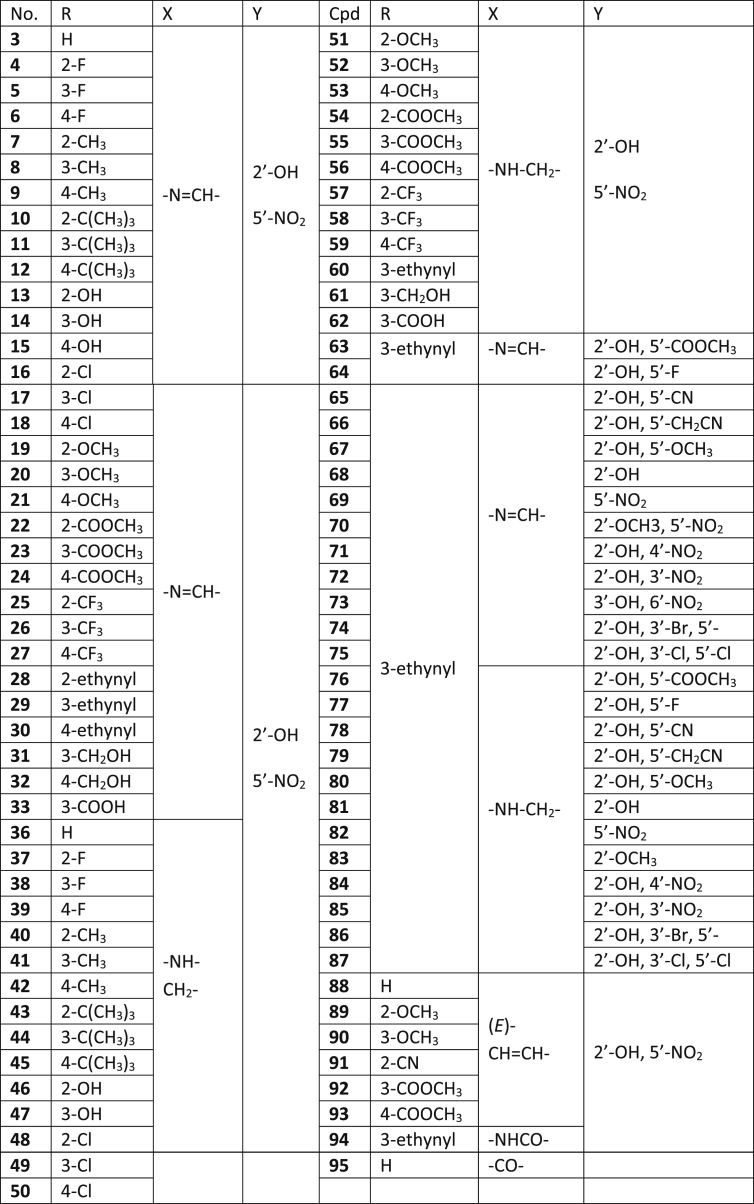
Chemical structures of 95 nusbiarylin compounds as NusB-NusE inhibitors.

**Table 2:** Data on purities by HPLC or qNMR and retention time (HPLC) of nusbiarylin compounds.

Compound	Purity/%	Retention time/min
**1**	99.9	6.51
**2**	99.0	11.23
**3**	99.8	9.06
**4**	98.2	9.35
**5**	98.0	9.47
**6**	100.0	9.32
**7**	99.5	9.90
**8**	99.8	10.12
**9**	99.9	10.12
**10**	99.4	12.10
**11**	100.0	12.35
**12**	99.2	12.50
**13**	96.0	5.66
**14**	97.8	6.59
**15**	96.1	6.35
**16**	98.5	10.17
**17**	96.6	10.51
**18**	95.5	10.50
**19**	97.8	7.80
**20**	98.7	9.04
**21**	99.9	9.02
**22**	98.4	qNMR
**23**	97.2	9.15
**24**	98.9	qNMR
**25**	100.0	10.09
**26**	98.3	10.55
**27**	97.3	qNMR
**28**	97.4	8.98
**29**	97.0	9.76
**30**	95.5	9.69
**31**	95.3	5.95
**32**	97.2	5.77
**33**	91.0	qNMR
**34**	99.2	9.21
**35**	100.0	7.86
**36**	99.4	6.48
**37**	99.7	6.81
**38**	97.7	6.70
**39**	99.8	6.58
**40**	99.8	7.14
**41**	100.0	7.11
**42**	98.0	7.17
**43**	99.4	8.67
**44**	98.6	8.92
**45**	99.6	9.15
**46**	96.3	5.25
**47**	99.3	4.70
**48**	99.5	7.45
**49**	99.3	7.35
**50**	99.7	7.32
**51**	98.9	6.82
**52**	99.7	6.27
**53**	97.6	6.05
**54**	99.6	7.44
**55**	98.0	6.21
**56**	99.6	5.80
**57**	99.4	7.84
**58**	99.0	7.72
**59**	97.6	7.68
**60**	98.7	6.79
**61**	99.4	4.33
**62**	98.9	12.92
**63**	98.1	9.85
**64**	97.1	9.87
**65**	99.4	9.02
**66**	97.7	8.46
**67**	97.6	9.46
**68**	99.8	9.68
**69**	99.9	9.32
**70**	99.8	9.62
**71**	97.2	9.73
**72**	98.0	8.46
**73**	95.1	7.03
**74**	97.7	qNMR
**75**	99.4	12.08
**76**	98.3	6.52
**77**	99.2	6.98
**78**	99.1	6.33
**79**	99.7	6.19
**80**	98.7	6.54
**81**	99.6	6.94
**82**	99.4	8.25
**83**	99.9	8.43
**84**	99.6	7.23
**85**	99.5	8.45
**86**	99.7	7.93
**87**	100.0	8.96
**88**	95.5	8.02
**89**	97.2	8.05
**90**	97.4	7.91
**91**	96.8	7.20
**92**	97.6	7.79
**93**	97.7	7.76
**94**	98.9	17.74
**95**	99.7	7.46

**Table 3:** HRMS data of nusbiarylin compounds.

Compound	Ion formula	m/z (calculated)	m/z (found)
**1**	C_13_H_15_N_2_O_3_ [M − H]^-^	247.1088	247.1089
**2**	C_17_H_11_N_2_O_3_ [M − H]^-^	291.0775	291.0773
**3**	C_13_H_9_N_2_O_3_ [M − H]^-^	241.0619	241.0620
**4**	C_13_H_8_FN_2_O_3_ [M − H]^-^	259.0524	259.0523
**5**	C_13_H_8_FN_2_O_3_ [M − H]^-^	259.0524	259.0528
**6**	C_13_H_8_FN_2_O_3_ [M − H]^-^	259.0524	259.0522
**7**	C_14_H_11_N_2_O_3_ [M − H]^-^	255.0775	255.0771
**8**	C_14_H_11_N_2_O_3_ [M − H]^-^	255.0775	255.0772
**9**	C_14_H_11_N_2_O_3_ [M − H]^-^	255.0775	255.0772
**10**	C_17_H_17_N_2_O_3_ [M − H]^-^	297.1245	297.1247
**11**	C_17_H_17_N_2_O_3_ [M − H]^-^	297.1245	297.1242
**12**	C_17_H_17_N_2_O_3_ [M − H]^-^	297.1245	297.1243
**13**	C_13_H_9_N_2_O_4_ [M − H]^-^	257.0568	257.0565
**14**	C_13_H_9_N_2_O_4_ [M − H]^-^	257.0568	257.0568
**15**	C_13_H_9_N_2_O_4_ [M − H]^-^	257.0568	257.0567
**16**	C_13_H_8_ClN_2_O_3_ [M − H]^-^	275.0229	275.0227
**17**	C_13_H_8_ClN_2_O_3_ [M − H]^-^	275.0229	275.0226
**18**	C_13_H_8_ClN_2_O_3_ [M − H]^-^	275.0229	275.0226
**19**	C_14_H_11_N_2_O_4_ [M − H]^-^	271.0724	271.0719
**20**	C_14_H_11_N_2_O_4_ [M − H]^-^	271.0724	271.0721
**21**	C_14_H_11_N_2_O_4_ [M − H]^-^	271.0724	271.0727
**22**	C_15_H_11_N_2_O_5_ [M − H]^-^	299.0673	299.0672
**23**	C_15_H_11_N_2_O_5_ [M − H]^-^	299.0673	299.0669
**24**	C_15_H_11_N_2_O_5_ [M − H]^-^	299.0673	299.0672
**25**	C_14_H_8_F_3_N_2_O_3_ [M − H]^-^	309.0493	309.0492
**26**	C_14_H_8_F_3_N_2_O_3_ [M − H]^-^	309.0493	309.0491
**27**	C_14_H_8_F_3_N_2_O_3_ [M − H]^-^	309.0493	309.0490
**28**	C_15_H_9_N_2_O_3_ [M − H]^-^	265.0619	265.0618
**29**	C_15_H_9_N_2_O_3_ [M − H]^-^	265.0619	265.0620
**30**	C_15_H_9_N_2_O_3_ [M − H]^-^	265.0619	265.0615
**31**	C_14_H_11_N_2_O_4_ [M − H]^-^	271.0724	271.0721
**32**	C_14_H_11_N_2_O_4_ [M − H]^-^	271.0724	271.0722
**33**	C_14_H_9_N_2_O_5_ [M − H]^-^	285.0517	285.0513
**34**	C_13_H_17_N_2_O_3_ [M − H]^-^	249.1245	249.1245
**35**	C_17_H_13_N_2_O_3_ [M − H]^-^	293.0932	293.0929
**36**	C_13_H_11_N_2_O_3_ [M − H]^-^	243.0775	243.0775
**37**	C_13_H_10_FN_2_O_3_ [M − H]^-^	261.0681	261.0683
**38**	C_13_H_10_FN_2_O_3_ [M − H]^-^	261.0681	261.0679
**39**	C_13_H_10_FN_2_O_3_ [M − H]^-^	261.0681	261.0682
**40**	C_14_H_13_N_2_O_3_ [M − H]^-^	257.0932	257.0928
**41**	C_14_H_13_N_2_O_3_ [M − H]^-^	257.0932	257.0928
**42**	C_14_H_13_N_2_O_3_ [M − H]^-^	257.0932	257.0927
**43**	C_17_H_19_N_2_O_3_ [M − H]^-^	299.1401	299.1401
**44**	C_17_H_19_N_2_O_3_ [M − H]^-^	299.1401	299.1399
**45**	C_17_H_19_N_2_O_3_ [M − H]^-^	299.1401	299.1404
**46**	C_13_H_11_N_2_O_4_ [M − H]^-^	259.0724	259.0723
**47**	C_13_H_11_N_2_O_4_ [M − H]^-^	259.0724	259.0723
**48**	C_13_H_10_ClN_2_O_3_ [M − H]^-^	277.0385	277.0384
**49**	C_13_H_10_ClN_2_O_3_ [M − H]^-^	277.0385	277.0387
**50**	C_13_H_10_ClN_2_O_3_ [M − H]^-^	277.0385	277.0386
**51**	C_14_H_13_N_2_O_4_ [M − H]^-^	273.0881	273.0878
**52**	C_14_H_13_N_2_O_4_ [M − H]^-^	273.0881	273.0876
**53**	C_14_H_13_N_2_O_4_ [M − H]^-^	273.0881	273.0881
**54**	C_15_H_13_N_2_O_5_ [M − H]^-^	301.0830	301.0833
**55**	C_15_H_13_N_2_O_5_ [M − H]^-^	301.0830	301.0831
**56**	C_15_H_13_N_2_O_5_ [M − H]^-^	301.0830	301.0826
**57**	C_14_H_10_F_3_N_2_O_3_ [M − H]^-^	311.0649	311.0651
**58**	C_14_H_10_F_3_N_2_O_3_ [M − H]^-^	311.0649	311.0650
**59**	C_14_H_10_F_3_N_2_O_3_ [M − H]^-^	311.0649	311.0654
**60**	C_15_H_11_N_2_O_3_ [M − H]^-^	267.0775	267.0773
**61**	C_14_H_13_N_2_O_4_ [M − H]^-^	273.0881	273.0879
**62**	C_14_H_11_N_2_O_5_ [M − H]^-^	287.0673	287.0673
**63**	C_17_H_12_NO_3_ [M − H]^-^	278.0823	278.0822
**64**	C_15_H_9_FNO [M − H]^-^	238.0674	238.0672
**65**	C_16_H_9_N_2_O [M − H]^-^	245.0720	245.0719
**66**	C_17_H_11_N_2_O [M − H]^-^	259.0877	259.0876
**67**	C_16_H_12_NO_2_ [M − H]^-^	258.0874	258.0875
**68**	C_15_H_10_NO [M − H]^-^	220.0768	220.0772
**69**	C_15_H_11_N_2_O_2_ [M + H]^+^	251.0815	251.0818
**70**	C_16_H_13_N_2_O_3_ [M + H]^+^	281.0921	281.0922
**71**	C_15_H_9_N_2_O_3_ [M − H]^-^	265.0619	265.0620
**72**	C_15_H_9_N_2_O_3_ [M − H]^-^	265.0619	265.0616
**73**	C_15_H_9_N_2_O_3_ [M − H]^-^	265.0619	265.0621
**74**	C_15_H_8_BrN_2_O_3_ [M − H]^-^	342.9724	342.9727
**75**	C_15_H_8_C_l2_NO [M − H]^-^	287.9988	287.9987
**76**	C_17_H_14_NO_3_ [M − H]^-^	280.0979	280.0974
**77**	C_15_H_11_FNO [M − H]^-^	240.0830	240.0829
**78**	C_16_H_11_N_2_O [M − H]^-^	247.0877	247.0876
**79**	C_17_H_13_N_2_O [M − H]^-^	261.1033	261.1032
**80**	C_16_H_14_NO_2_ [M − H]^-^	252.1030	252.1023
**81**	C_15_H_12_NO [M − H]^-^	222.0924	222.0923
**82**	C_15_H_13_N_2_O_2_ [M + H]^+^	253.0972	253.0974
**83**	C_16_H_15_N_2_O_3_ [M + H]^+^	283.1077	283.1080
**84**	C_15_H_11_N_2_O_3_ [M − H]^-^	267.0775	267.0773
**85**	C_15_H_11_N_2_O_3_ [M − H]^-^	267.0775	267.0770
**86**	C_15_H_10_BrN_2_O_3_ [M − H]^-^	344.9880	344.9878
**87**	C_15_H_10_Cl_2_NO [M − H]^-^	290.0145	290.0141
**88**	C_14_H_10_NO_3_ [M − H]^-^	240.0666	240.0662
**89**	C_15_H_12_NO_4_ [M − H]^-^	270.0772	270.0769
**90**	C_15_H_12_NO_4_ [M − H]^-^	270.0772	270.0771
**91**	C_15_H_9_N_2_O_3_ [M − H]^-^	265.0619	265.0617
**92**	C_16_H_12_NO_5_ [M − H]^-^	298.0721	298.0717
**93**	C_16_H_12_NO_5_ [M − H]^-^	298.0721	298.0719
**94**	C_15_H_9_N_2_O_4_ [M − H]^-^	281.0568	281.0571
**95**	C_13_H_8_NO_4_ [M − H]^-^	242.0459	242.0454

## 2 Experimental design, materials, and methods

### 2.1 HPLC analysis

#### 2.1.1 Sample preparation and HPLC analysis

Approximately 0.1 mg of derivatives were dissolved in 1 mL of HPLC grade acetonitrile. 20 μL of supernatant was manually loaded onto the sample loop. The analysis was carried out on Agilent 1100 series and 1260 infinity system consisting of G1322A degasser, G1311A quat pump and G1365B multi-wavelength detector (MWD). The chromatographic parameters were set as follows:

Mobile phase: Mobile phase A: MeCN, Mobile phase B: H_2_O
Detector: MWD at 254 nm
Column: Agilent ZORBAX Eclipse Plus C18 (4.6 × 100 mm, 5 μm)
Flow rate: 1.000 mL/min
Gradient programme:
For compound **29, 60, 76, 87, 88**

**Table:** 

t/min	Mobile phase A	Mobile phase B
0	30%	70%
2	40%	60%
3	50%	50%
9	80%	20%
14	90%	10%
15	100%	0%
16.5	80%	20%
17	60%	40%
20	30%	70%

For compound **34, 62, 94**

**Table:** 

t/min	Mobile phase A	Mobile phase B
0	10%	90%
8	30%	70%
14	50%	50%
24	100%	0%
25	80%	20%
27	40%	60%
28	10%	90%

For the remaining compounds **except** compounds **22, 24, 27, 33, 74**

**Table:** 

t/min	Mobile phase A	Mobile phase B
0	30%	70%
2	40%	60%
3	50%	50%
13	100%	0%
16.5	30%	70%

#### 2.1.2 Data processing

The automated integration software ChemStation for LC systems B.03.02 [341] was used to acquire the *area under the curve* (mAU). The obtained spectra were then exported as images.

### 2.2 qNMR analysis

#### 2.2.1 Sample preparation and qNMR analysis

Samples were weighed into 5 mm standard NMR tubes using OHAUS® analytical plus balance, followed by addition of 500 μL of DMSO-d_6_ and indicated volume of internal reference 1,3,5-trioxane (99.66% pure, 9.98 mg/mL in DMSO-d_6_) purchased from Dieckmann (Hong Kong) Chemical Industry co., LTD. qNMR analysis were carried out *via* Bruker ultrashield™ NMR spectrometer 600 MHz. NMR instrument controlled parameters were adjusted as follows [5]:

Sample Temperature: 25 °C (298 K, regulated ± 0.1 K)
Data Points (acquired): 64 K
Zero-Filling (SI or FN): to 256 K
Dummy Scans: 4
Relaxation delay (D1): 60 s
Scans (NS or NT): 16

#### 2.2.2 Data processing

The software Bruker topspin 3.2 was used to acquire the integrals of the signals of sample and internal reference. The normalized integrals values per proton equivalent by dividing each integral by the corresponding number of protons were calculated, so as the integral of the analyte (***Int***_***t***_ and ***Int***_***IC***_) as the average of all normalized integrals. The total number of protons (***n***_***t***_ and ***n***_***IC***_) was set to one [5]. Purities was then calculated according to the equation as below:

$$P\;\left[\%\right]=\;\frac{n_{IC}*Int_{t}*MW_{t}*m_{IC}}{n_{t}*Int_{IC}*MW_{IC}*m_{s}}*P_{IC}$$

where: ***P*** = purity of tested compound

***m***_***IC***_ = weight of the internal calibrant (IC)
***m***_***s***_ = weight of the sample
***Int***_***IC***_ = integral of the IC resonance signal being used for quantification
***Int***_***t***_ = integral of the target analyte (t) resonance signal being used for quantification
***n***_***IC***_ = number of protons that give rise to ***Int***_***IC***_
***n***_***t***_ = number of protons of the target analyte that give rise to ***Int***_***t***_
***MW***_***IC***_ = molecular weight of the internal calibrant
***MW***_***t***_ = molecular weight of the target analyte
***P***_***IC***_ = purity of the internal calibrant, as percent value

### 2.3 HRMS analysis for all compounds

#### 2.3.1 Sample preparation and HRMS analysis

Approximately 0.1 mg of derivatives were dissolved in 1 mL of HPLC grade acetonitrile. After sonication and filtration *via* 0.22 μm PTFE syringe filter, 10 μL of the upper layer was injected using an autosampler onto Agilent Technologies 6520 Accurate-Mass Q-TOF LC/MS spectrometer. The spectrometer was calibrated before each chromatographic run for optimal mass accuracy. The mobile phase gradient was 100% acetonitrile, at a flow rate of 0.5 ml/min. The mass spectra were acquired in positive- or negative-ion mode with source temperature at 300 °C. Ion spray voltage and fragmentor voltage were adjusted to 3.5 kV and 175 V, respectively. The range of mass detected was between 100 m/z and 1000 m/z.

#### 2.3.2 Data processing

HRMS profiles were acquired and processed using MassHunter B.07 software. The obtained spectra were then exported as images.
