# Supplementary material for: HPLC, quantitative NMR and HRMS spectroscopic data of nusbiarylins as a new class of antimicrobial agents
Source: Data Brief. 2020 Feb 21;29:105313. doi: 10.1016/j.dib.2020.105313 (PMC7056622; doi:10.1016/j.dib.2020.105313)
Supplement: Multimedia component 1 [file mmc1.pdf]

HPLC, quantitative NMR and HRMS spectroscopic data of nusbiarylins as a new class of antimicrobial agents

Authors

Yangyi Qiu, Cong Ma*

Affiliations

State Key Laboratory of Chemical Biology and Drug Discovery, Department of Applied Biology and Chemical Technology, The Hong Kong Polytechnic University, Kowloon, Hong Kong SAR

Corresponding author

Cong Ma (cong.ma@polyu.edu.hk)

Table of Contents

HPLC spectra	…………………………………………………………………	S2-S53
qNMR spectra	…………………………………………………………………	S54-S58
HRMS spectra	…………………………………………………………………	S59-S90

HPLC profiles

1

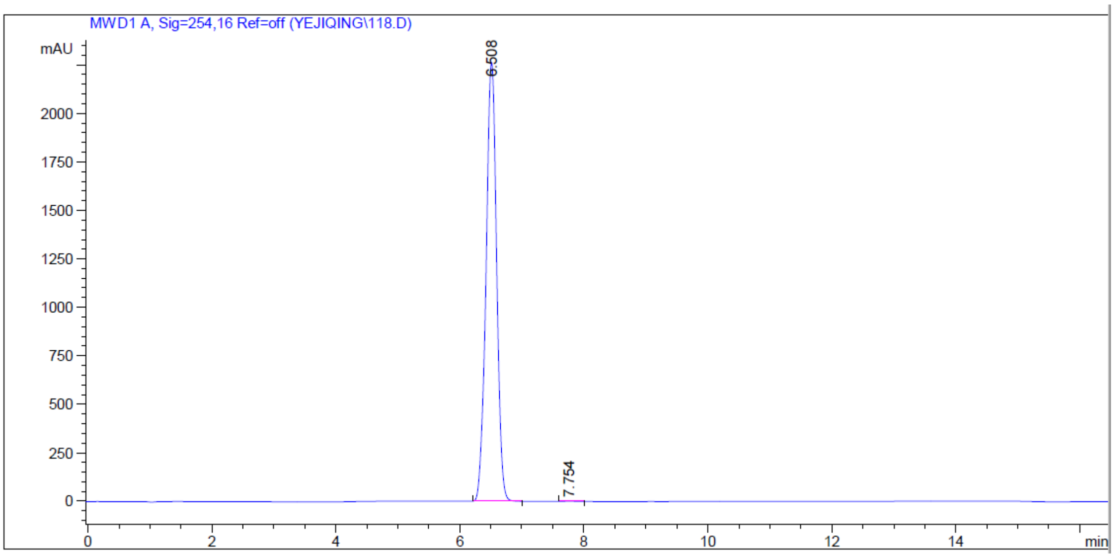

Peak #	RetTime [min]	Type	Width [min]	Area [mAU*s]	Height [mAU]	Area %
1	6.508	BB	0.1801	2.68071e4	2264.17090	99.9182
2	7.754	BB	0.1754	21.95600	1.86420	0.0818

Totals : 2.68291e4 2266.03510

2

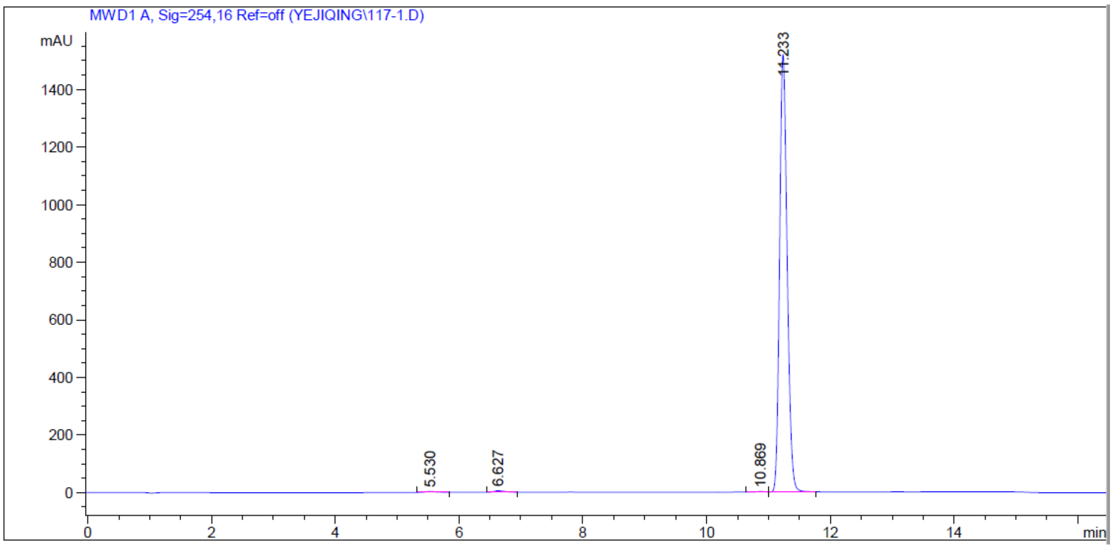

Peak #	RetTime [min]	Type	Width [min]	Area [mAU*s]	Height [mAU]	Area %
1	5.530	BB	0.1752	42.69644	3.47751	0.3271
2	6.627	BB	0.1870	68.45049	5.50904	0.5245
3	10.869	BV	0.1495	18.46266	1.80256	0.1415
4	11.233	VB	0.1311	1.29215e4	1521.35730	99.0069

Totals : 1.30511e4 1532.14641

3

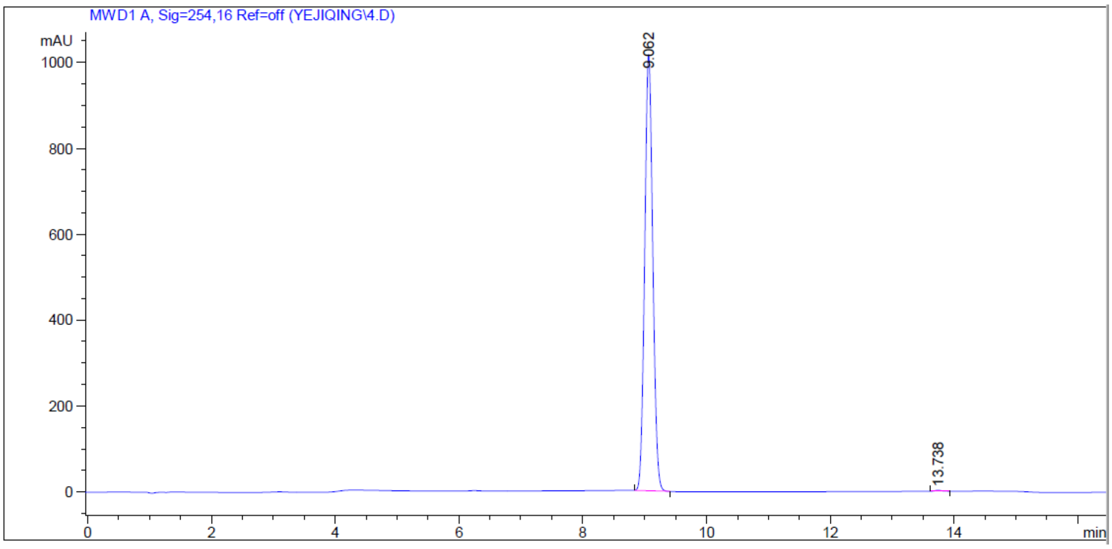

Peak #	RetTime [min]	Type	Width [min]	Area [mAU*s]	Height [mAU]	Area %
1	9.062	BB	0.1401	9249.13379	1016.98688	99.8225
2	13.738	BB	0.1086	16.44216	2.32368	0.1775

Totals : 9265.57594 1019.31056

4

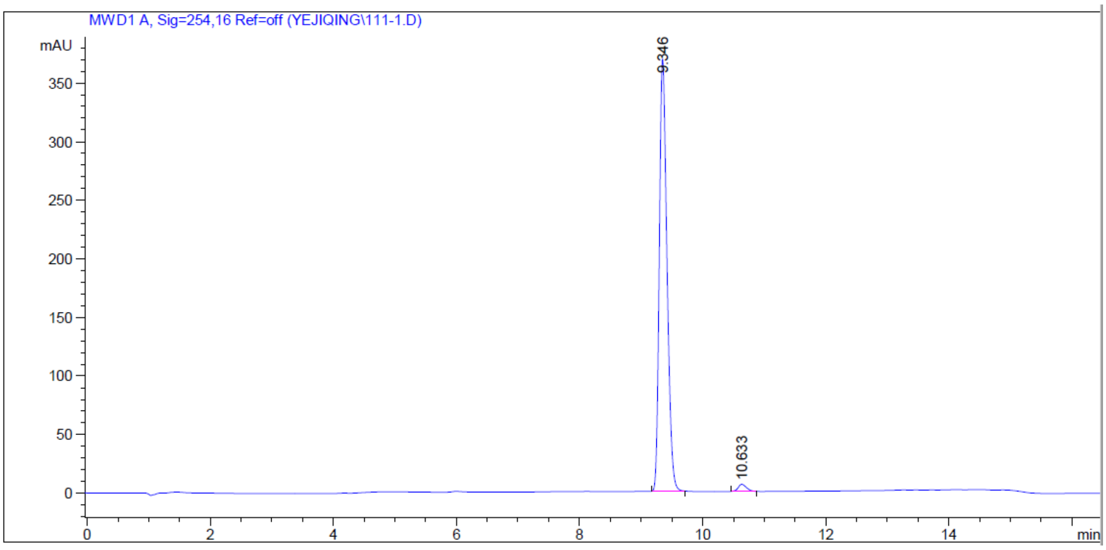

Peak #	RetTime [min]	Type	Width [min]	Area [mAU*s]	Height [mAU]	Area %
1	9.346	BB	0.1330	3196.63843	369.31638	98.1573
2	10.633	BB	0.1470	60.01086	6.19676	1.8427

Totals : 3256.64929 375.51314

5

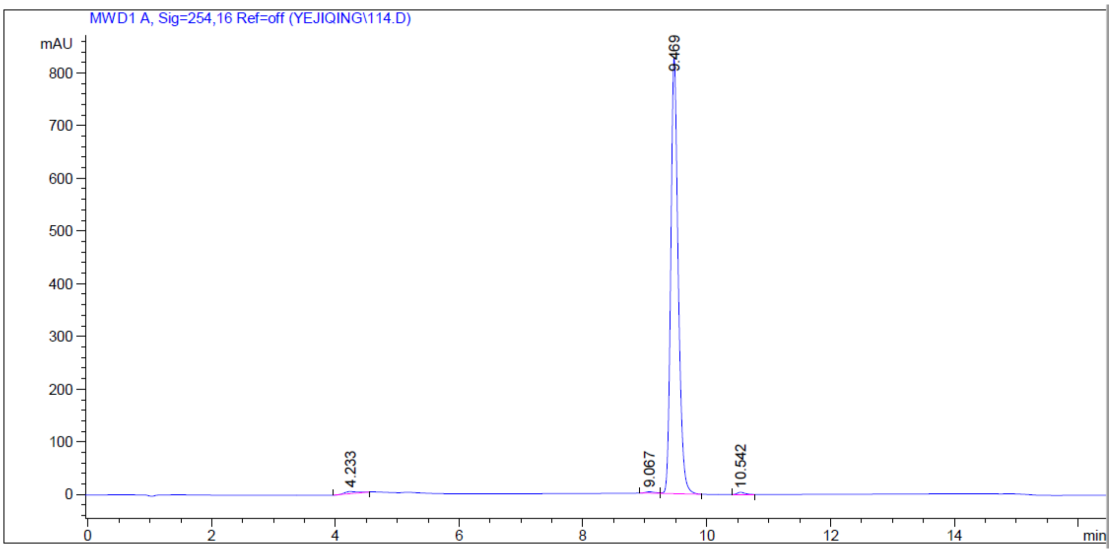

Peak #	RetTime [min]	Type	Width [min]	Area [mAU*s]	Height [mAU]	Area %
1	4.233	BB	0.2245	67.76752	4.33106	0.9587
2	9.067	BV	0.1685	37.04250	3.16657	0.5240
3	9.469	VB	0.1255	6927.30908	828.76733	97.9964
4	10.542	BB	0.1284	36.82264	4.27994	0.5209

Totals : 7068.94175 840.54490

6

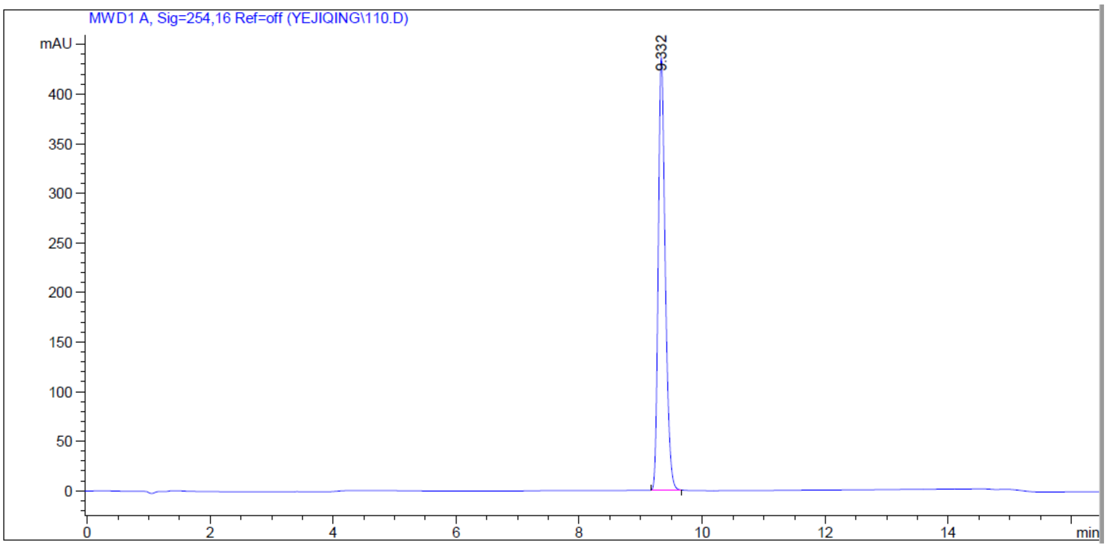

Peak #	RetTime [min]	Type	Width [min]	Area [mAU*s]	Height [mAU]	Area %
1	9.332	BB	0.1187	3474.49951	437.11560	100.0000

Totals : 3474.49951 437.11560

7

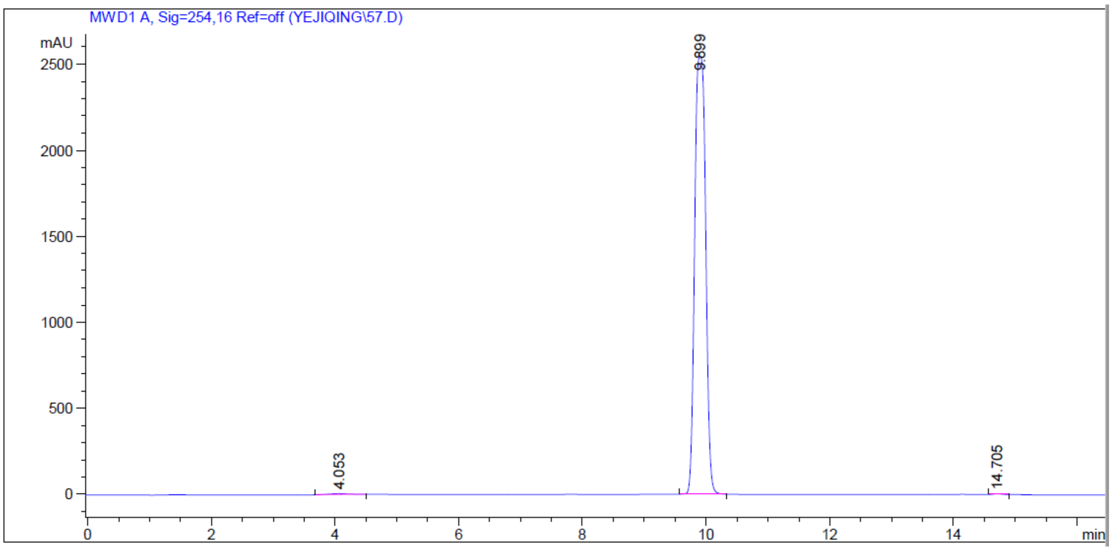

Peak #	RetTime [min]	Type	Width [min]	Area [mAU*s]	Height [mAU]	Area %
1	4.053	BB	0.3321	124.28331	5.44856	0.3959
2	9.899	BB	0.1971	3.12329e4	2546.27808	99.4796
3	14.705	BB	0.1239	39.09536	4.85806	0.1245

Totals : 3.13963e4 2556.58470

8

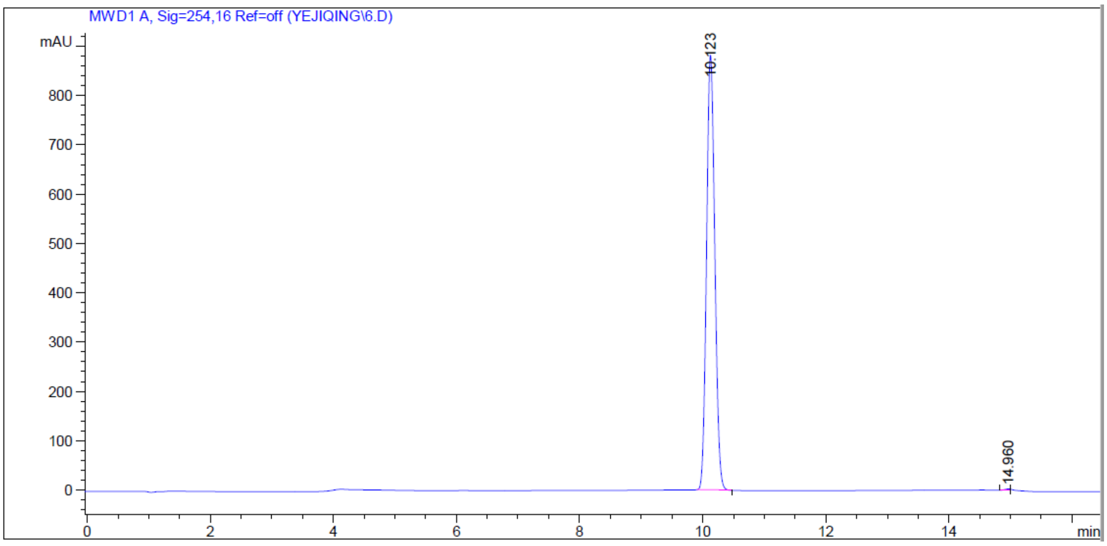

Peak #	RetTime [min]	Type	Width [min]	Area [mAU*s]	Height [mAU]	Area %
1	10.123	BB	0.1450	8241.99902	882.45172	99.8289
2	14.960	BBA	0.0950	14.12340	2.47694	0.1711

Totals : 8256.12243 884.92866

9

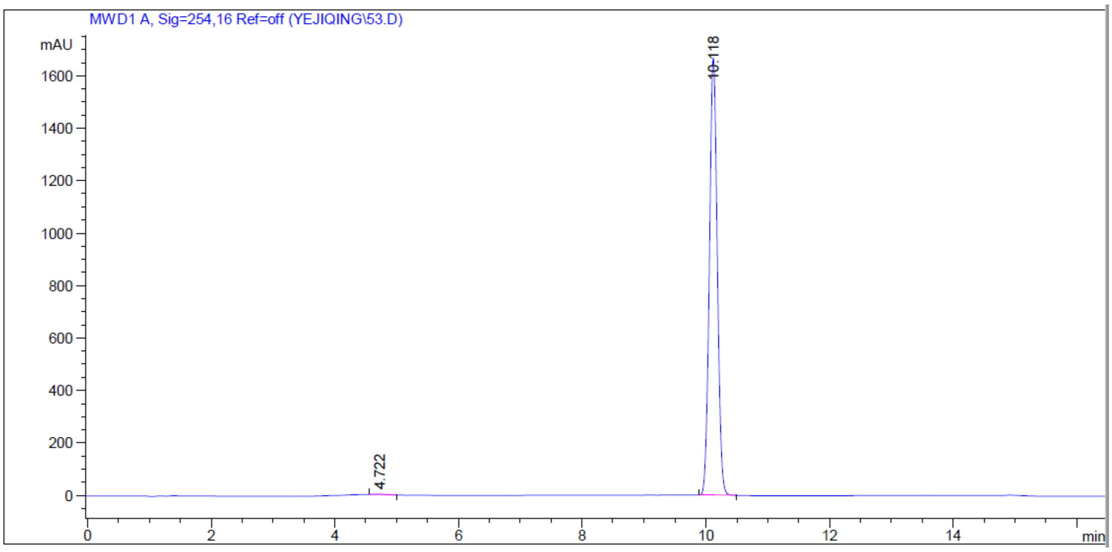

Peak #	RetTime [min]	Type	Width [min]	Area [mAU*s]	Height [mAU]	Area %
1	4.722	BB	0.1580	22.01364	2.21415	0.1489
2	10.118	BB	0.1352	1.47606e4	1668.66479	99.8511

Totals : 1.47826e4 1670.87894

10

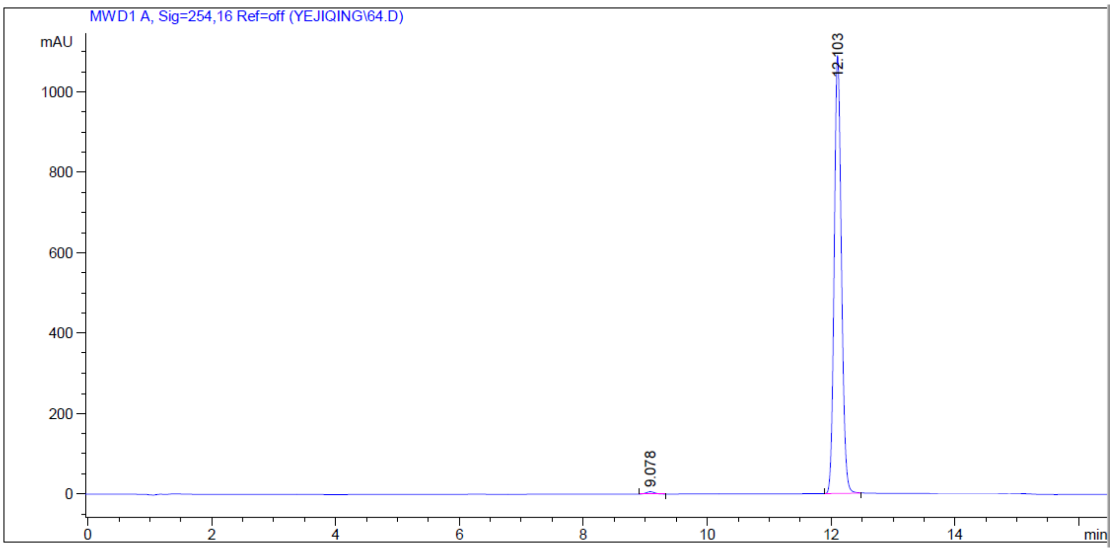

Peak #	RetTime [min]	Type	Width [min]	Area [mAU*s]	Height [mAU]	Area %
1	9.078	BB	0.1463	56.42135	5.86150	0.6366
2	12.103	BB	0.1242	8806.21094	1090.55762	99.3634

Totals : 8862.63229 1096.41912

11

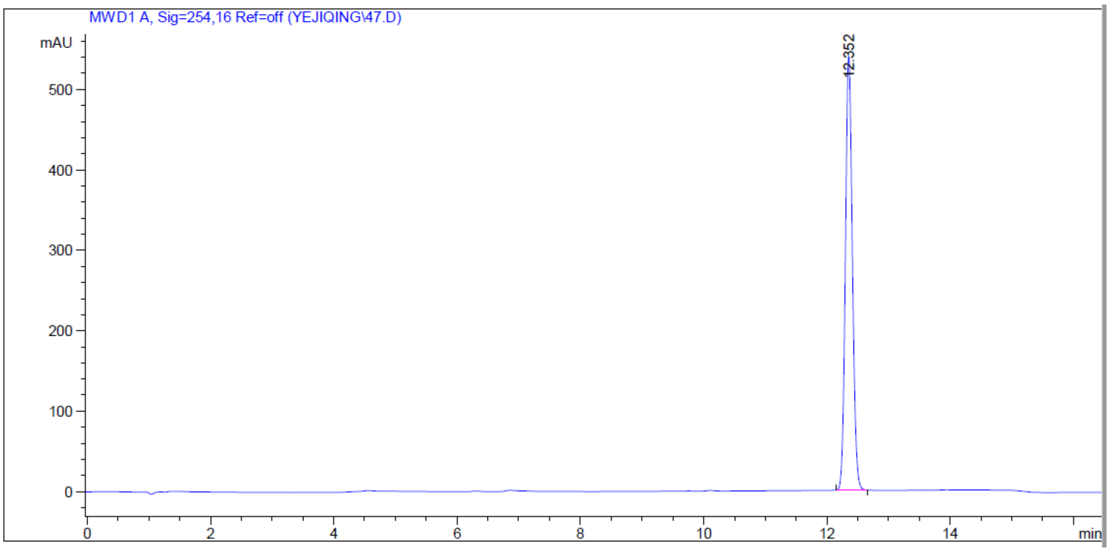

Peak #	RetTime [min]	Type	Width [min]	Area [mAU*s]	Height [mAU]	Area %
1	12.352	BB	0.1197	4248.96875	540.57526	100.0000

Totals : 4248.96875 540.57526

12

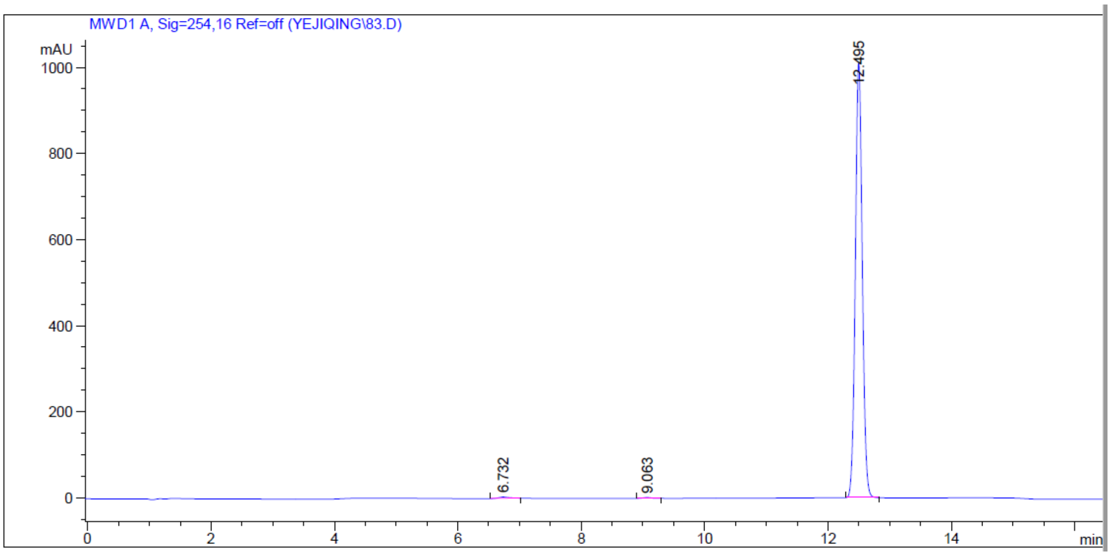

Peak #	RetTime [min]	Type	Width [min]	Area [mAU*s]	Height [mAU]	Area %
1	6.732	BB	0.1890	43.61917	3.32616	0.5453
2	9.063	BB	0.1465	20.16895	2.05532	0.2521
3	12.495	BB	0.1215	7936.04883	1011.64221	99.2026

Totals : 7999.83696 1017.02368

13

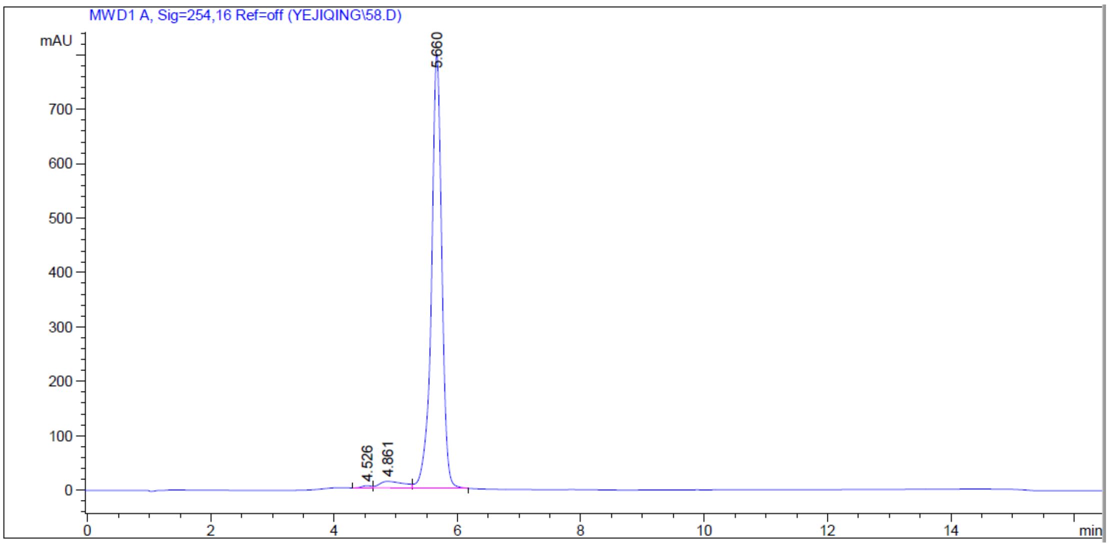

Peak #	RetTime [min]	Type	Width [min]	Area [mAU*s]	Height [mAU]	Area %
1	4.526	BV	0.1698	45.75504	4.17751	0.4703
2	4.861	VV	0.4109	343.29517	12.32845	3.5285
3	5.660	VB	0.1785	9340.16113	798.48242	96.0012

Totals : 9729.21134 814.98838

14

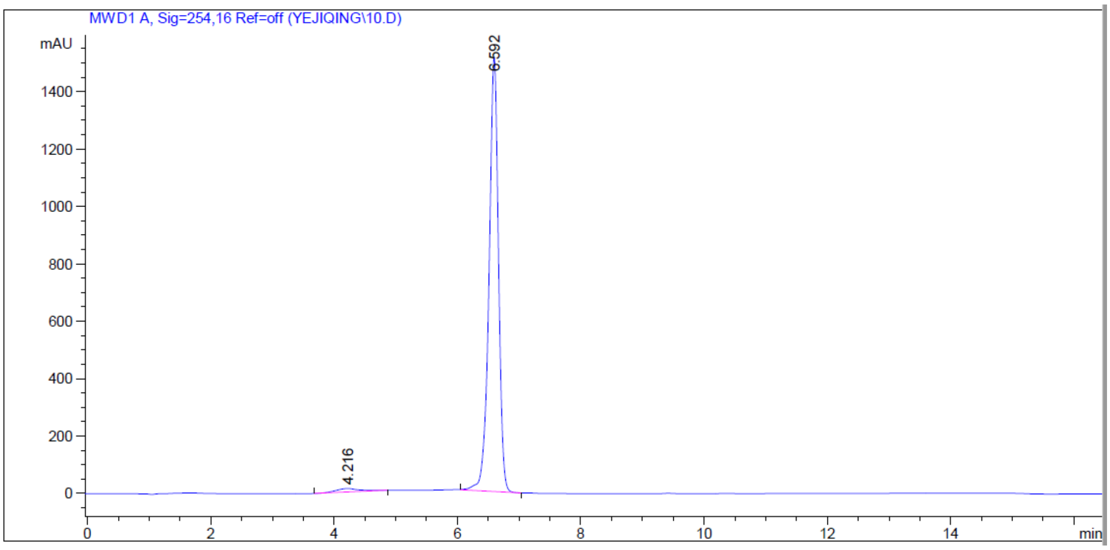

Peak #	RetTime [min]	Type	Width [min]	Area [mAU*s]	Height [mAU]	Area %
1	4.216	BB	0.4132	357.84616	12.60684	2.1658
2	6.592	BB	0.1606	1.61651e4	1514.00464	97.8342

Totals : 1.65230e4 1526.61148

15

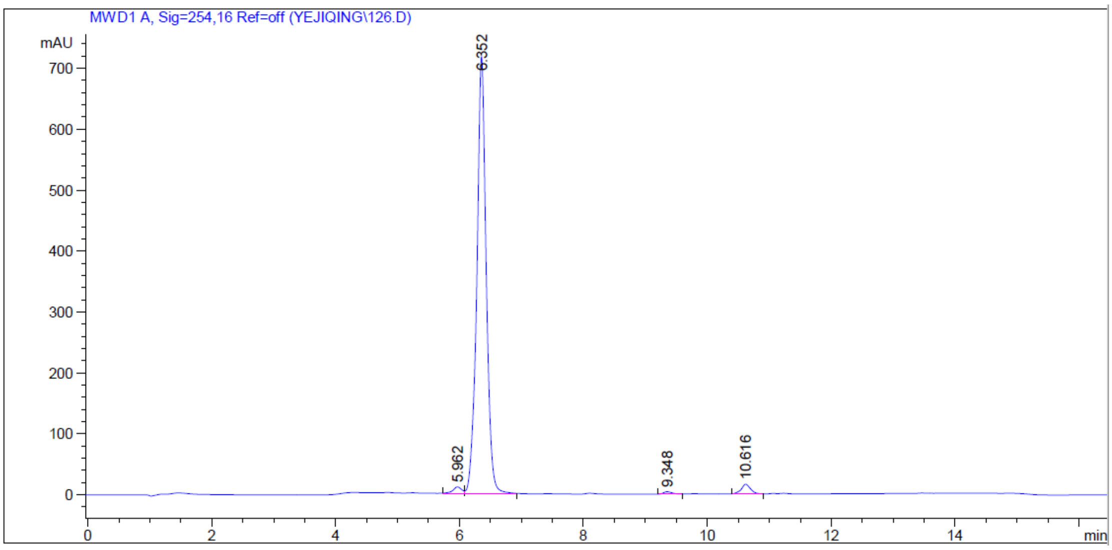

Peak #	RetTime [min]	Type	Width [min]	Area [mAU*s]	Height [mAU]	Area %
1	5.962	BV	0.1440	103.42542	10.58717	1.3173
2	6.352	VB	0.1526	7542.95996	717.51581	96.0709
3	9.348	BB	0.1457	36.20859	3.92266	0.4612
4	10.616	BB	0.1552	168.85608	15.99008	2.1506

Totals : 7851.45004 748.01573

16

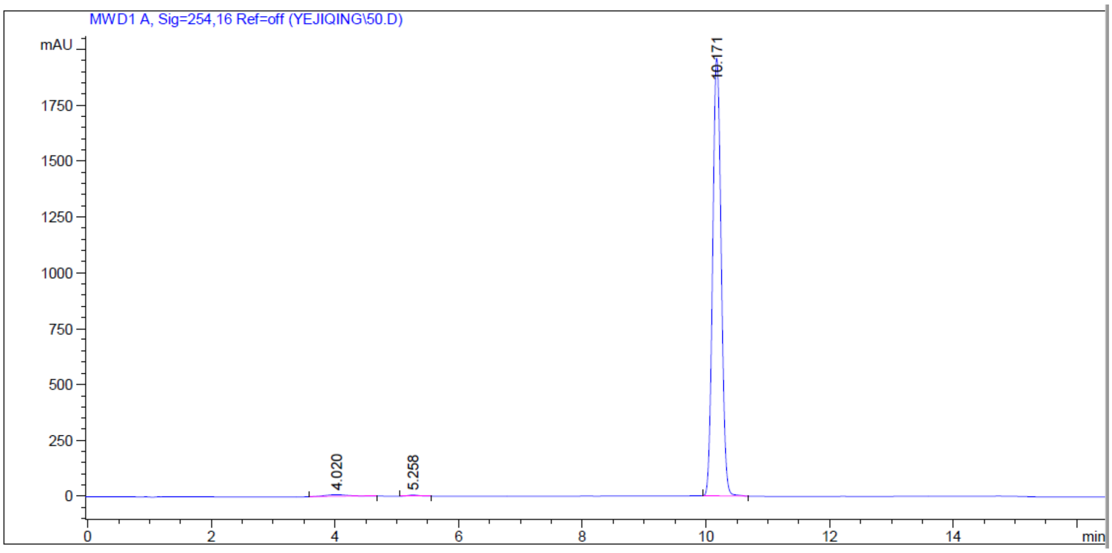

Peak #	RetTime [min]	Type	Width [min]	Area [mAU*s]	Height [mAU]	Area %
1	4.020	BB	0.4242	225.32585	8.05838	1.1807
2	5.258	BB	0.1801	60.72013	5.20481	0.3182
3	10.171	BB	0.1498	1.87979e4	1960.83301	98.5011

Totals : 1.90839e4 1974.09620

17

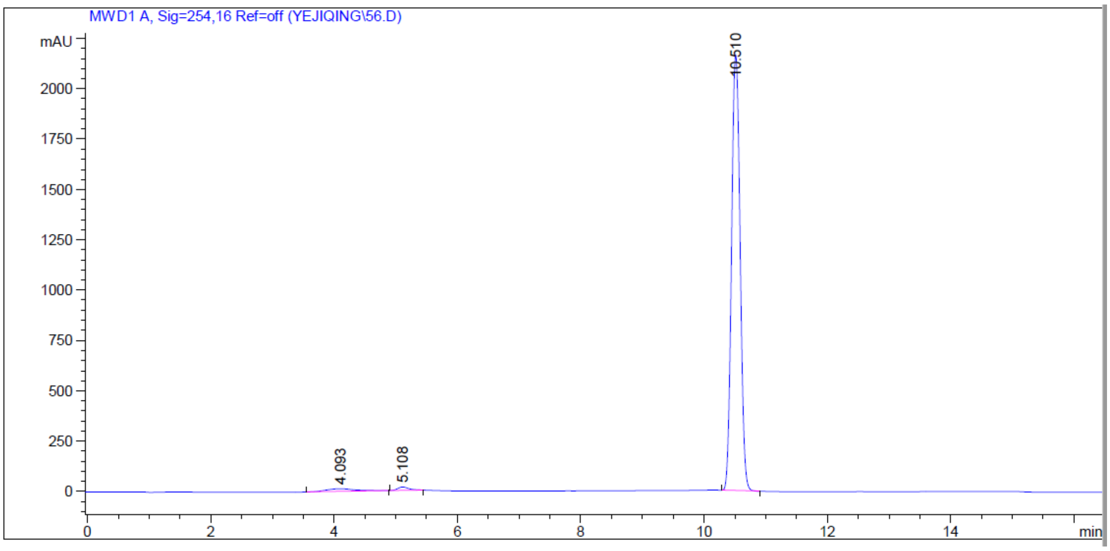

Peak #	RetTime [min]	Type	Width [min]	Area [mAU*s]	Height [mAU]	Area %
1	4.093	BB	0.5305	491.51706	13.96918	2.2649
2	5.108	BB	0.2039	250.12196	18.02782	1.1525
3	10.510	BB	0.1550	2.09603e4	2164.75269	96.5826
Totals :				2.17019e4	2196.74969	

18

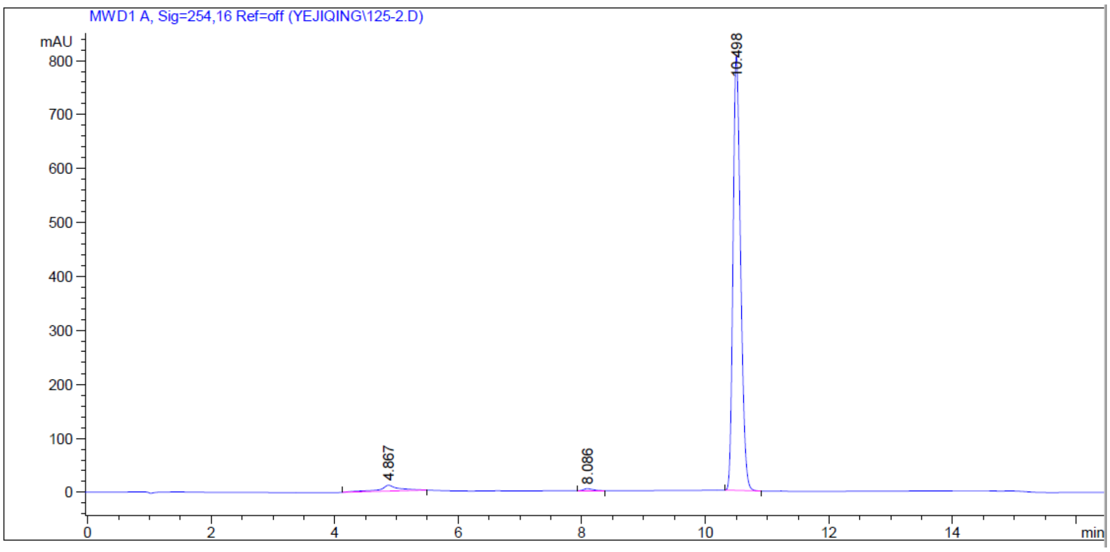

Peak #	RetTime [min]	Type	Width [min]	Area [mAU*s]	Height [mAU]	Area %
1	4.867	BB	0.3169	279.94778	11.46316	3.9024
2	8.086	BB	0.1552	39.49755	3.80260	0.5506
3	10.498	BB	0.1291	6854.30518	807.03595	95.5470
Totals :				7173.75051	822.30170	

19

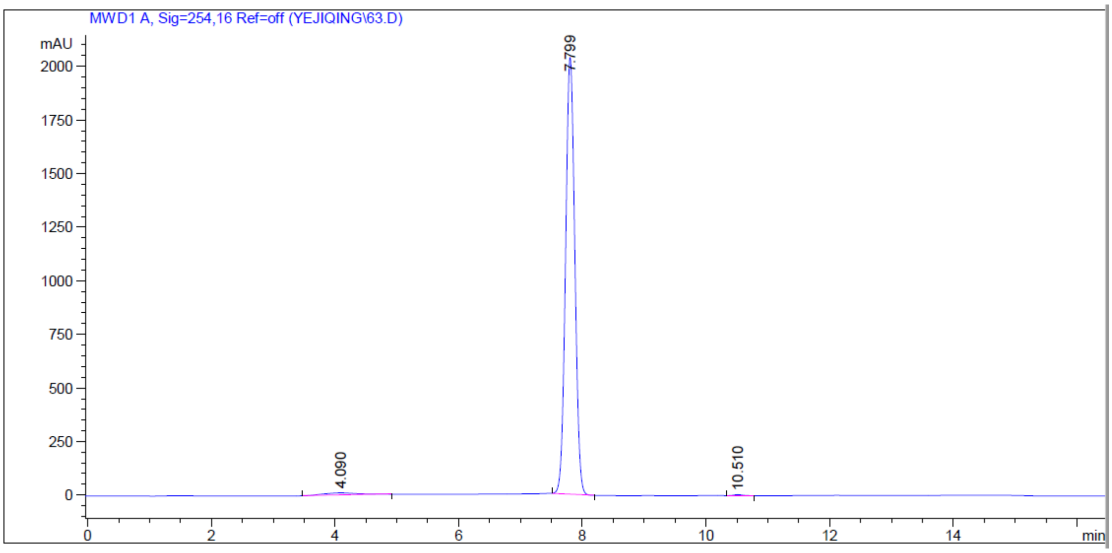

Peak #	RetTime [min]	Type	Width [min]	Area [mAU*s]	Height [mAU]	Area %
1	4.090	BB	0.5550	425.77118	10.68143	1.9714
2	7.799	BB	0.1629	2.11273e4	2038.78613	97.8250
3	10.510	BB	0.1458	43.97246	4.67042	0.2036

Totals : 2.15970e4 2054.13799

20

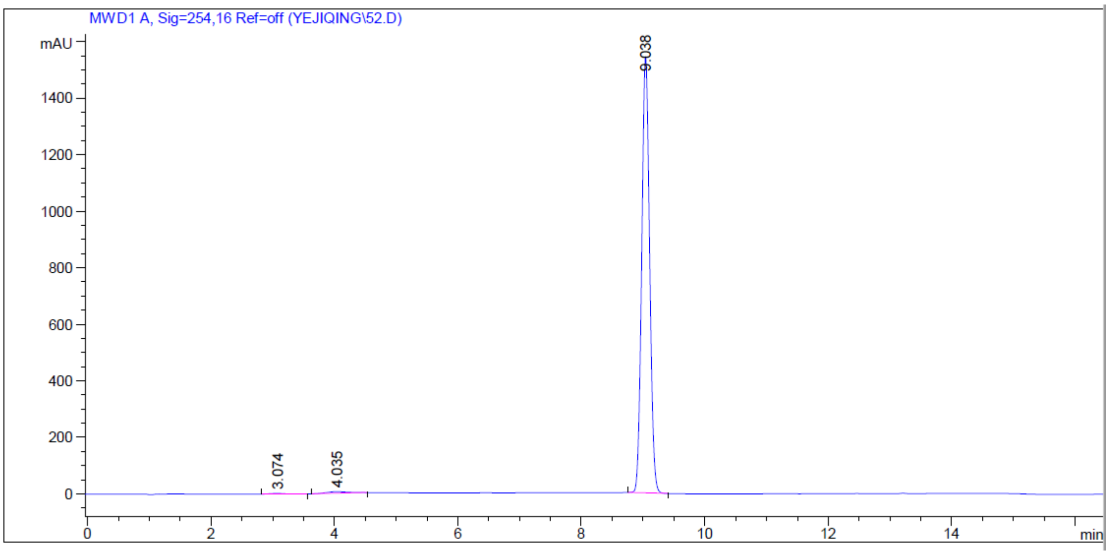

Peak #	RetTime [min]	Type	Width [min]	Area [mAU*s]	Height [mAU]	Area %
1	3.074	BB	0.2499	30.58688	2.02848	0.2197
2	4.035	BB	0.3693	154.22217	6.01239	1.1078

3	9.038	BB	0.1378	1.37365e4	1544.48218	98.6725

Totals :	1.39213e4	1552.52305

21

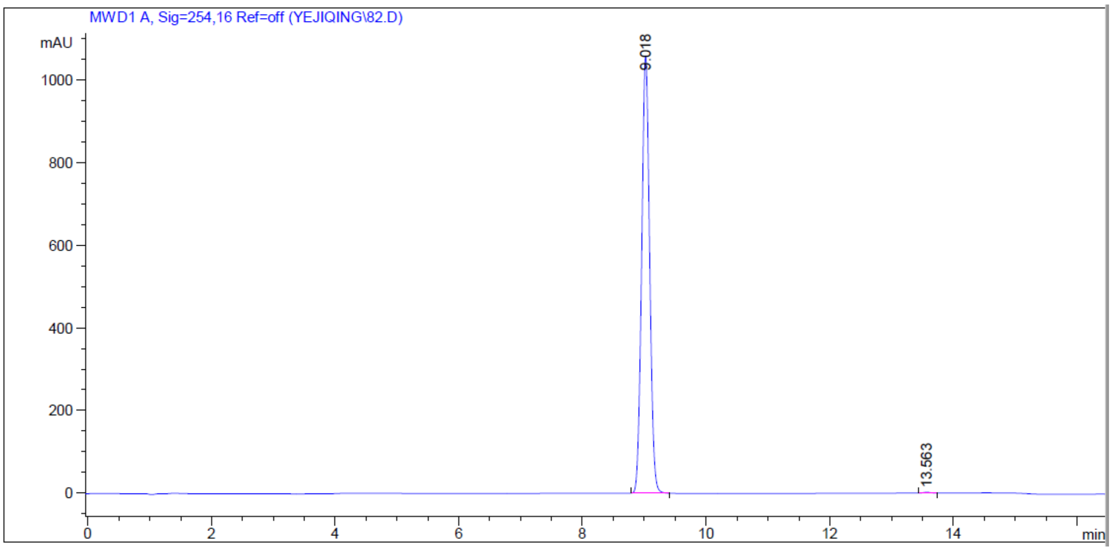

Peak #	RetTime [min]	Type	Width [min]	Area [mAU*s]	Height [mAU]	Area %
1	9.018	BB	0.1367	9488.60352	1057.69934	99.8520
2	13.563	BB	0.1087	14.06204	1.98282	0.1480
Totals :				9502.66555	1059.68216	

23

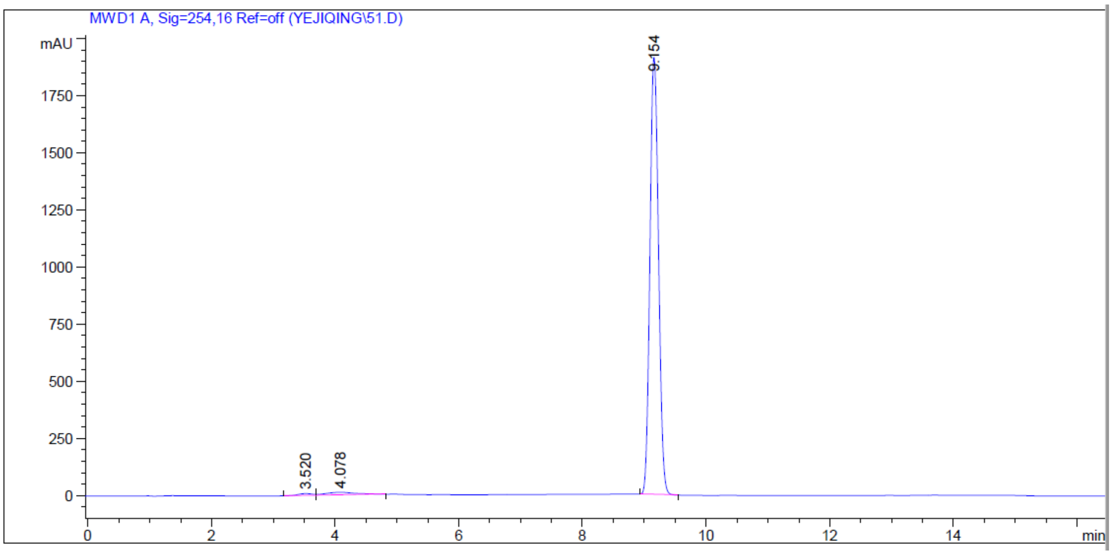

Peak #	RetTime [min]	Type	Width [min]	Area [mAU*s]	Height [mAU]	Area %
1	3.520	BV	0.2591	134.85159	8.23069	0.7084
2	4.078	VB	0.5168	405.18393	11.62005	2.1286
3	9.154	BB	0.1507	1.84951e4	1914.24658	97.1630

Totals : 1.90351e4 1934.09732

25

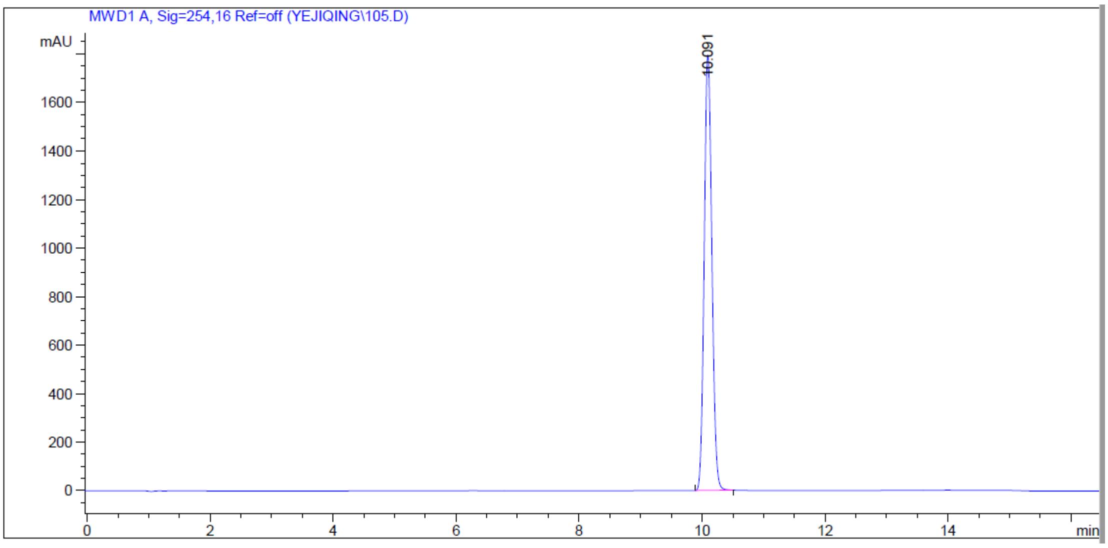

Peak #	RetTime [min]	Type	Width [min]	Area [mAU*s]	Height [mAU]	Area %
1	10.091	BB	0.1350	1.55551e4	1797.07727	100.0000

Totals : 1.55551e4 1797.07727

26

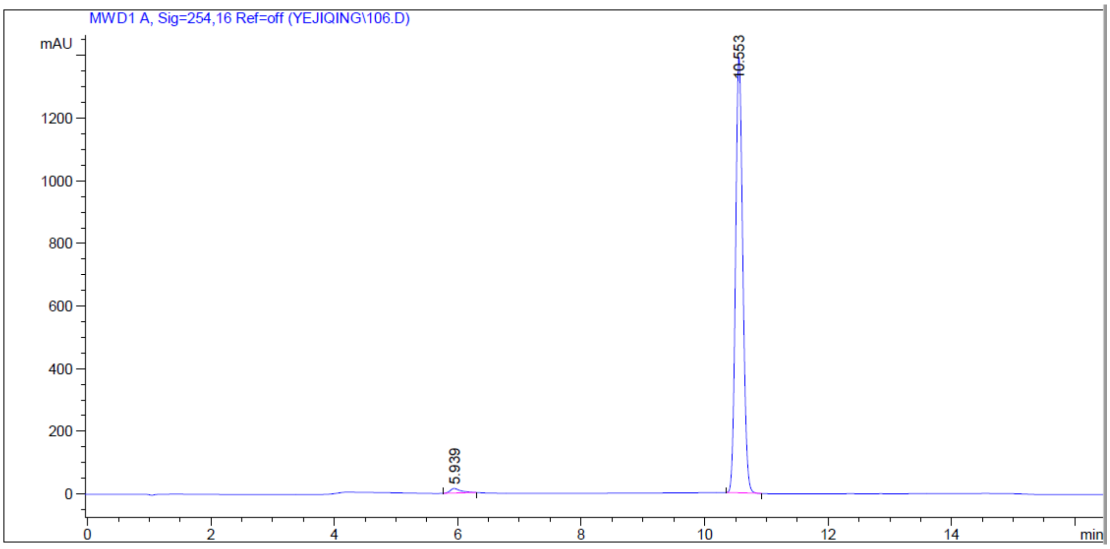

Peak #	RetTime [min]	Type	Width [min]	Area [mAU*s]	Height [mAU]	Area %
1	5.939	BB	0.1831	195.26025	15.07143	1.6972
2	10.553	BB	0.1248	1.13099e4	1391.73352	98.3028

Totals : 1.15051e4 1406.80495

28

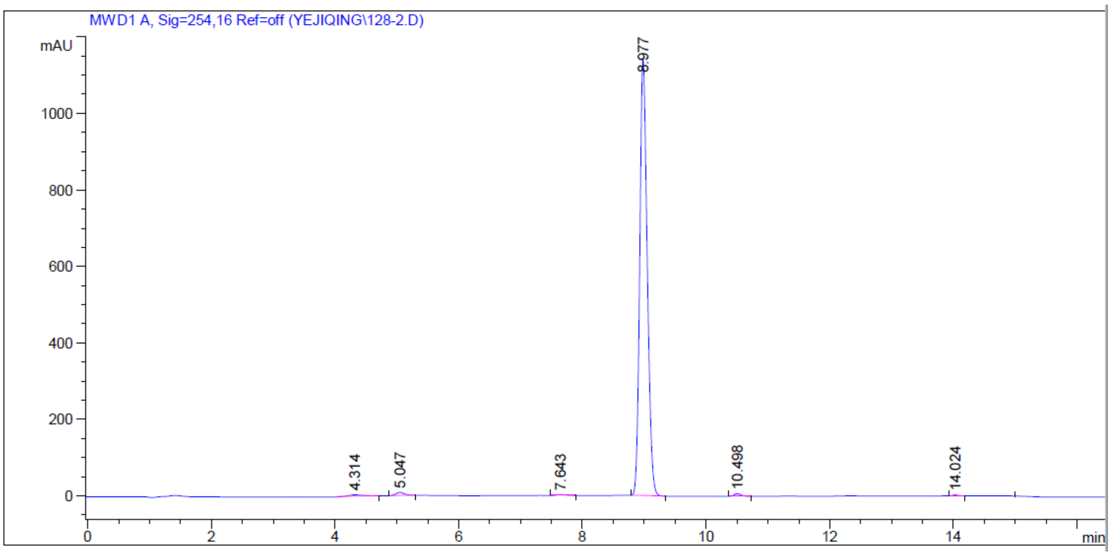

Peak #	RetTime [min]	Type	Width [min]	Area [mAU*s]	Height [mAU]	Area %
1	4.314	BB	0.2583	79.30613	4.44111	0.8037
2	5.047	BB	0.1477	86.35835	8.55815	0.8751
3	7.643	BB	0.1435	22.53098	2.35702	0.2283
4	8.977	BB	0.1280	9610.18457	1144.34167	97.3858
5	10.498	BB	0.1252	55.34827	6.92899	0.5609
6	14.024	BB	0.0859	14.43415	2.55397	0.1463

Totals : 9868.16246 1169.18091

29

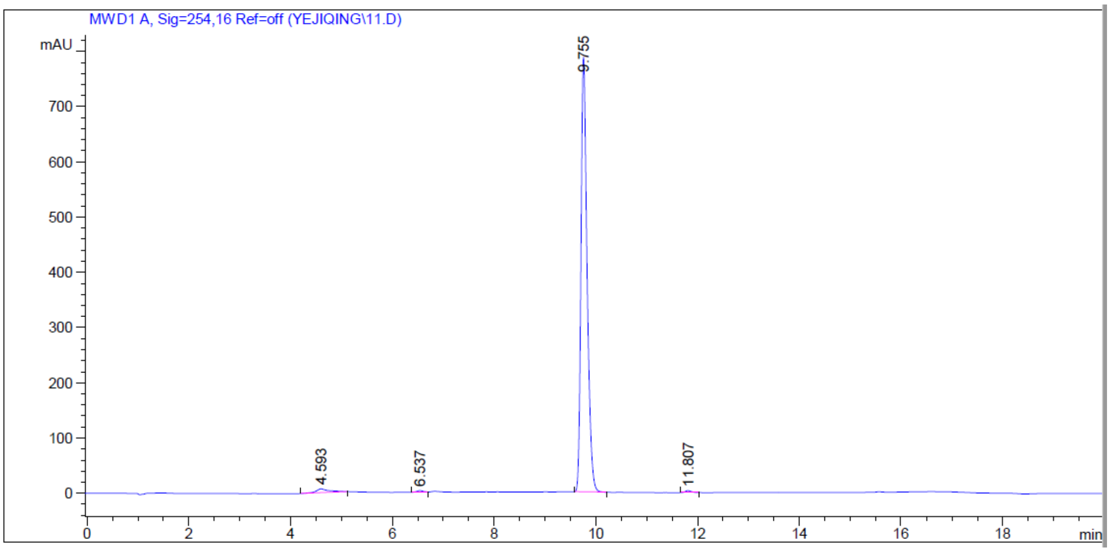

Peak #	RetTime [min]	Type	Width [min]	Area [mAU*s]	Height [mAU]	Area %
1	4.593	BB	0.2616	141.07434	7.16786	2.0858
2	6.537	BV	0.1267	27.23679	3.09321	0.4027
3	9.755	BB	0.1231	6563.89502	788.66595	97.0491
4	11.807	BB	0.1301	31.27031	3.64517	0.4623
Totals :				6763.47646	802.57219	

30

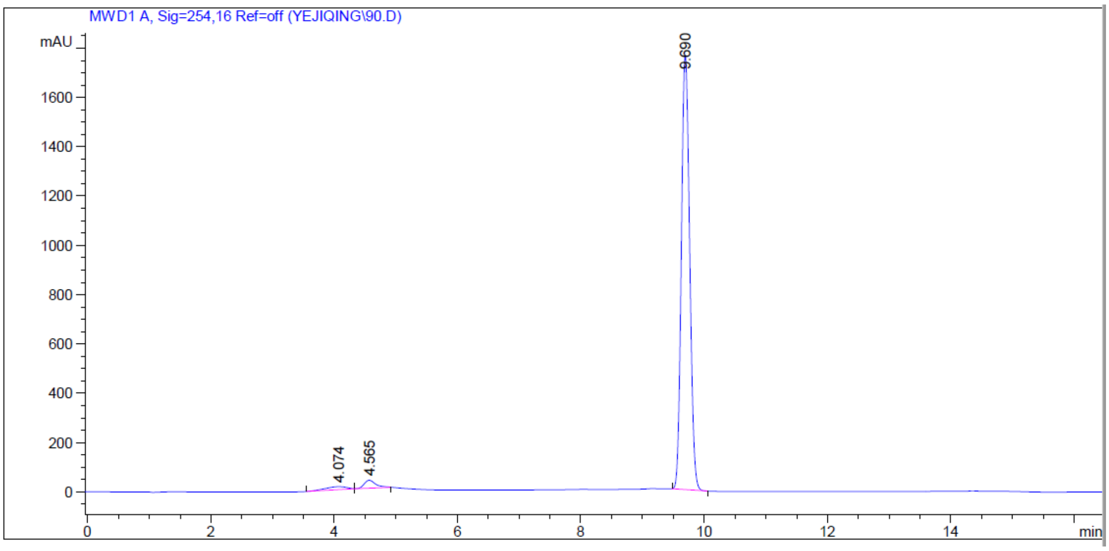

Peak #	RetTime [min]	Type	Width [min]	Area [mAU*s]	Height [mAU]	Area %
1	4.074	BV	0.3957	334.78143	13.20981	1.9337
2	4.565	VB	0.2048	451.34854	33.15677	2.6070
3	9.690	BB	0.1493	1.65270e4	1764.68457	95.4593

Totals : 1.73131e4 1811.05115

31

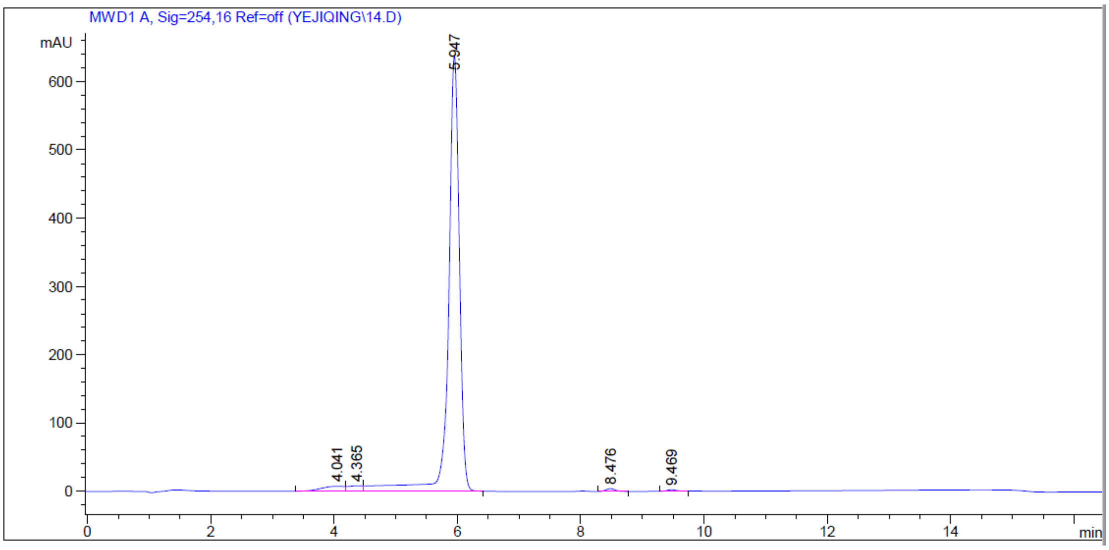

Peak #	RetTime [min]	Type	Width [min]	Area [mAU*s]	Height [mAU]	Area %
1	4.041	BV	0.3931	188.61693	7.21192	2.2698
2	4.365	VV	0.2403	134.89429	7.93551	1.6233
3	5.947	VB	0.1887	7923.08789	638.86536	95.3459
4	8.476	BB	0.1640	41.75369	4.06027	0.5025
5	9.469	BB	0.1569	21.48521	2.14338	0.2586

Totals : 8309.83801 660.21644

32

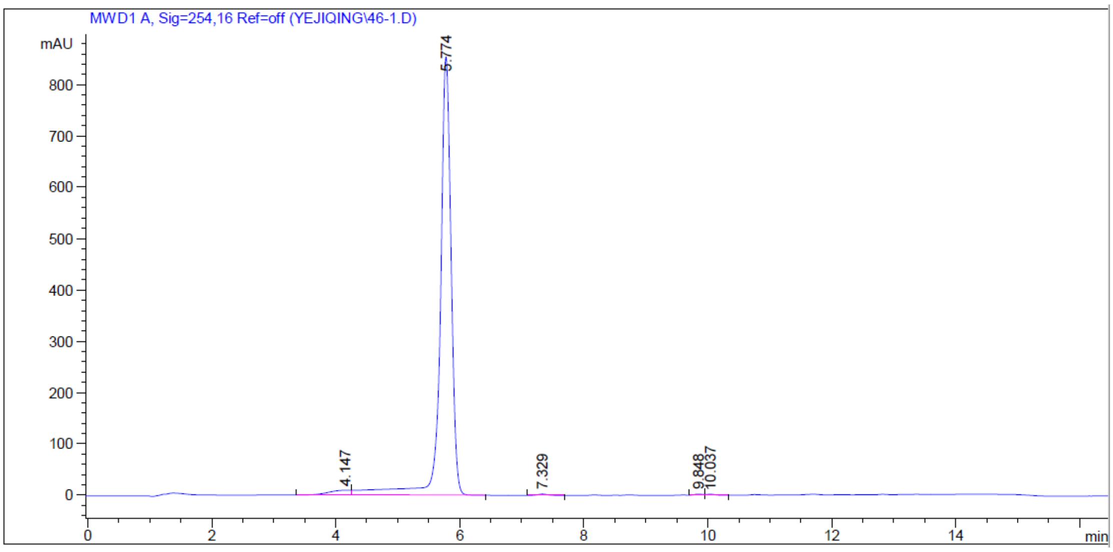

Peak #	RetTime [min]	Type	Width [min]	Area [mAU*s]	Height [mAU]	Area %
1	4.147	BV	0.3856	231.77194	9.27217	2.1272
2	5.774	VB	0.1865	1.05883e4	854.90625	97.1779
3	7.329	BB	0.1689	31.29954	2.74699	0.2873
4	9.848	VV	0.1484	23.54970	2.40226	0.2161
5	10.037	VB	0.1523	20.86700	2.09372	0.1915

Totals : 1.08958e4 871.42139

34

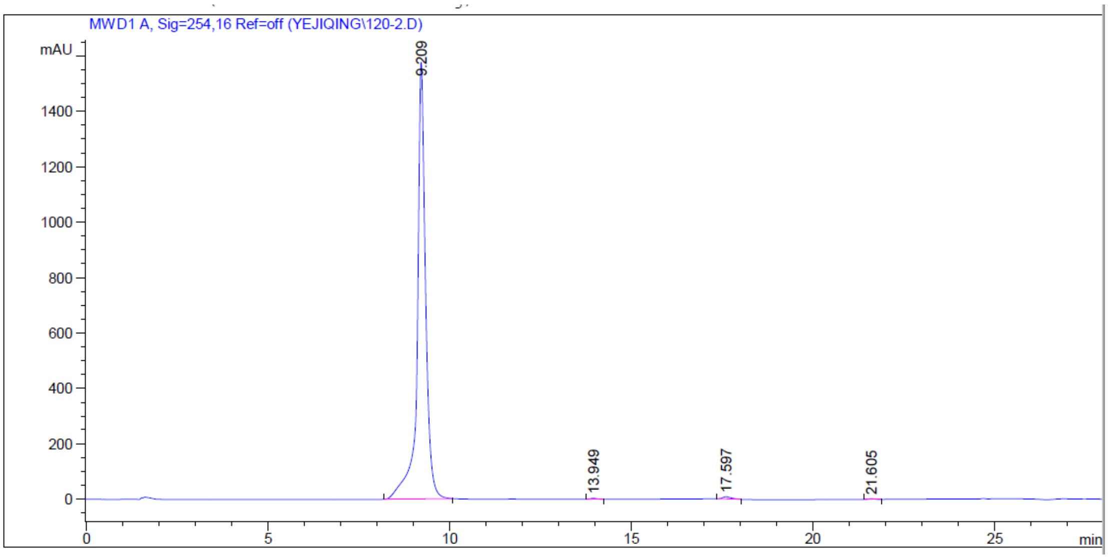

Peak #	RetTime [min]	Type	Width [min]	Area [mAU*s]	Height [mAU]	Area %
1	9.209	BB	0.2286	2.52275e4	1577.27869	99.1920
2	13.949	BB	0.1683	41.38209	3.82137	0.1627
3	17.597	BB	0.2459	137.82053	8.20890	0.5419
4	21.605	BB	0.1667	26.30022	2.50155	0.1034
Totals :				2.54330e4	1591.81050	

35

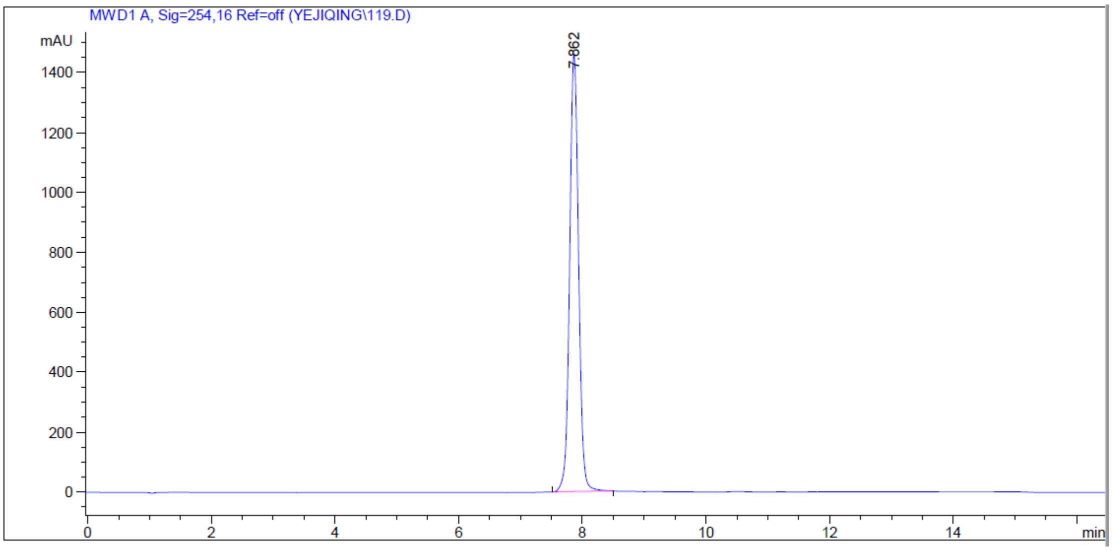

Peak #	RetTime [min]	Type	Width [min]	Area [mAU*s]	Height [mAU]	Area %
1	7.862	BB	0.1556	1.47201e4	1460.42957	100.0000
Totals :				1.47201e4	1460.42957	

36

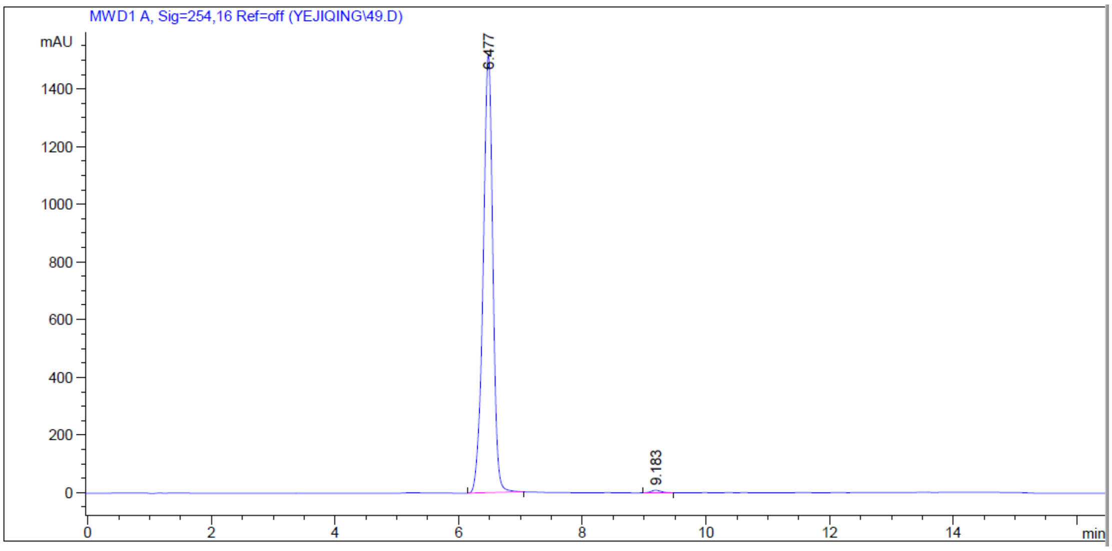

Peak #	RetTime [min]	Type	Width [min]	Area [mAU*s]	Height [mAU]	Area %
1	6.477	BB	0.1673	1.65908e4	1519.97021	99.3885
2	9.183	BB	0.1710	102.08523	8.95638	0.6115
Totals :				1.66929e4	1528.92659	

37

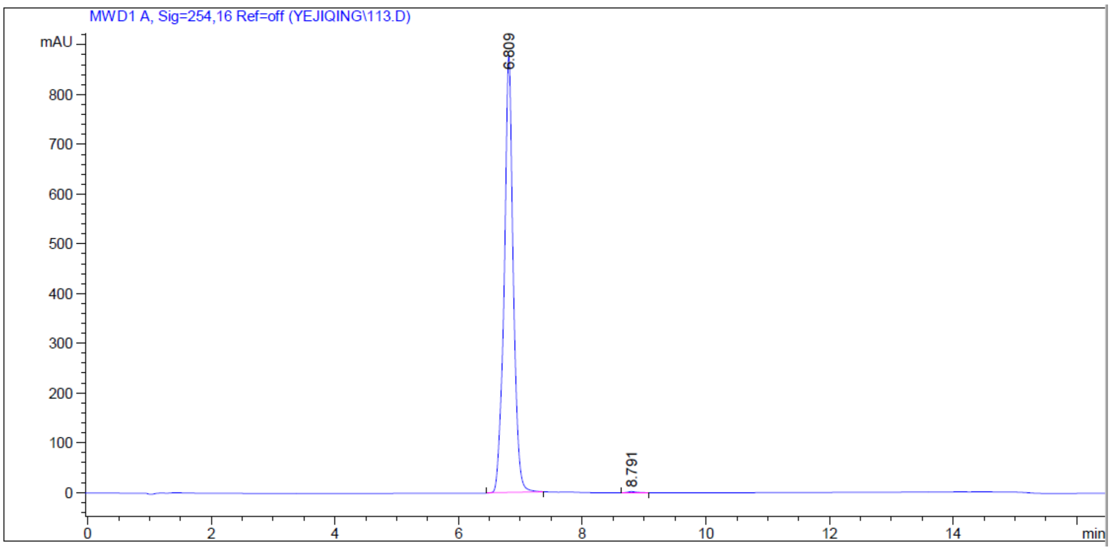

Peak #	RetTime [min]	Type	Width [min]	Area [mAU*s]	Height [mAU]	Area %
1	6.809	BB	0.1452	8978.95117	879.08911	99.7366
2	8.791	BB	0.1543	23.71176	2.22489	0.2634
Totals :				9002.66293	881.31400	

38

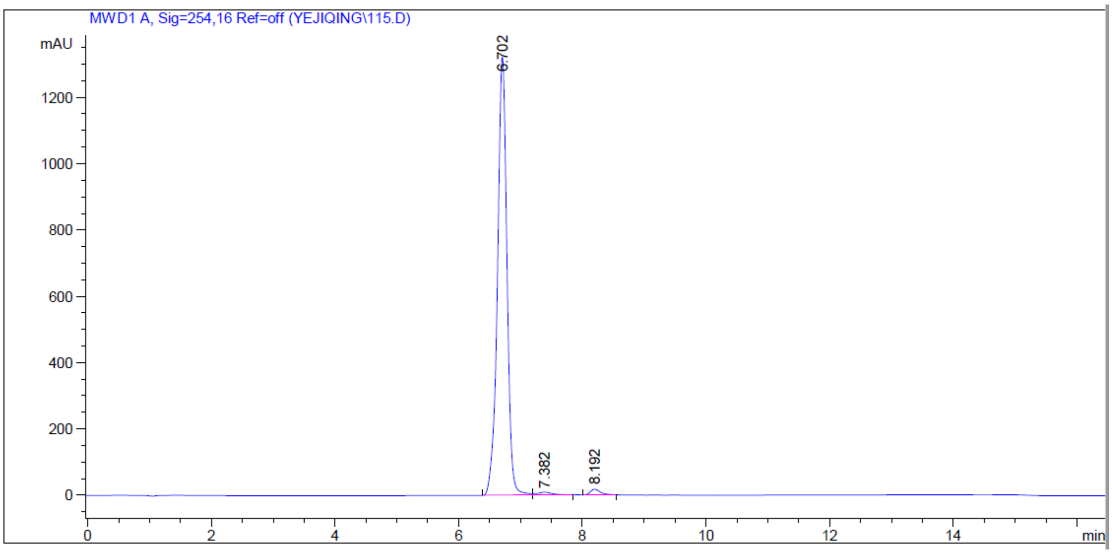

Peak #	RetTime [min]	Type	Width [min]	Area [mAU*s]	Height [mAU]	Area %
1	6.702	BB	0.1623	1.40891e4	1322.28894	97.6509
2	7.382	BB	0.2392	142.47156	8.51617	0.9875
3	8.192	BB	0.1737	196.46486	17.14346	1.3617

Totals : 1.44281e4 1347.94858

39

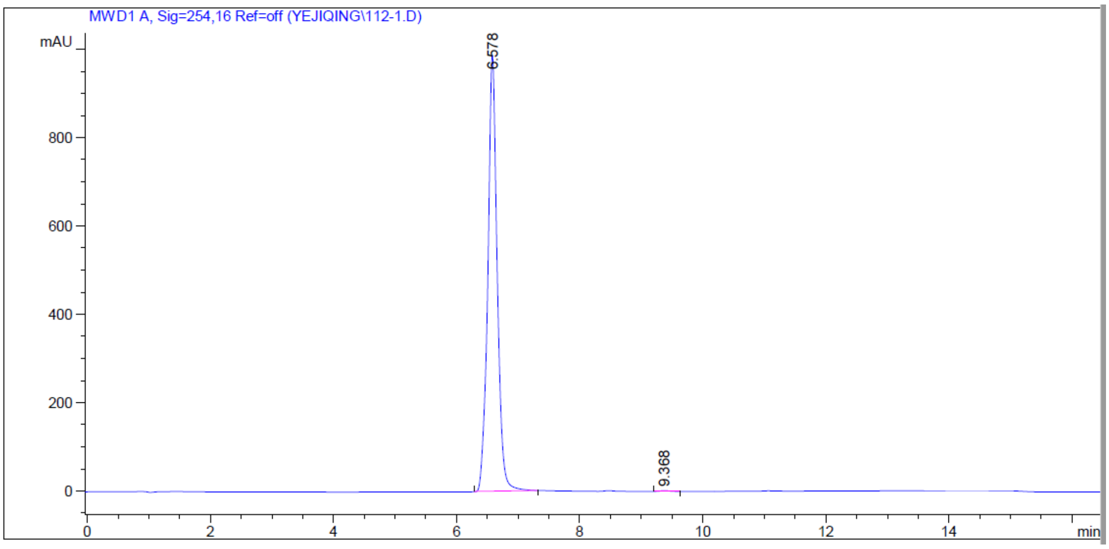

Peak #	RetTime [min]	Type	Width [min]	Area [mAU*s]	Height [mAU]	Area %
1	6.578	BB	0.1513	1.04503e4	989.00201	99.8433
2	9.368	BB	0.1480	16.40651	1.74074	0.1567

Totals : 1.04667e4 990.74276

40

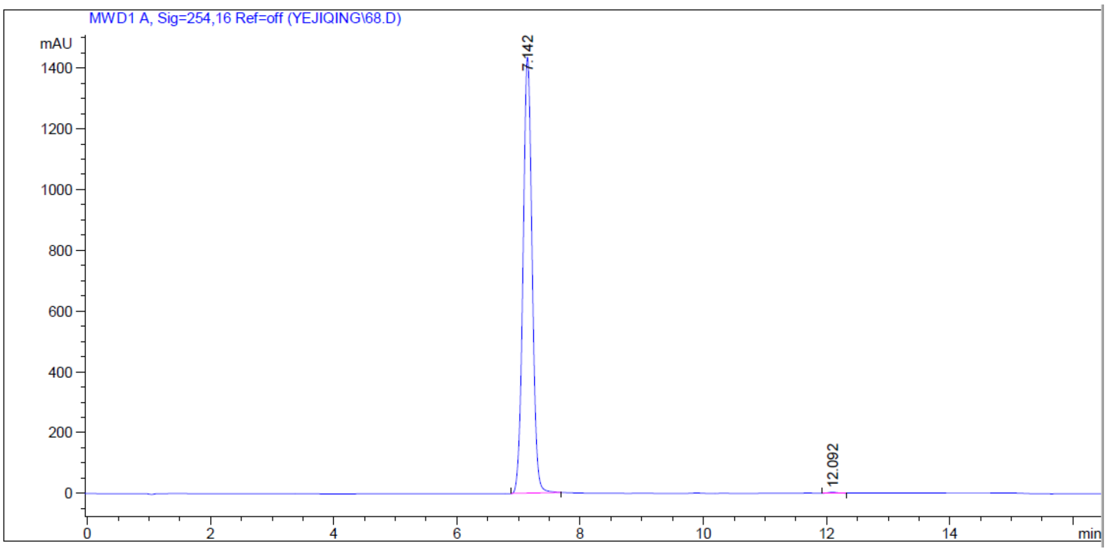

Peak #	RetTime [min]	Type	Width [min]	Area [mAU*s]	Height [mAU]	Area %
1	7.142	BB	0.1599	1.47633e4	1436.92493	99.8266
2	12.092	BB	0.1249	25.64073	3.15285	0.1734

Totals : 1.47890e4 1440.07777

41

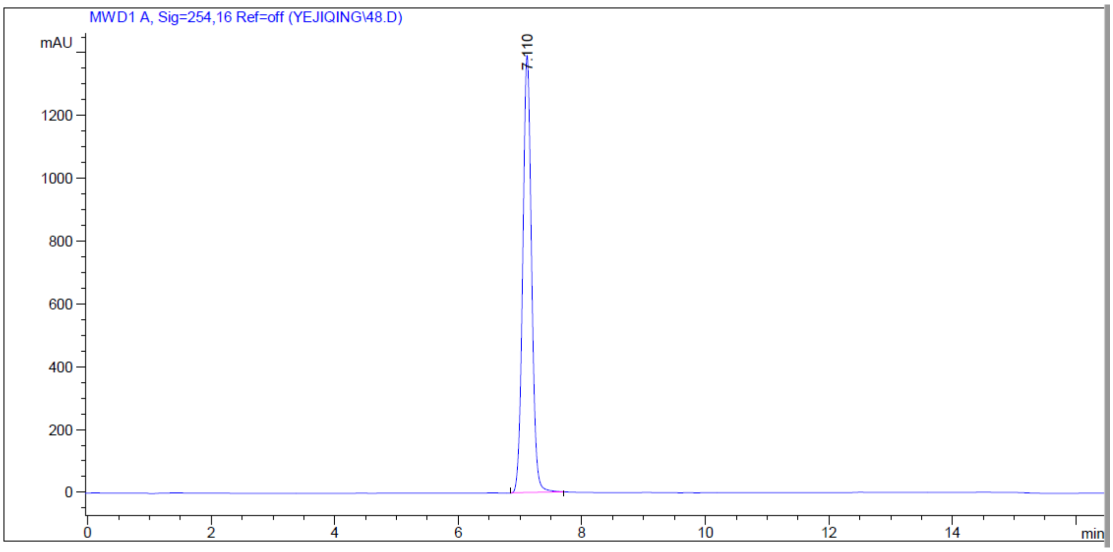

Peak #	RetTime [min]	Type	Width [min]	Area [mAU*s]	Height [mAU]	Area %
1	7.110	BB	0.1517	1.38237e4	1393.82007	100.0000

Totals : 1.38237e4 1393.82007

42

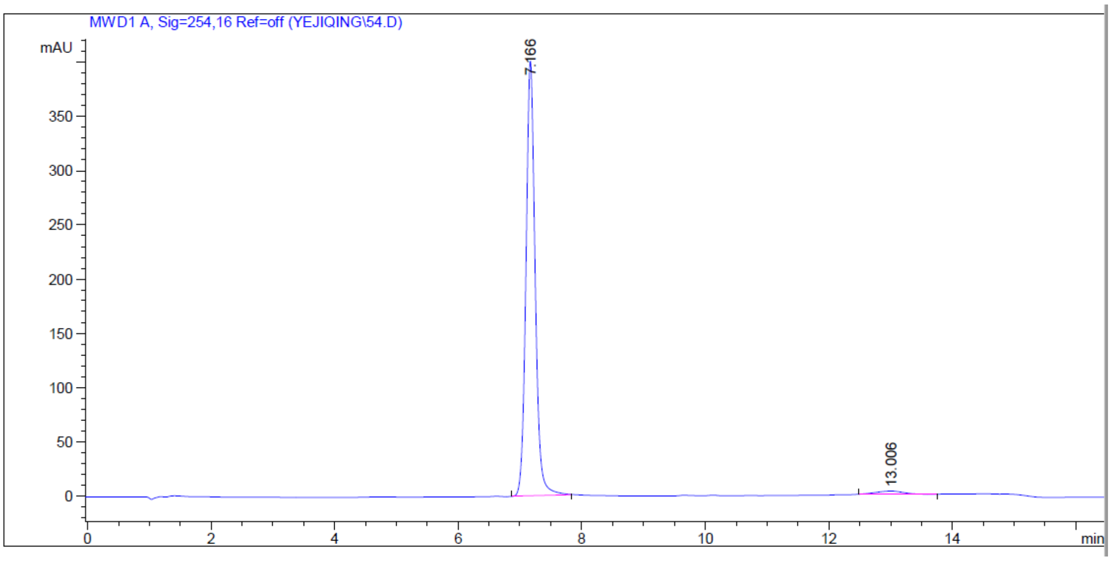

Peak #	RetTime [min]	Type	Width [min]	Area [mAU*s]	Height [mAU]	Area %
1	7.166	BB	0.1545	4143.91016	401.04831	97.9546
2	13.006	BB	0.4380	86.52998	2.91793	2.0454

Totals : 4230.44014 403.96623

43

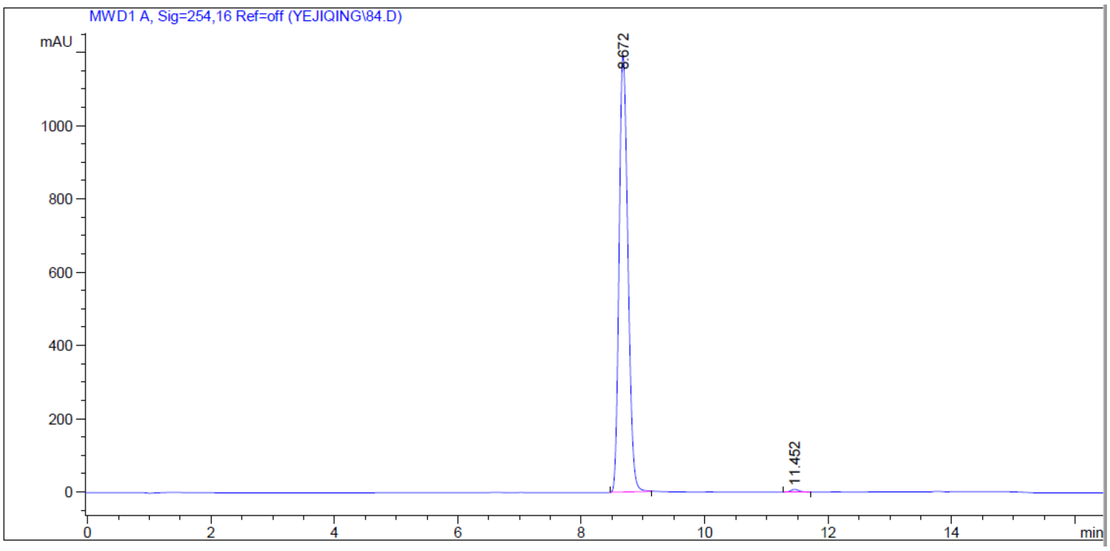

Peak #	RetTime [min]	Type	Width [min]	Area [mAU*s]	Height [mAU]	Area %
1	8.672	BB	0.1537	1.18332e4	1193.57751	99.4325
2	11.452	BB	0.1423	67.53574	7.41118	0.5675

Totals : 1.19007e4 1200.98869

44

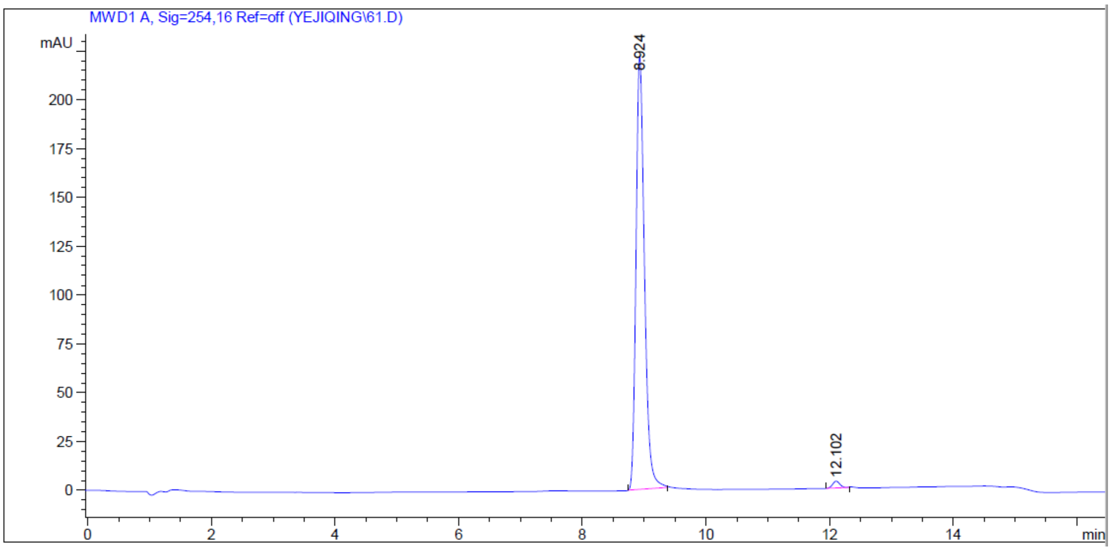

Peak #	RetTime [min]	Type	Width [min]	Area [mAU*s]	Height [mAU]	Area %
1	8.924	BB	0.1445	2104.25537	222.17053	98.6179
2	12.102	BB	0.1274	29.48985	3.53336	1.3821

Totals : 2133.74522 225.70389

45

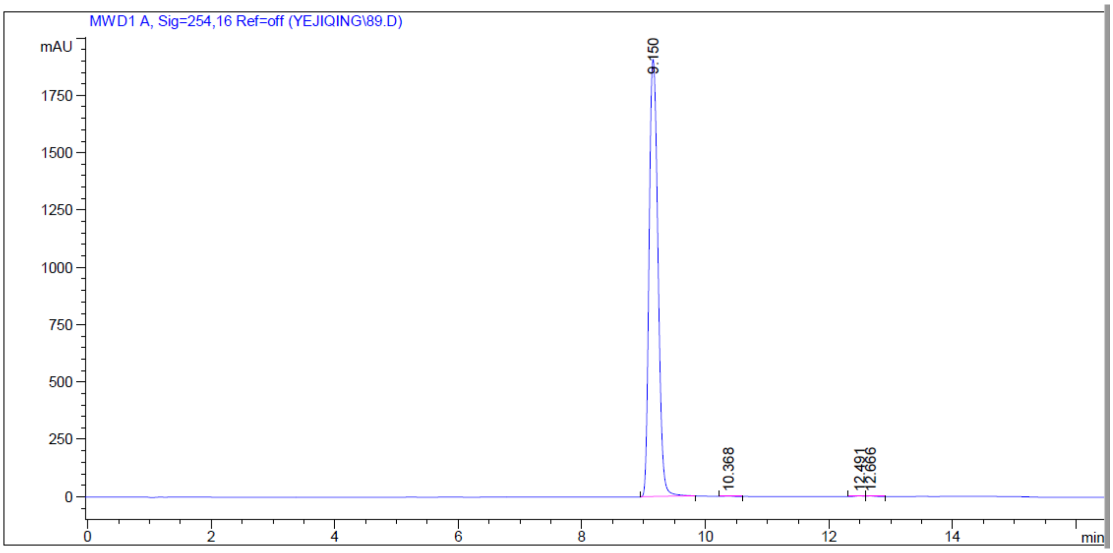

Peak #	RetTime [min]	Type	Width [min]	Area [mAU*s]	Height [mAU]	Area %
1	9.150	BB	0.1584	1.90441e4	1908.38171	99.6320
2	10.368	BB	0.1120	14.44765	2.16141	0.0756

3	12.491	BV	0.1294	30.64834	3.52525	0.1603
4	12.666	VB	0.1246	25.24574	2.98787	0.1321

Totals : 1.91145e4 1917.05625

46

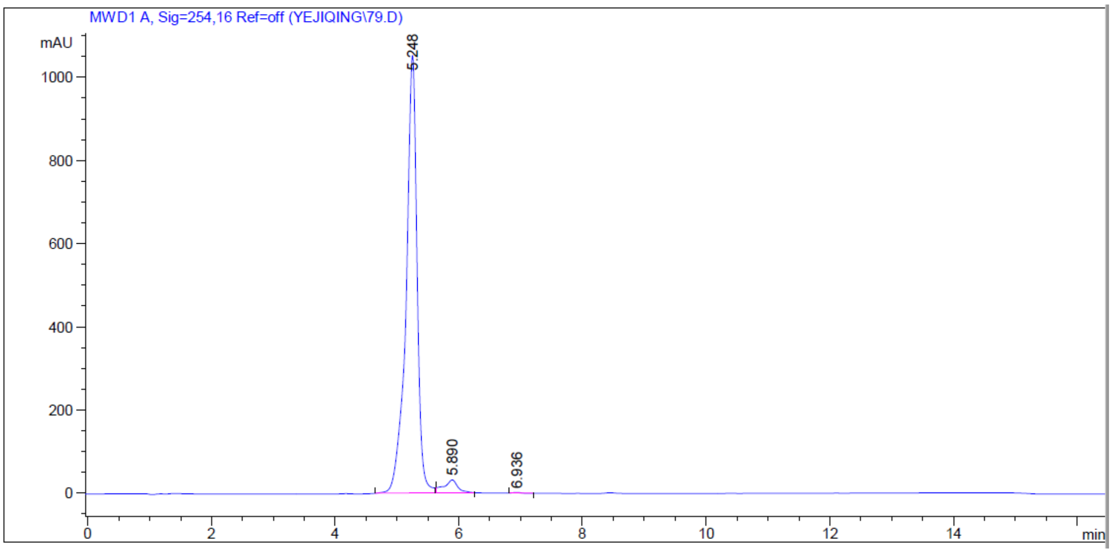

Peak #	RetTime [min]	Type	Width [min]	Area [mAU*s]	Height [mAU]	Area %
1	5.248	BB	0.1838	1.29937e4	1053.86169	96.1857
2	5.890	BB	0.2210	501.25571	31.61816	3.7105
3	6.936	BB	0.1352	14.01548	1.71740	0.1037

Totals : 1.35090e4 1087.19725

47

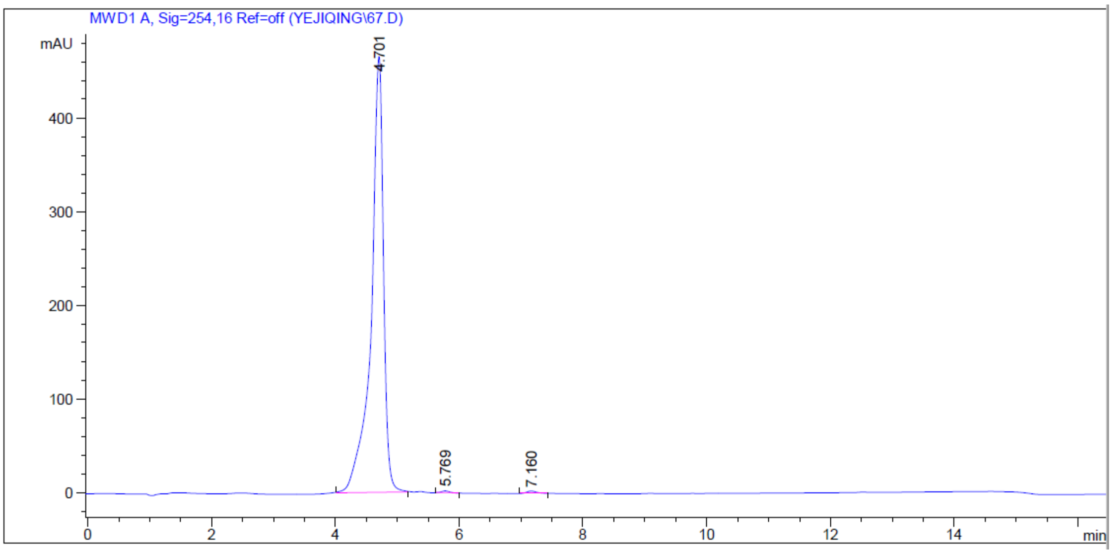

Peak #	RetTime [min]	Type	Width [min]	Area [mAU*s]	Height [mAU]	Area %
1	4.701	BB	0.2031	6428.05762	465.57159	99.2190
2	5.769	BB	0.1392	20.00532	2.13769	0.3088
3	7.160	BB	0.1711	30.59017	2.64084	0.4722

Totals : 6478.65310 470.35013

48

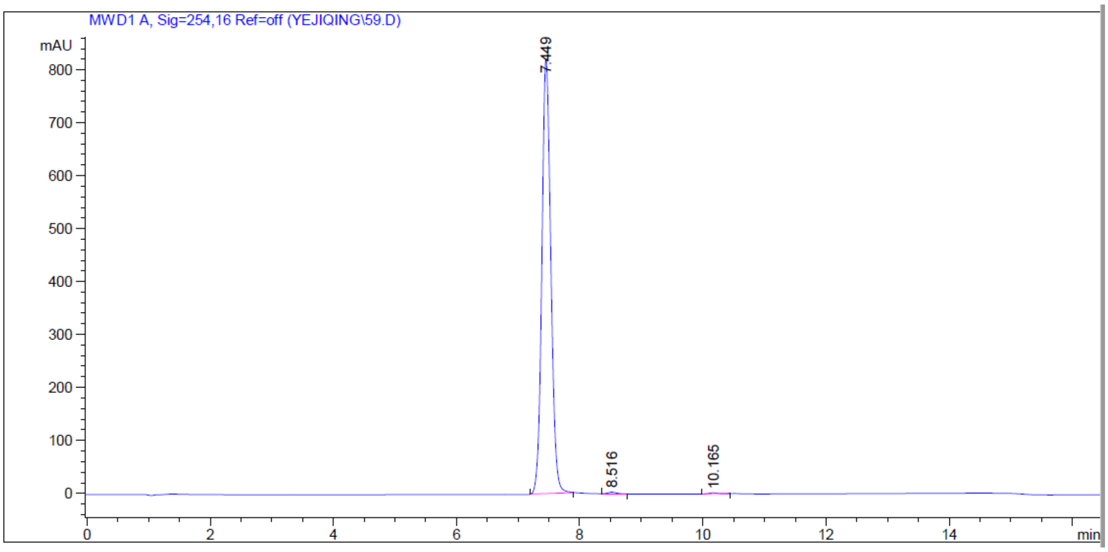

Peak #	RetTime [min]	Type	Width [min]	Area [mAU*s]	Height [mAU]	Area %
1	7.449	BB	0.1580	8312.45215	821.72119	99.4571
2	8.516	BB	0.1520	30.91981	3.16482	0.3700
3	10.165	BB	0.1303	14.45663	1.90909	0.1730

Totals : 8357.82859 826.79510

49

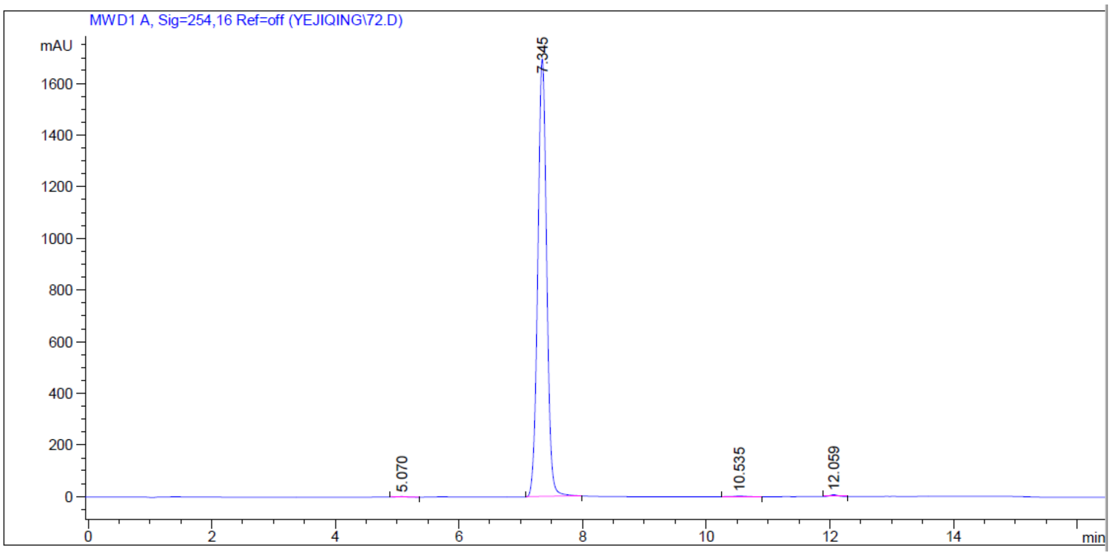

Peak #	RetTime [min]	Type	Width [min]	Area [mAU*s]	Height [mAU]	Area %
1	5.070	BB	0.1519	16.51640	1.75273	0.0964
2	7.345	BB	0.1529	1.70105e4	1697.87048	99.2901
3	10.535	BB	0.1964	41.89503	2.93818	0.2445
4	12.059	BB	0.1312	63.20271	7.28814	0.3689

Totals : 1.71321e4 1709.84954

50

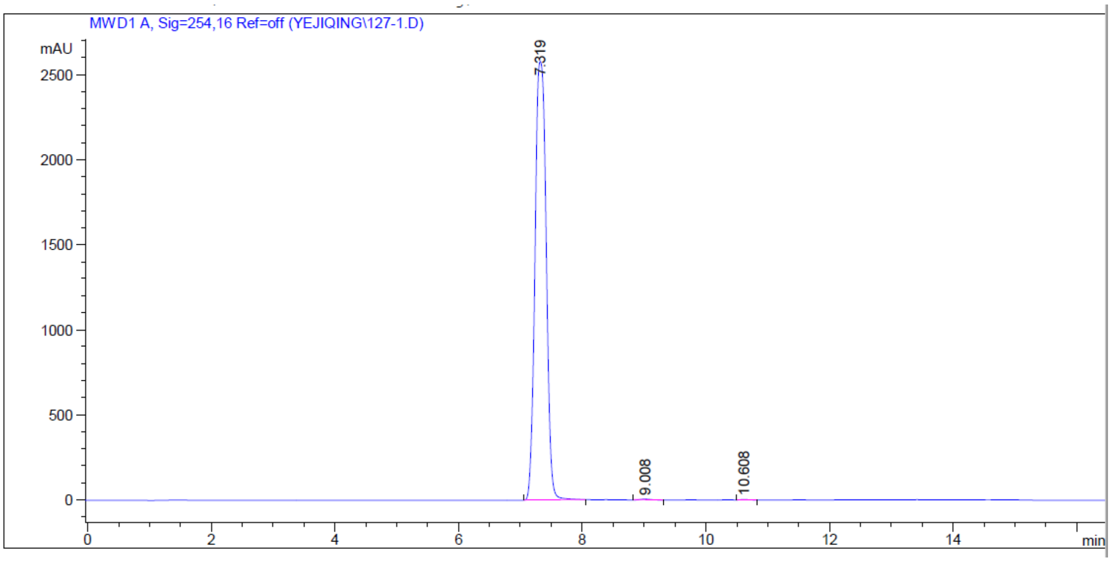

Peak #	RetTime [min]	Type	Width [min]	Area [mAU*s]	Height [mAU]	Area %
1	7.319	BB	0.2004	3.23796e4	2580.29761	99.7445
2	9.008	BB	0.1660	57.06814	5.28308	0.1758
3	10.608	VB	0.1325	25.87203	3.00289	0.0797

Totals : 3.24625e4 2588.58358

51

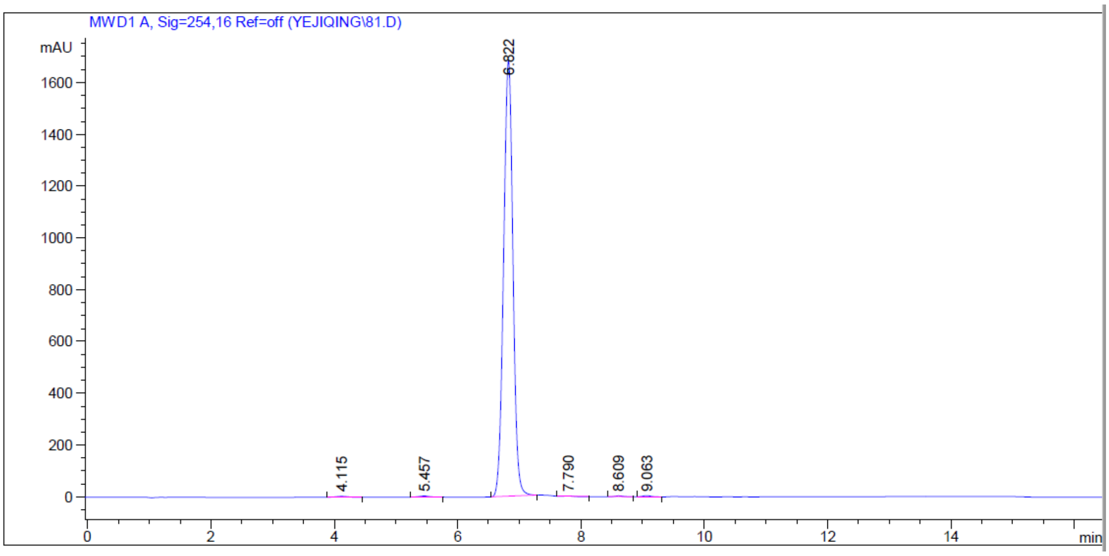

Peak #	RetTime [min]	Type	Width [min]	Area [mAU*s]	Height [mAU]	Area %
1	4.115	BB	0.2044	44.41965	3.40144	0.2479
2	5.457	BB	0.1890	53.85991	4.33391	0.3006
3	6.822	BB	0.1668	1.77253e4	1683.84851	98.9292
4	7.790	BB	0.0973	8.60564	1.75168	0.0480
5	8.609	BB	0.1541	39.09163	3.79898	0.2182
6	9.063	BB	0.1453	45.88128	4.80968	0.2561

Totals : 1.79171e4 1701.94421

52

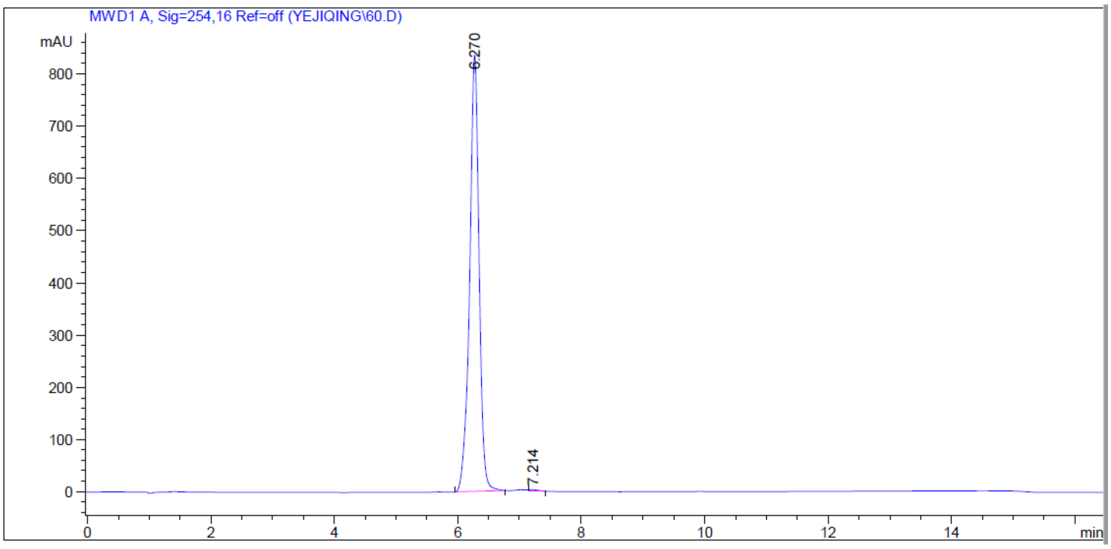

Peak #	RetTime [min]	Type	Width [min]	Area [mAU*s]	Height [mAU]	Area %
1	6.270	BB	0.1601	8893.83594	835.99084	99.7001
2	7.214	BB	0.1434	26.75282	2.80158	0.2999

Totals : 8920.58876 838.79242

53

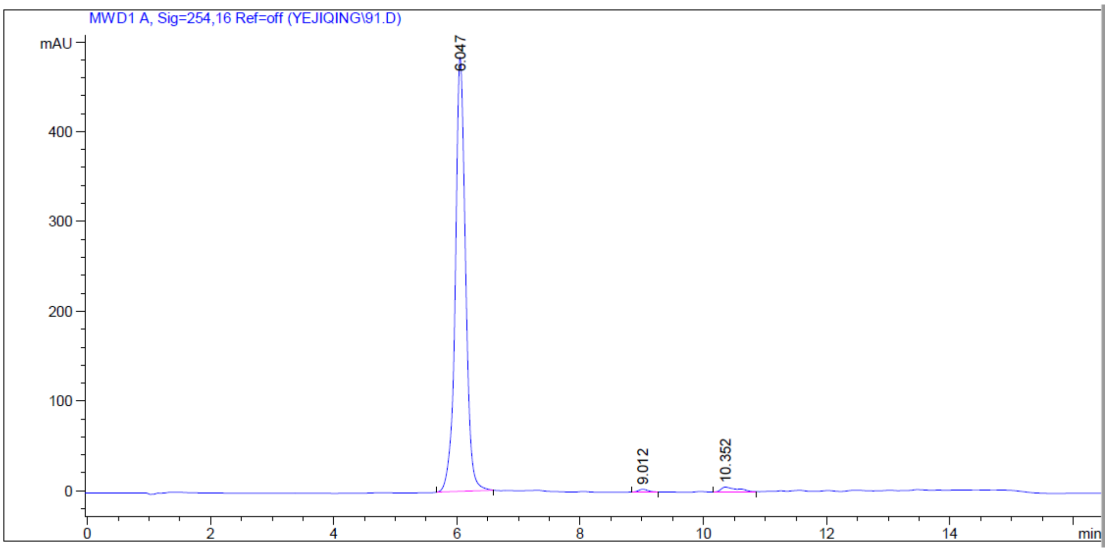

Peak #	RetTime [min]	Type	Width [min]	Area [mAU*s]	Height [mAU]	Area %
1	6.047	BB	0.1770	5602.23096	484.28204	97.6463
2	9.012	BB	0.1618	33.66855	3.17270	0.5868
3	10.352	BB	0.2509	101.36668	5.45237	1.7668

Totals : 5737.26618 492.90711

54

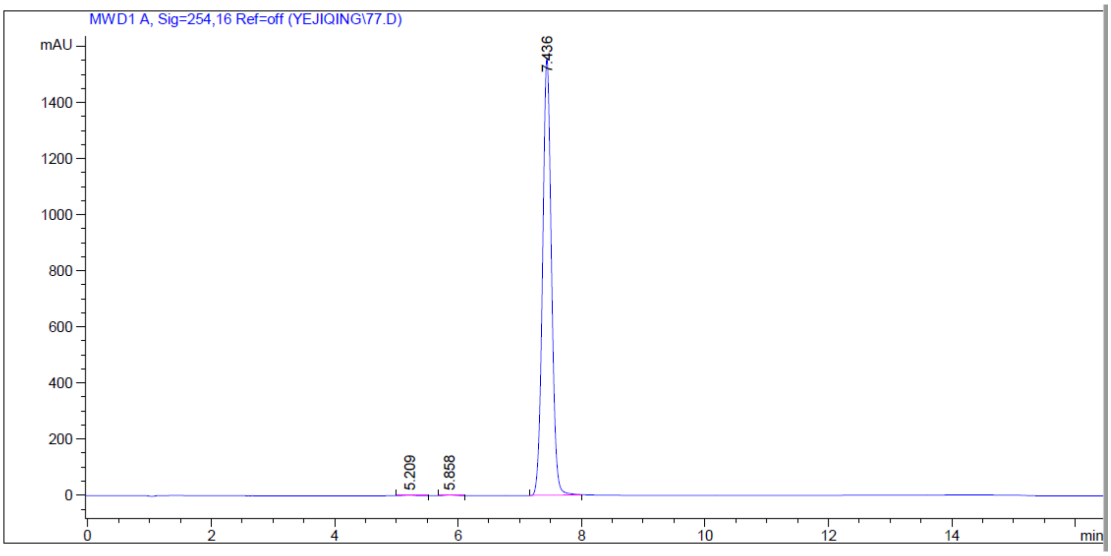

Peak #	RetTime [min]	Type	Width [min]	Area [mAU*s]	Height [mAU]	Area %
1	5.209	BB	0.1785	24.80259	2.18323	0.1592
2	5.858	BB	0.1617	33.07846	2.97444	0.2124
3	7.436	BB	0.1522	1.55190e4	1557.57117	99.6284

Totals : 1.55769e4 1562.72884

55

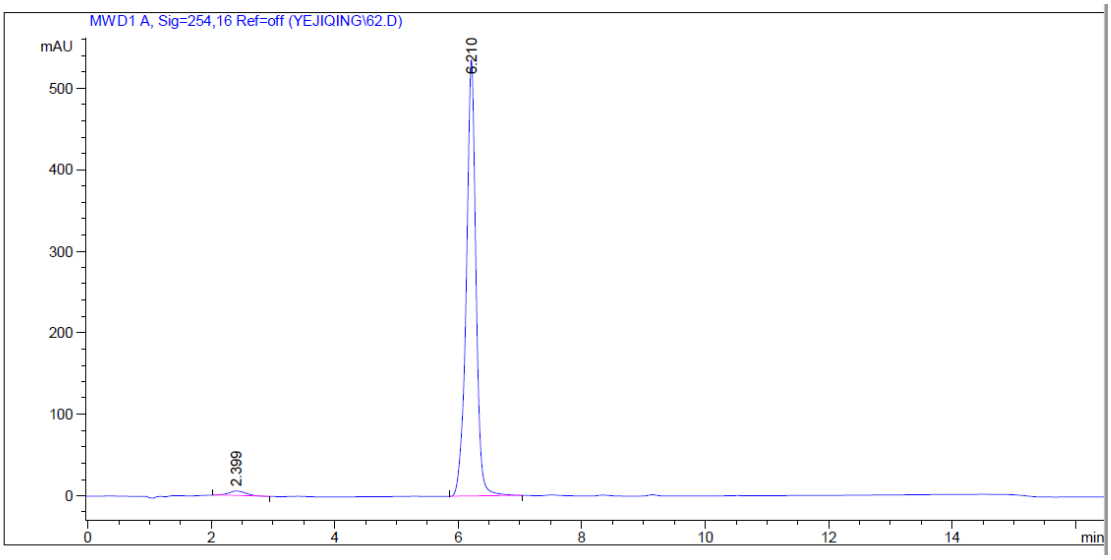

Peak #	RetTime [min]	Type	Width [min]	Area [mAU*s]	Height [mAU]	Area %
1	2.399	BB	0.3126	117.61034	5.75177	1.9759
2	6.210	BB	0.1610	5834.59570	535.92047	98.0241

Totals : 5952.20604 541.67224

56

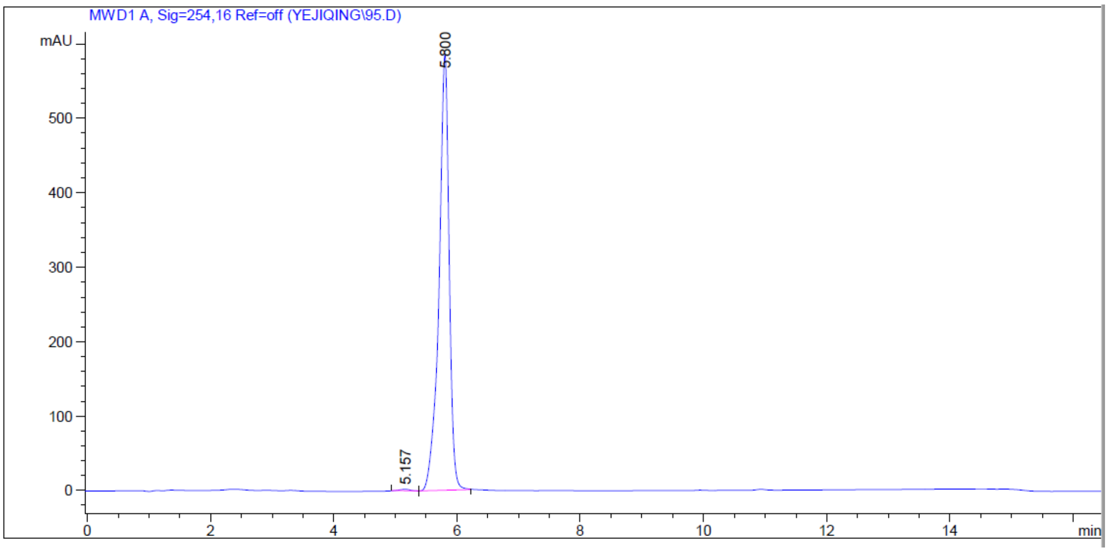

Peak #	RetTime [min]	Type	Width [min]	Area [mAU*s]	Height [mAU]	Area %
1	5.157	BV	0.1757	25.45130	2.18790	0.3769
2	5.800	VB	0.1719	6727.01904	586.28418	99.6231

Totals : 6752.47034 588.47208

57

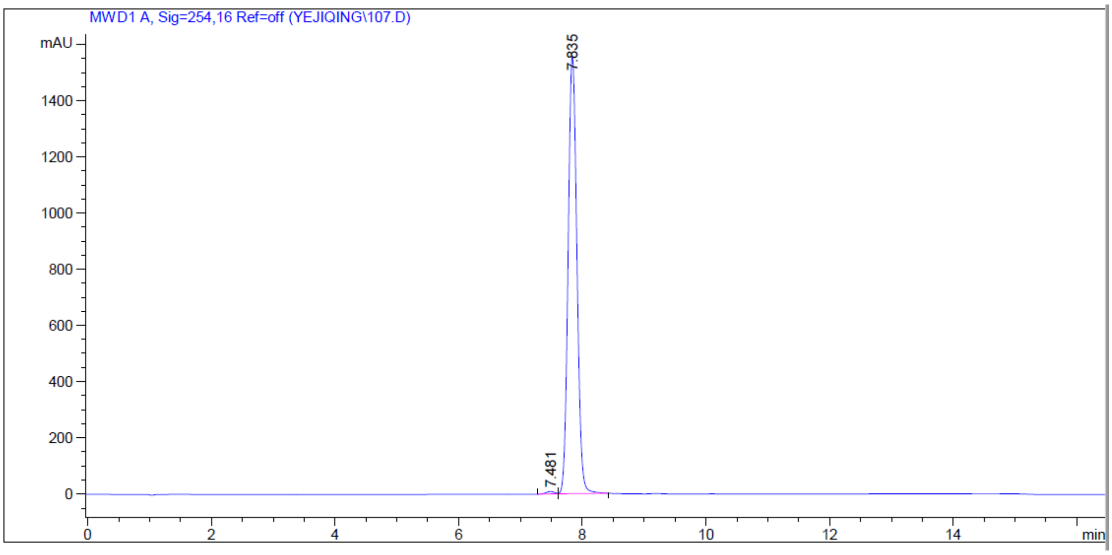

Peak #	RetTime [min]	Type	Width [min]	Area [mAU*s]	Height [mAU]	Area %
1	7.481	BV	0.1515	90.38239	9.12960	0.5842
2	7.835	VB	0.1552	1.53802e4	1556.92700	99.4158

Totals : 1.54706e4 1566.05660

58

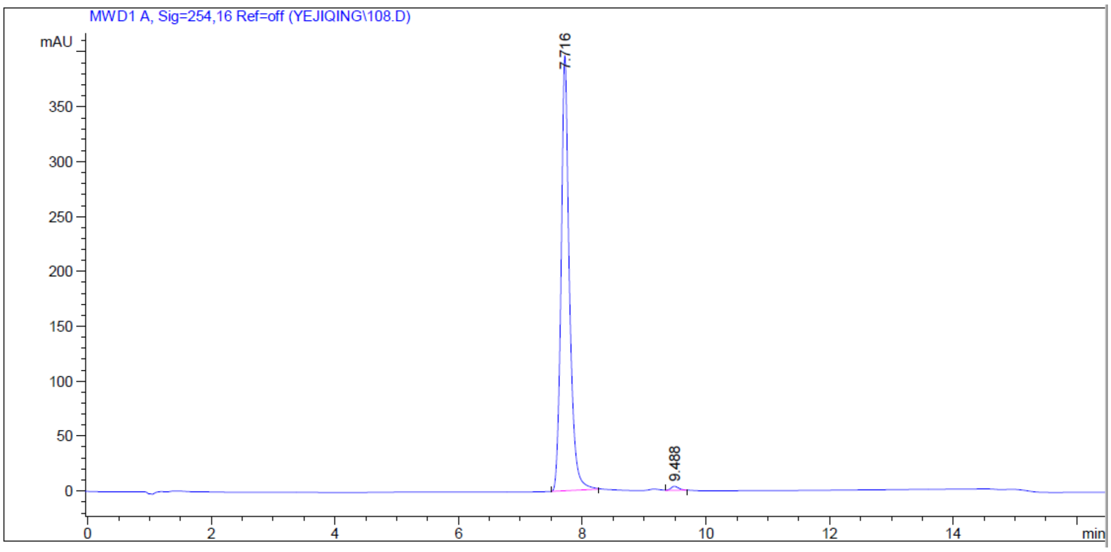

Peak #	RetTime [min]	Type	Width [min]	Area [mAU*s]	Height [mAU]	Area %
1	7.716	BB	0.1351	3713.08032	397.10507	99.0293
2	9.488	BB	0.1412	36.39439	3.81764	0.9707

Totals : 3749.47471 400.92271

59

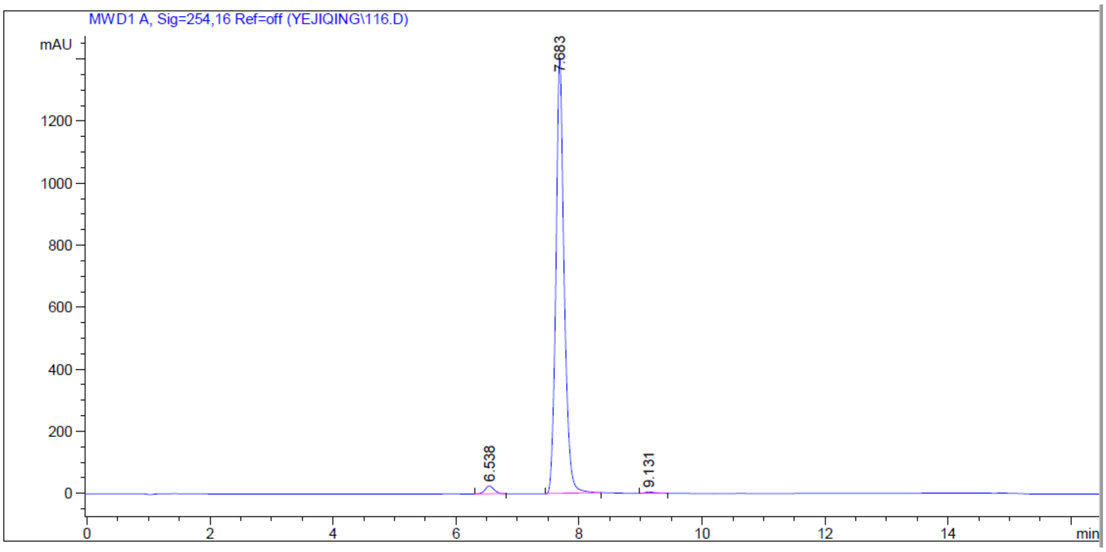

Peak #	RetTime [min]	Type	Width [min]	Area [mAU*s]	Height [mAU]	Area %
1	6.538	BB	0.1578	271.70786	26.02177	2.0188
2	7.683	BB	0.1350	1.31337e4	1405.22766	97.5837
3	9.131	BB	0.1633	53.49817	4.75474	0.3975

Totals : 1.34589e4 1436.00417

60

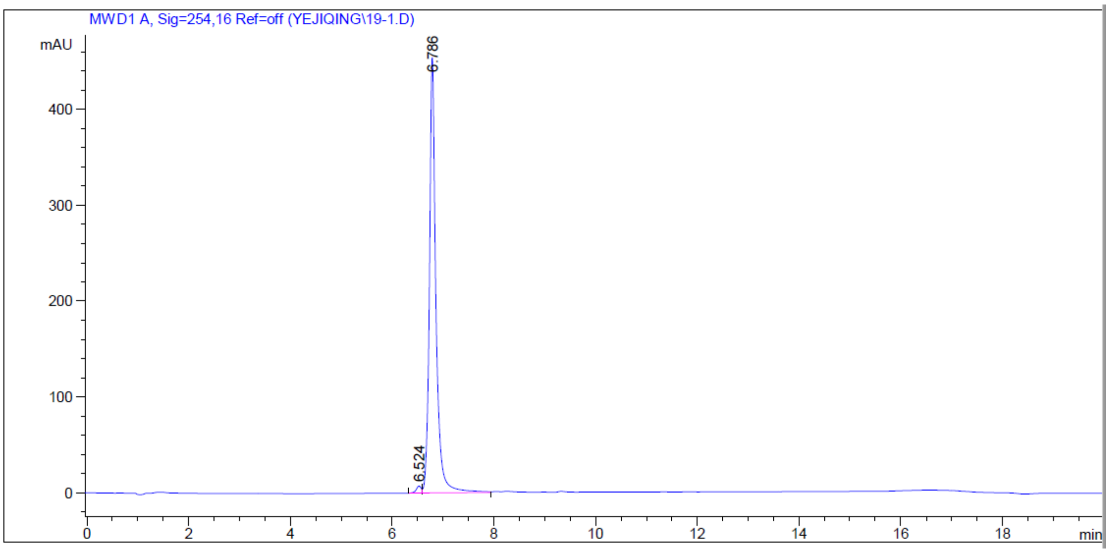

Peak #	RetTime [min]	Type	Width [min]	Area [mAU*s]	Height [mAU]	Area %
1	6.524	BV	0.1026	51.69344	7.47917	1.2523
2	6.786	VV	0.1284	4076.16113	455.40280	98.7477
Totals :				4127.85457	462.88197	

61

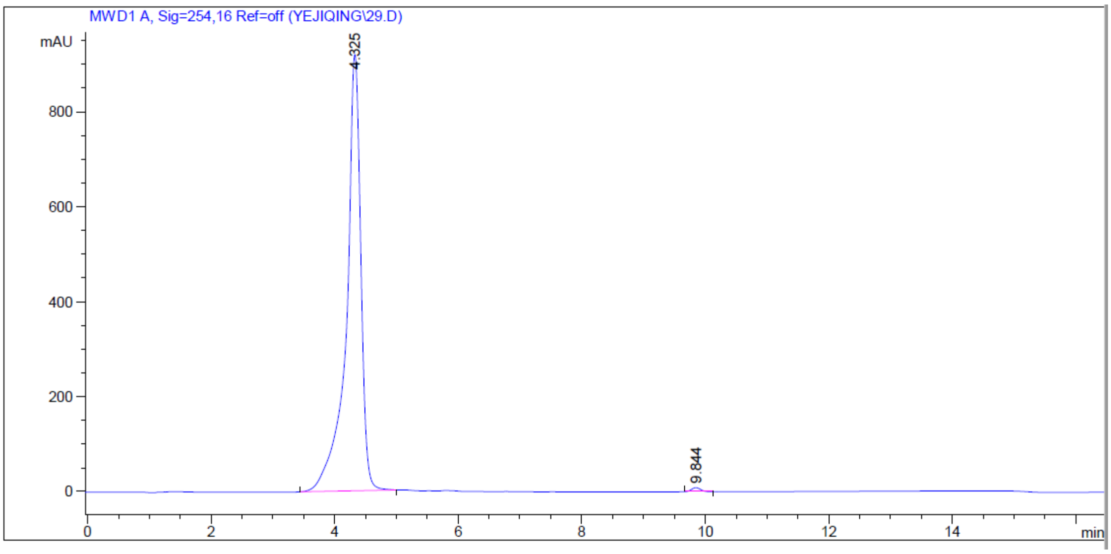

Peak #	RetTime [min]	Type	Width [min]	Area [mAU*s]	Height [mAU]	Area %
1	4.325	BB	0.2355	1.49319e4	919.51483	99.4034
2	9.844	BB	0.1625	89.61214	8.67753	0.5966
Totals :				1.50215e4	928.19236	

62

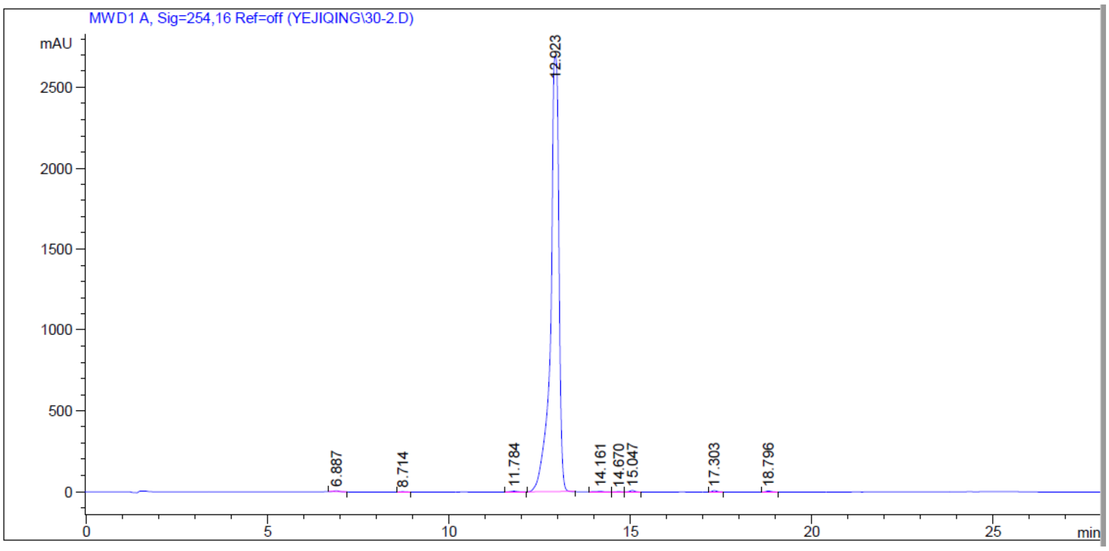

Peak #	RetTime [min]	Type	Width [min]	Area [mAU*s]	Height [mAU]	Area %
1	6.887	BB	0.1823	73.14928	6.08127	0.1682
2	8.714	BB	0.1355	19.46807	2.15311	0.0448
3	11.784	BB	0.2195	67.05471	4.61569	0.1542
4	12.923	BB	0.2363	4.29984e4	2693.03198	98.8809
5	14.161	BB	0.1969	57.73234	4.13451	0.1328
6	14.670	BV	0.1543	23.18188	2.28573	0.0533
7	15.047	VB	0.1491	93.64468	9.49510	0.2153
8	17.303	BB	0.1509	85.81383	8.71121	0.1973
9	18.796	BB	0.1568	66.60458	6.53878	0.1532

Totals : 4.34850e4 2737.04739

63

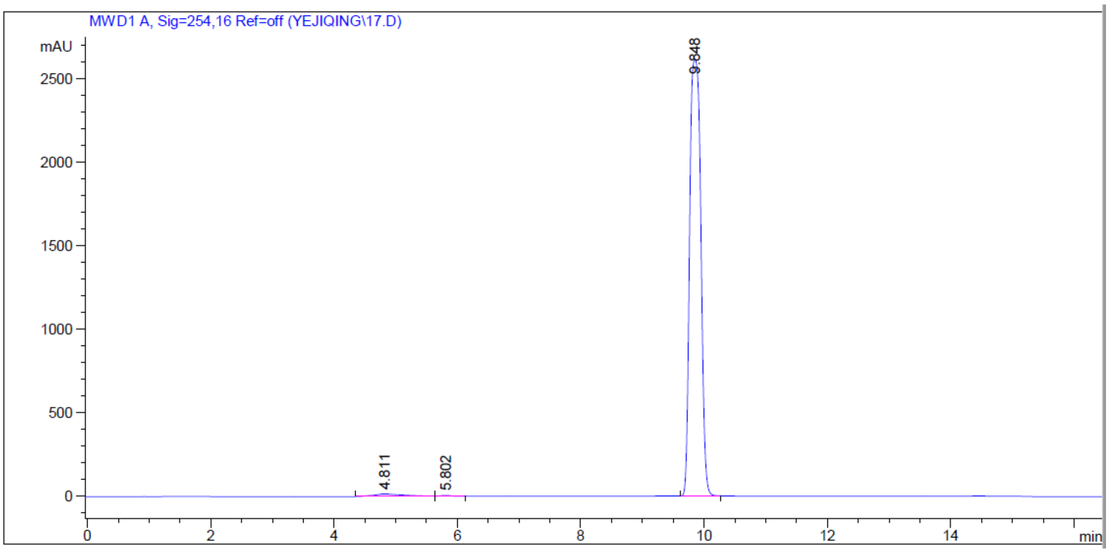

Peak #	RetTime [min]	Type	Width [min]	Area [mAU*s]	Height [mAU]	Area %
1	4.811	BB	0.4653	547.27380	15.59784	1.6508
2	5.802	BB	0.1922	75.48186	6.10666	0.2277
3	9.848	BB	0.2033	3.25293e4	2612.65112	98.1215
Totals :				3.31521e4	2634.35562	

64

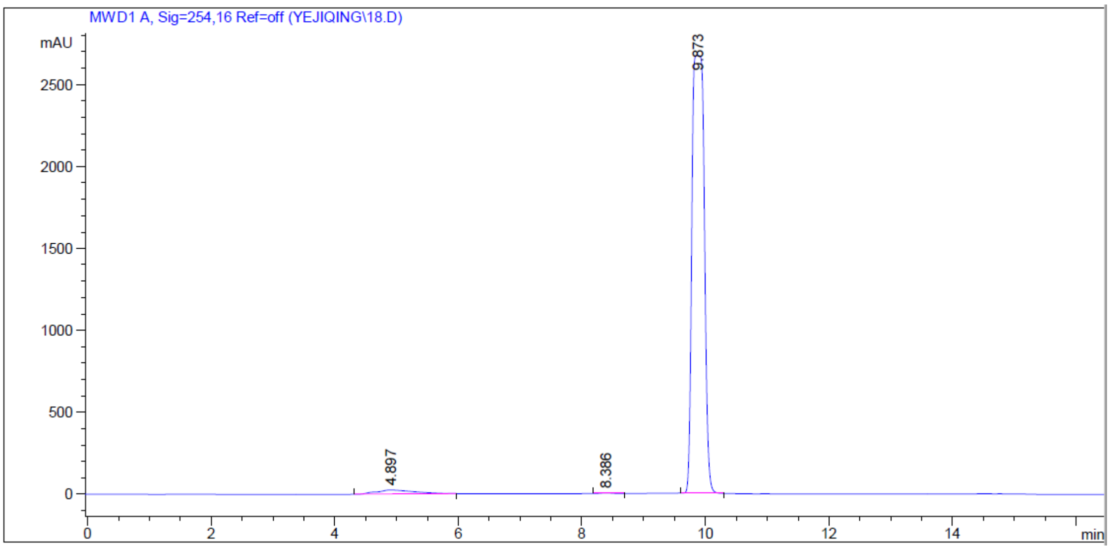

Peak #	RetTime [min]	Type	Width [min]	Area [mAU*s]	Height [mAU]	Area %
1	4.897	BB	0.6043	1070.44519	25.06307	2.8220
2	8.386	BB	0.1876	45.97951	3.73591	0.1212
3	9.873	BB	0.2228	3.68157e4	2671.64087	97.0568
Totals :				3.79321e4	2700.43984	

65

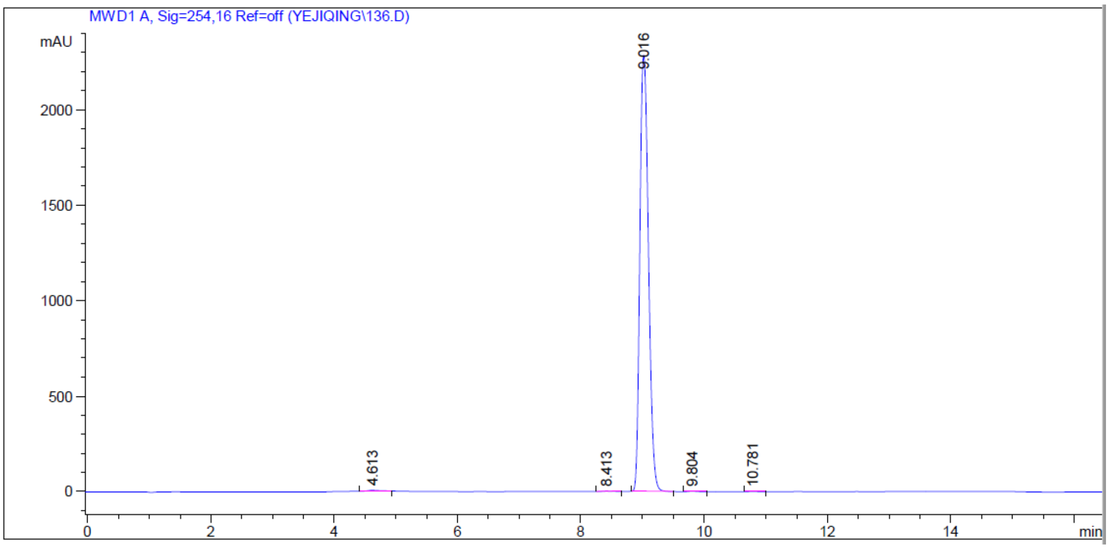

Peak #	RetTime [min]	Type	Width [min]	Area [mAU*s]	Height [mAU]	Area %
1	4.613	BB	0.2159	77.58837	4.97557	0.3493
2	8.413	BB	0.1378	19.52171	2.07415	0.0879
3	9.016	BB	0.1507	2.20780e4	2286.20093	99.3883
4	9.804	BB	0.1223	22.69685	2.80857	0.1022
5	10.781	BB	0.1157	16.08505	2.09316	0.0724

Totals : 2.22139e4 2298.15237

66

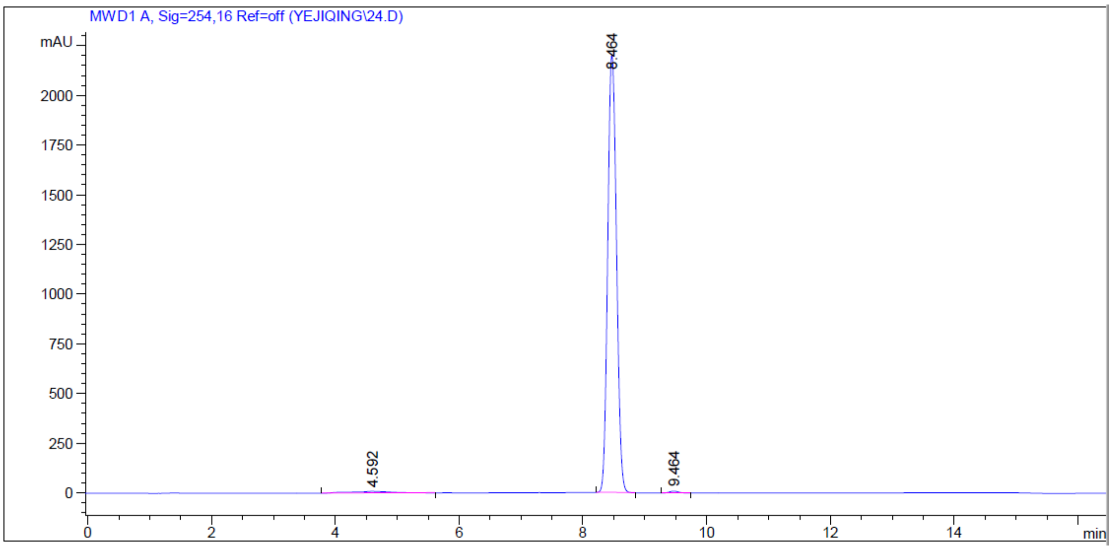

Peak #	RetTime [min]	Type	Width [min]	Area [mAU*s]	Height [mAU]	Area %
1	4.592	BB	0.5949	407.22604	8.65542	1.8357
2	8.464	BB	0.1549	2.16842e4	2202.67017	97.7467

3	9.464	BB	0.1591	92.64904	9.22978	0.4176

Totals : 2.21841e4 2220.55537

67

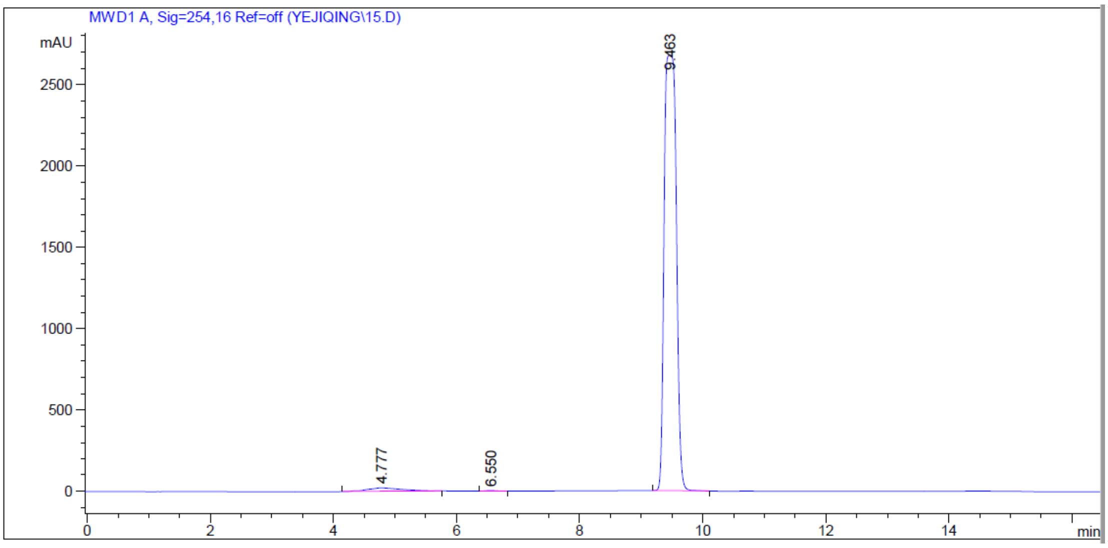

Peak #	RetTime [min]	Type	Width [min]	Area [mAU*s]	Height [mAU]	Area %
1	4.777	BB	0.5056	851.13300	21.79302	2.2741
2	6.550	BB	0.1587	31.43125	3.14342	0.0840
3	9.463	BB	0.2213	3.65441e4	2676.11792	97.6419

Totals : 3.74267e4 2701.05436

68

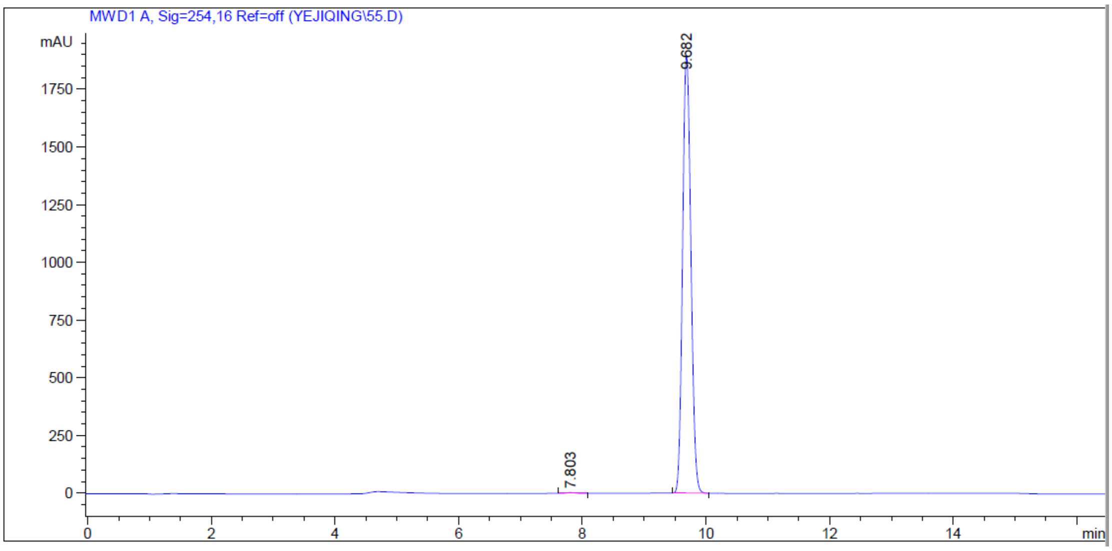

Peak #	RetTime [min]	Type	Width [min]	Area [mAU*s]	Height [mAU]	Area %
1	7.803	BB	0.1621	31.95619	3.10548	0.1800
2	9.682	BB	0.1469	1.77213e4	1897.79236	99.8200

Totals : 1.77533e4 1900.89784

69

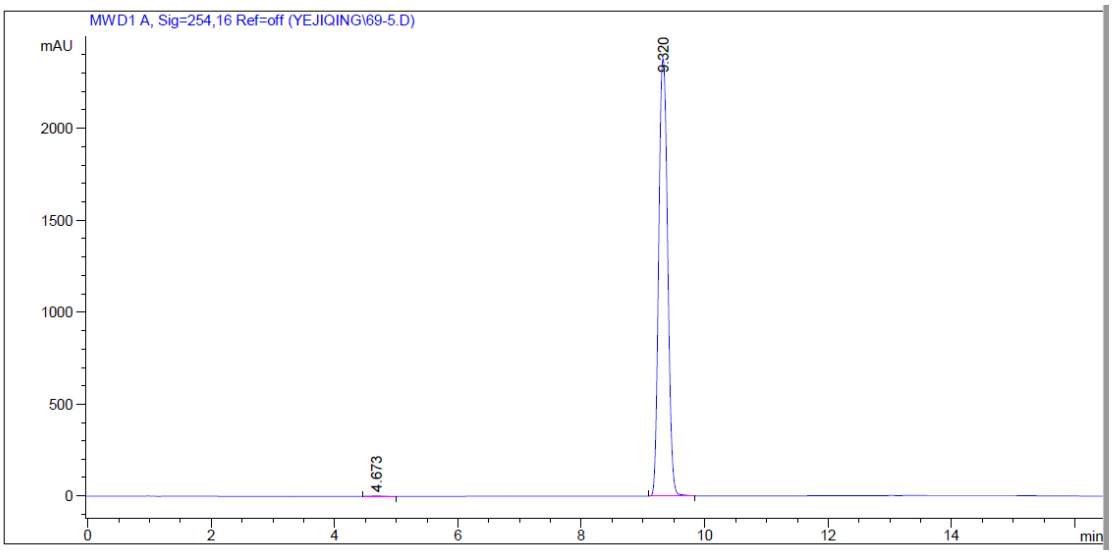

Peak #	RetTime [min]	Type	Width [min]	Area [mAU*s]	Height [mAU]	Area %
1	4.673	BB	0.1908	24.93131	2.00924	0.1041
2	9.320	BB	0.1613	2.39277e4	2379.57788	99.8959

Totals : 2.39526e4 2381.58712

70

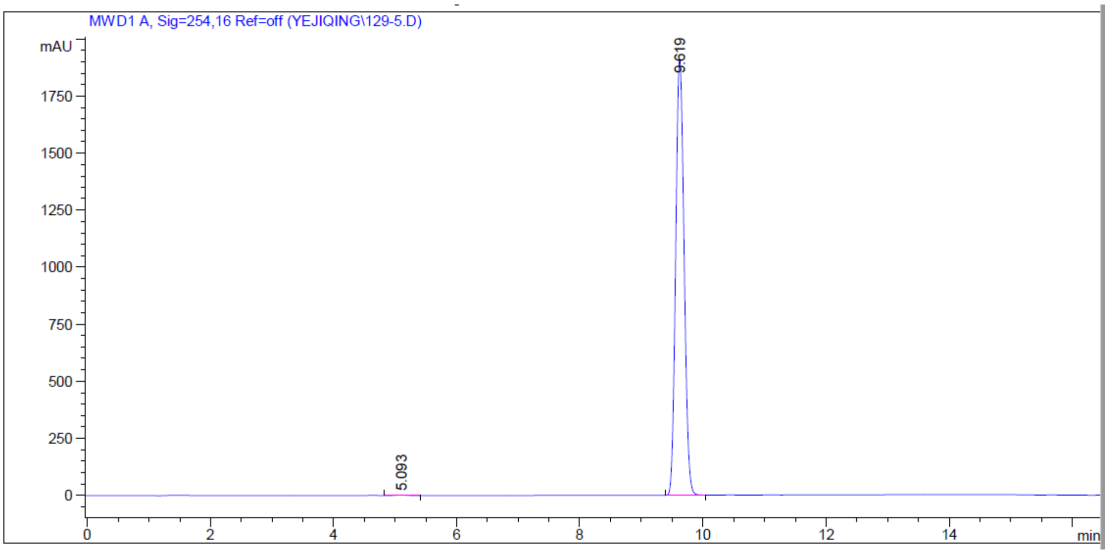

Peak #	RetTime [min]	Type	Width [min]	Area [mAU*s]	Height [mAU]	Area %
1	5.093	VB	0.1957	32.63926	2.51057	0.1796
2	9.619	BB	0.1487	1.81404e4	1911.39844	99.8204

Totals : 1.81730e4 1913.90900

71

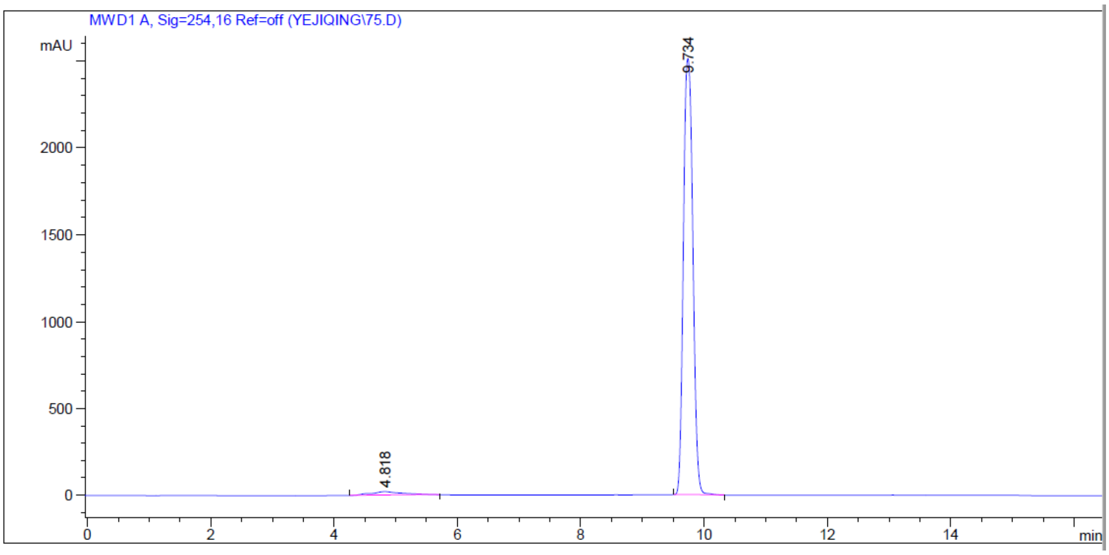

Peak #	RetTime [min]	Type	Width [min]	Area [mAU*s]	Height [mAU]	Area %
1	4.818	BB	0.4944	775.14539	20.81658	2.8127
2	9.734	BB	0.1704	2.67832e4	2512.60571	97.1873

Totals : 2.75583e4 2533.42230

72

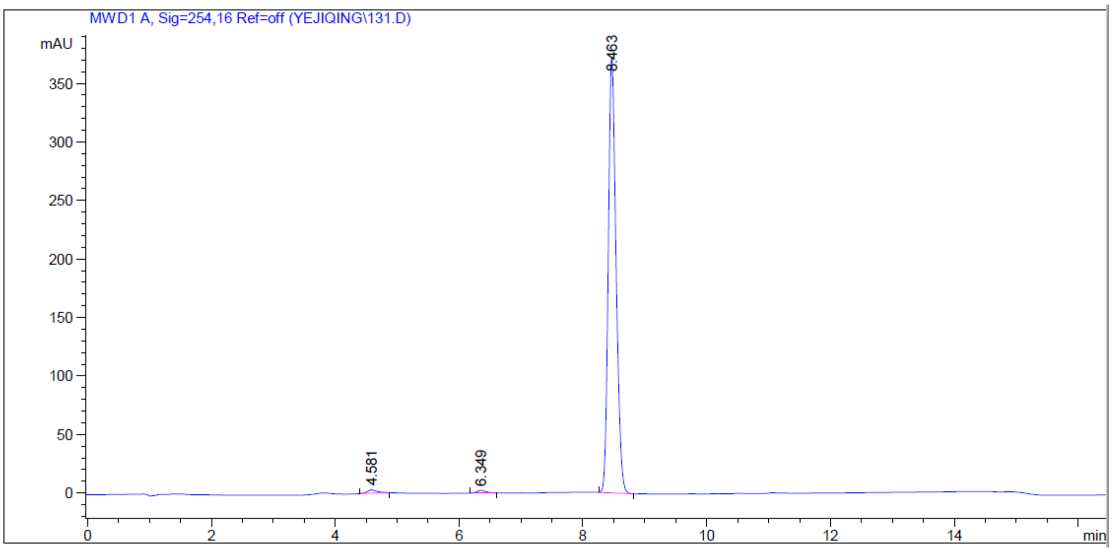

Peak #	RetTime [min]	Type	Width [min]	Area [mAU*s]	Height [mAU]	Area %
1	4.581	BB	0.1744	38.35247	3.09861	1.1583
2	6.349	BB	0.1517	26.30708	2.52150	0.7945
3	8.463	BB	0.1317	3246.31763	372.24680	98.0471
Totals :				3310.97718	377.86691	

73

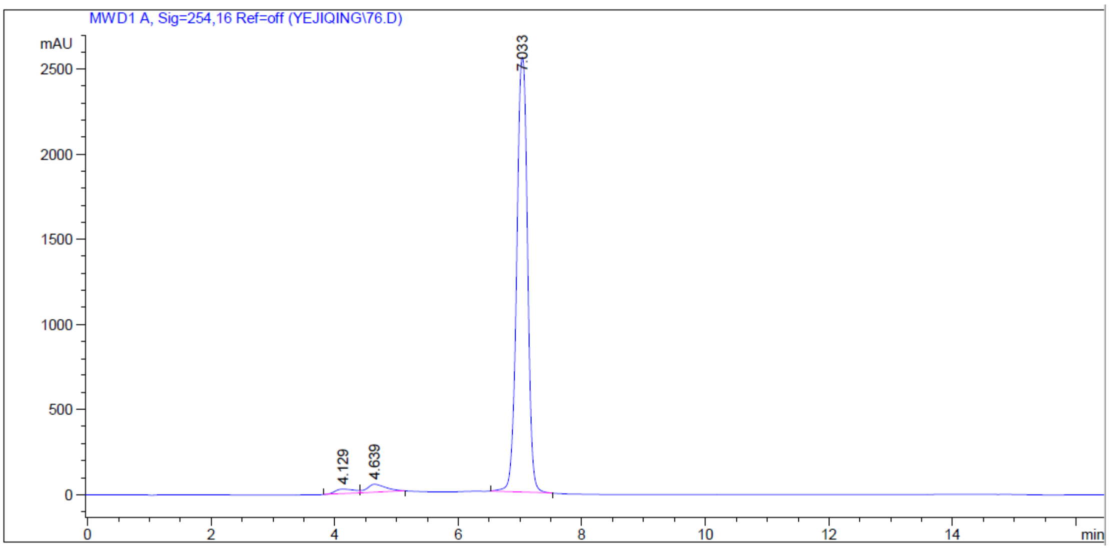

Peak #	RetTime [min]	Type	Width [min]	Area [mAU*s]	Height [mAU]	Area %
1	4.129	BV	0.3413	595.88434	27.02216	1.8217
2	4.639	VB	0.3086	1016.77313	47.72490	3.1084
3	7.033	BB	0.1919	3.10977e4	2557.24072	95.0699
Totals :				3.27104e4	2631.98779	

75

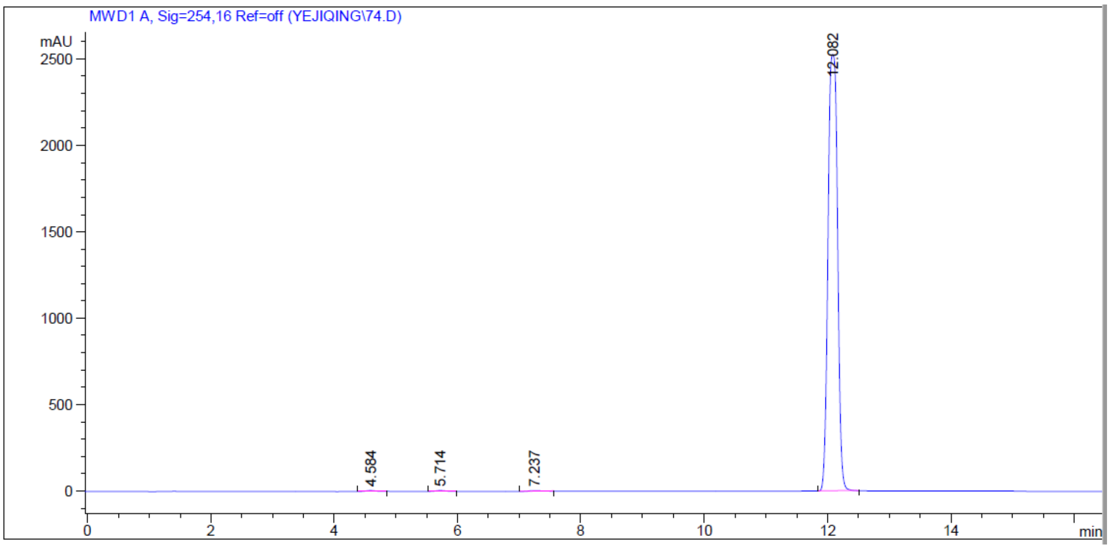

Peak #	RetTime [min]	Type	Width [min]	Area [mAU*s]	Height [mAU]	Area %
1	4.584	BB	0.1748	59.74694	5.02151	0.2194
2	5.714	BB	0.1709	55.63214	4.88497	0.2043
3	7.237	BB	0.2232	61.27763	4.03499	0.2251
4	12.082	BB	0.1710	2.70493e4	2525.23218	99.3511

Totals : 2.72259e4 2539.17364

76

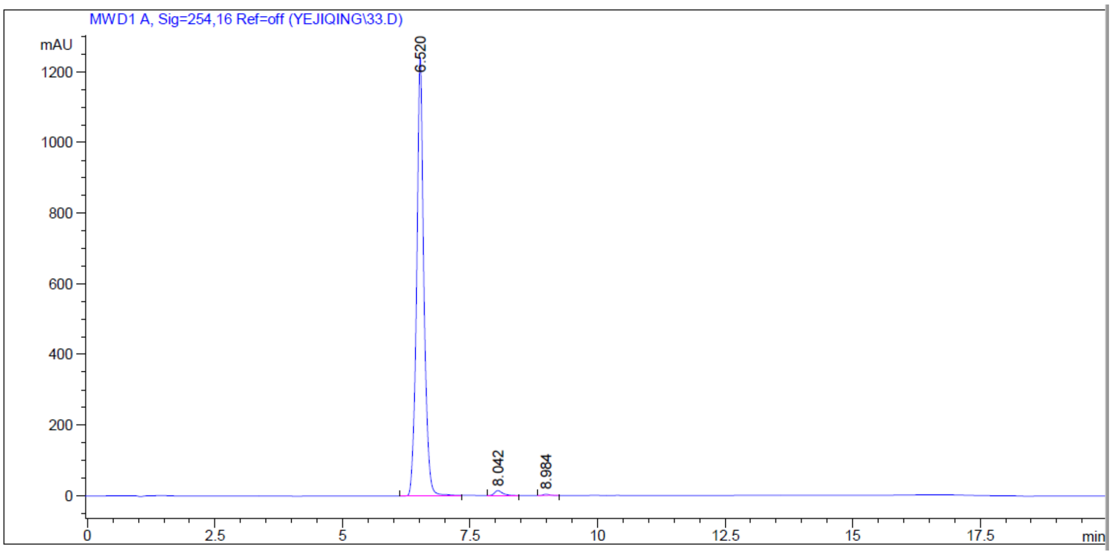

Peak #	RetTime [min]	Type	Width [min]	Area [mAU*s]	Height [mAU]	Area %
1	6.520	BV	0.1410	1.22513e4	1243.42651	98.2732
2	8.042	VV	0.1696	177.64162	14.84649	1.4249
3	8.984	VB	0.1365	37.63499	4.04836	0.3019

Totals : 1.24666e4 1262.32137

77

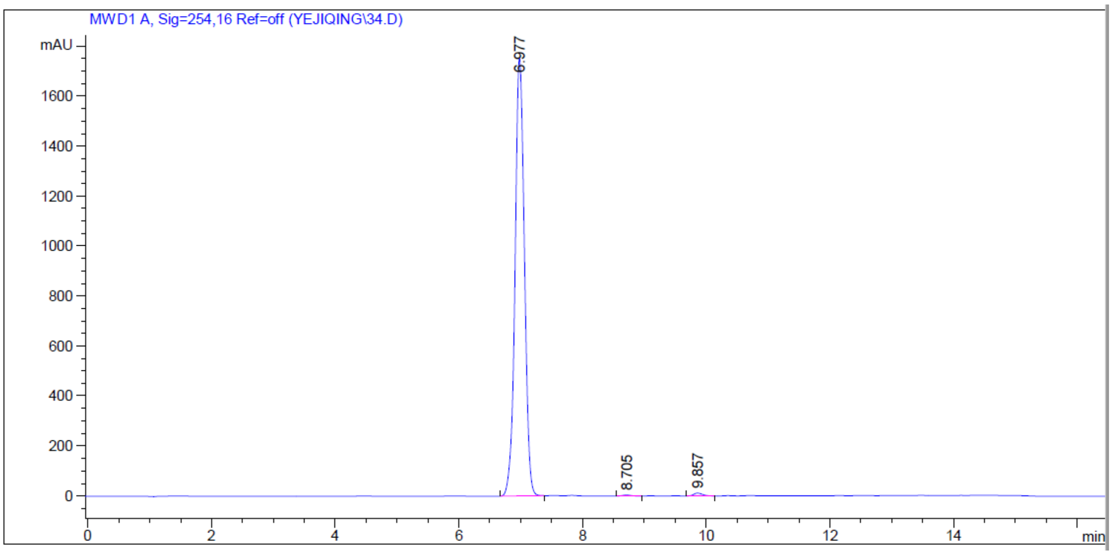

Peak #	RetTime [min]	Type	Width [min]	Area [mAU*s]	Height [mAU]	Area %
1	6.977	BB	0.1646	1.84365e4	1754.66089	99.2001
2	8.705	BB	0.1432	35.72697	3.96239	0.1922
3	9.857	BB	0.1510	112.93526	11.45704	0.6077

Totals : 1.85851e4 1770.08032

78

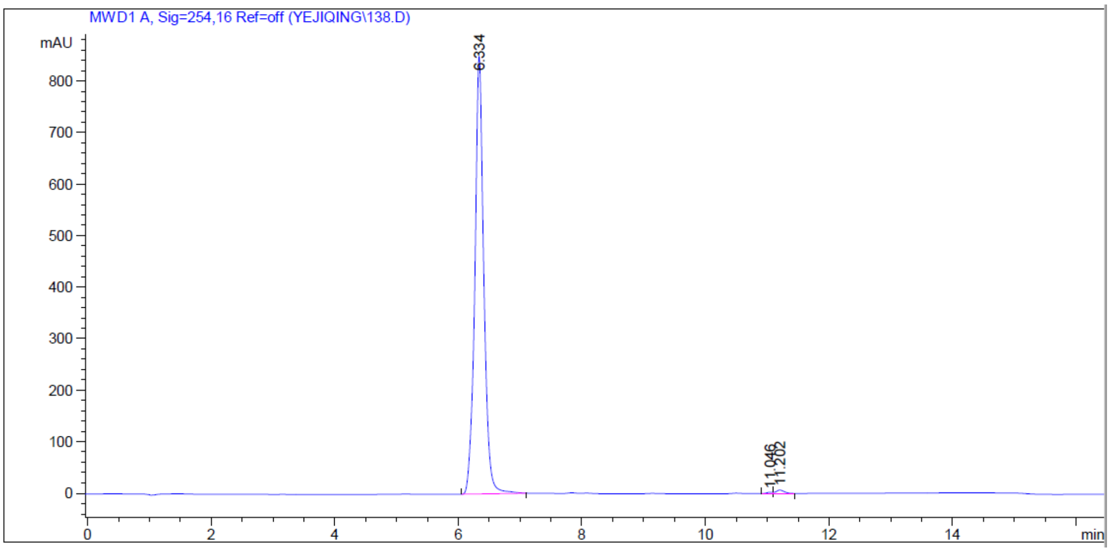

Peak #	RetTime [min]	Type	Width [min]	Area [mAU*s]	Height [mAU]	Area %
1	6.334	BB	0.1458	8582.63379	849.76947	99.1002
2	11.046	BV	0.0946	16.20356	2.60494	0.1871
3	11.202	VB	0.1382	61.72213	6.65700	0.7127

Totals : 8660.55947 859.03142

79

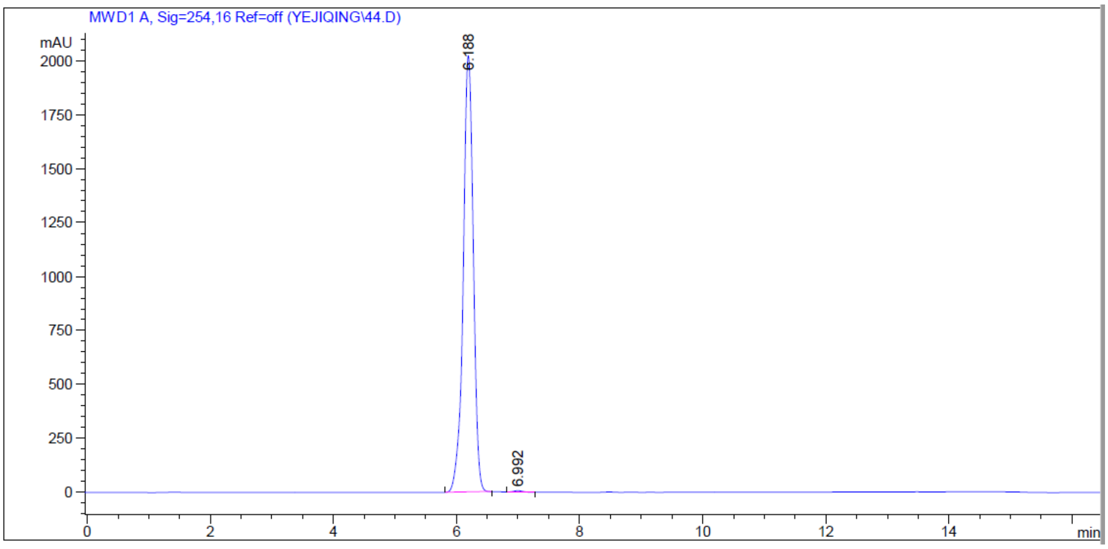

Peak #	RetTime [min]	Type	Width [min]	Area [mAU*s]	Height [mAU]	Area %
1	6.188	BB	0.1785	2.30003e4	2024.85046	99.7359
2	6.992	BB	0.1556	60.90421	6.25556	0.2641

Totals : 2.30612e4 2031.10603

80

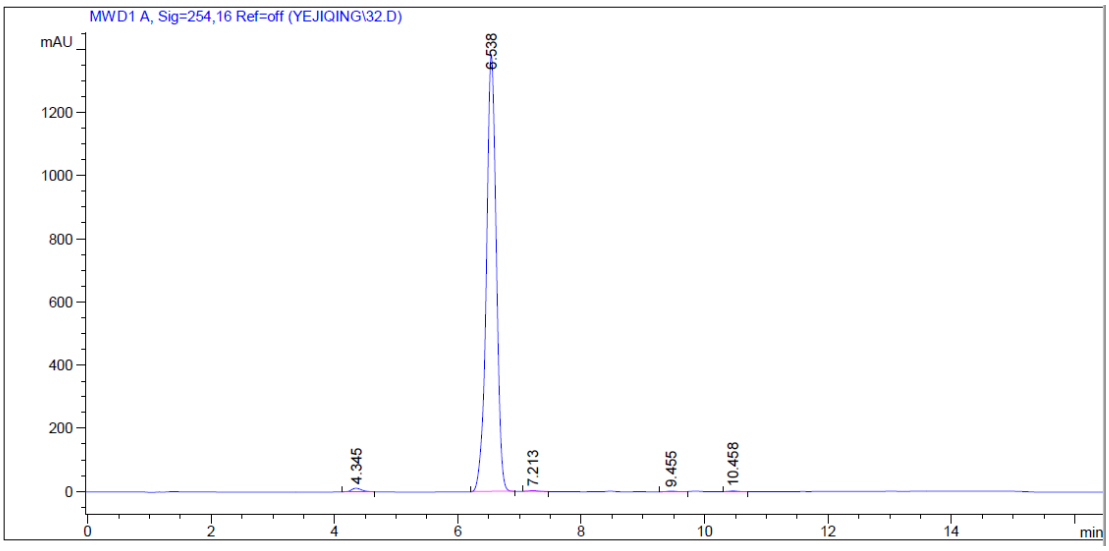

Peak #	RetTime [min]	Type	Width [min]	Area [mAU*s]	Height [mAU]	Area %
1	4.345	BB	0.1865	139.15605	11.55617	0.8901
2	6.538	BB	0.1741	1.54249e4	1383.21008	98.6647
3	7.213	BB	0.1560	28.77336	2.84431	0.1840
4	9.455	BB	0.1603	21.29120	2.06566	0.1362
5	10.458	BB	0.1440	19.54393	2.11151	0.1250
Totals :				1.56337e4	1401.78773	

81

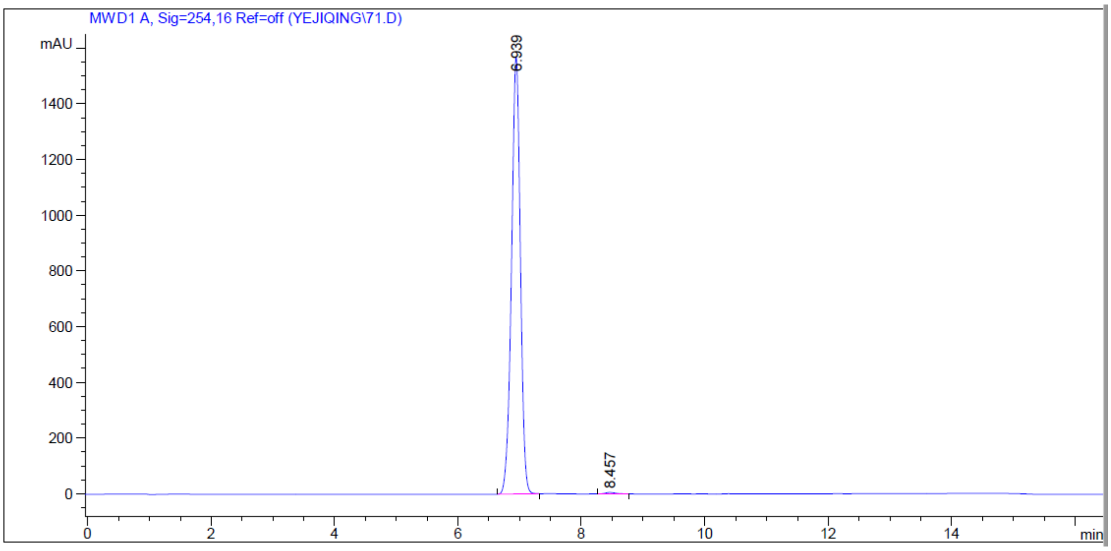

Peak #	RetTime [min]	Type	Width [min]	Area [mAU*s]	Height [mAU]	Area %
1	6.939	BB	0.1524	1.56789e4	1572.01453	99.5898
2	8.457	BB	0.1738	64.57551	5.46752	0.4102
Totals :				1.57434e4	1577.48205	

82

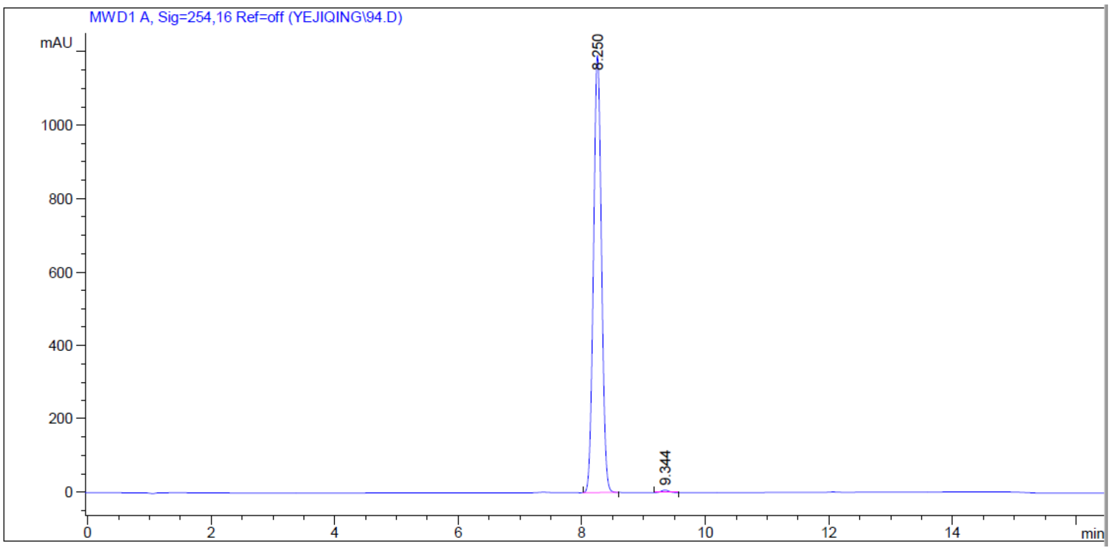

Peak #	RetTime [min]	Type	Width [min]	Area [mAU*s]	Height [mAU]	Area %
1	8.250	BB	0.1385	1.06853e4	1192.76758	99.4471
2	9.344	BB	0.1350	59.40331	6.86102	0.5529

Totals : 1.07447e4 1199.62860

83

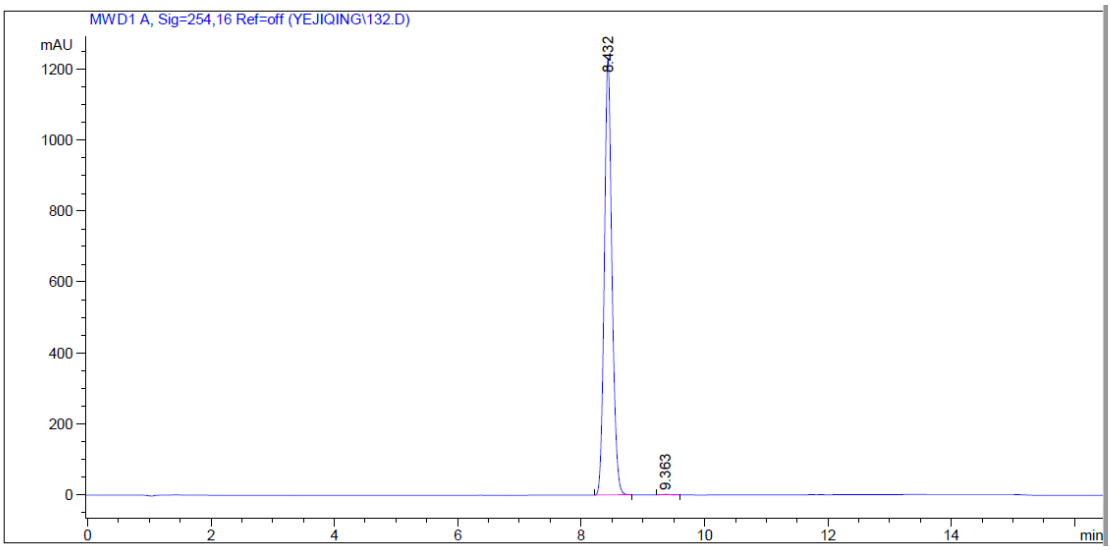

Peak #	RetTime [min]	Type	Width [min]	Area [mAU*s]	Height [mAU]	Area %
1	8.432	BB	0.1285	1.06219e4	1233.31104	99.8595
2	9.363	BB	0.1314	14.94627	1.75392	0.1405

Totals : 1.06368e4 1235.06496

84

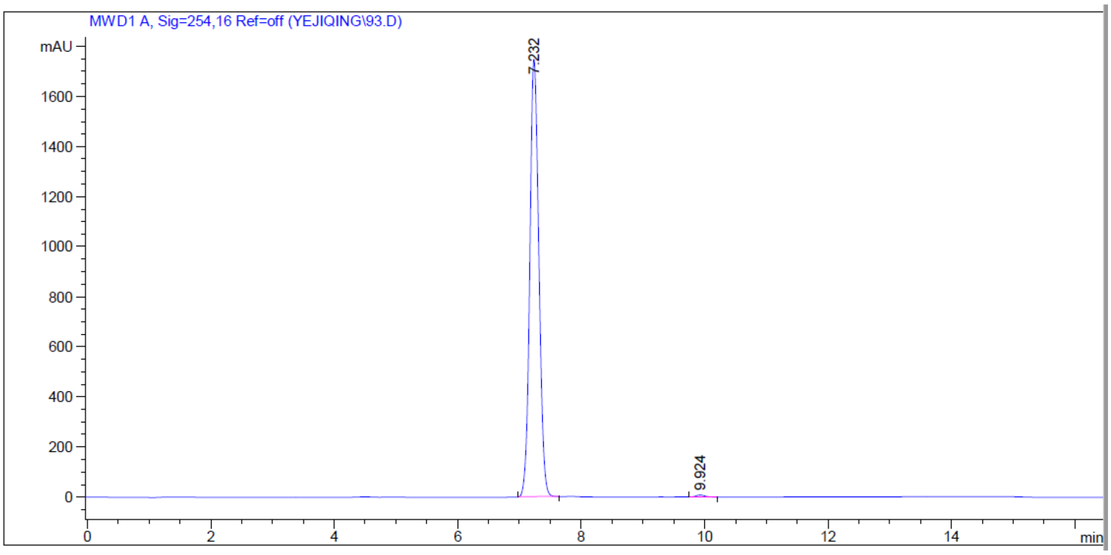

Peak #	RetTime [min]	Type	Width [min]	Area [mAU*s]	Height [mAU]	Area %
1	7.232	BB	0.1588	1.78121e4	1749.60913	99.6002
2	9.924	BB	0.1458	71.49633	7.74017	0.3998

Totals : 1.78836e4 1757.34930

85

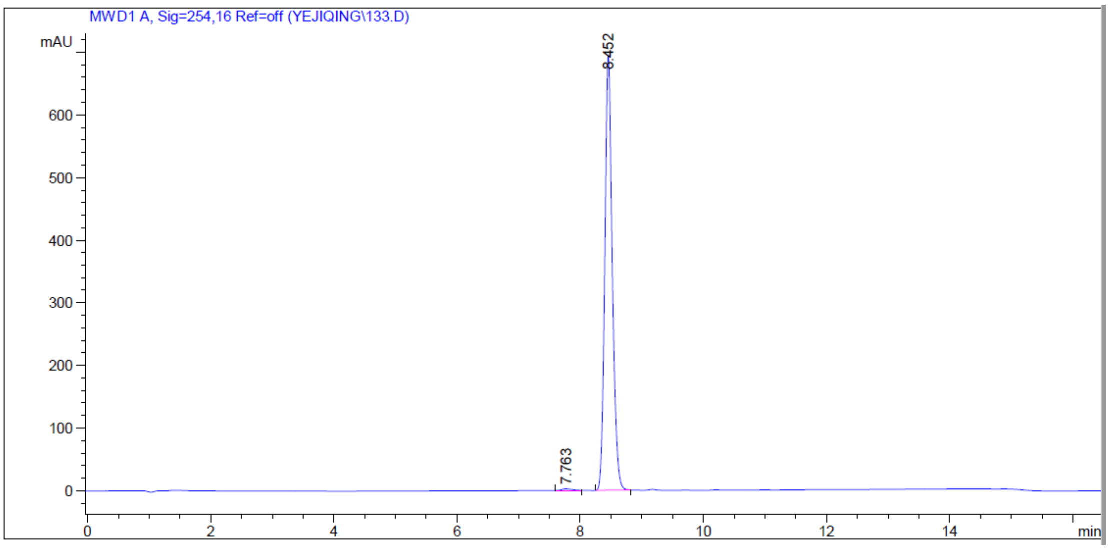

Peak #	RetTime [min]	Type	Width [min]	Area [mAU*s]	Height [mAU]	Area %
1	7.763	BB	0.1702	29.57688	2.61093	0.5038
2	8.452	BB	0.1261	5841.49902	694.70935	99.4962

Totals : 5871.07590 697.32028

86

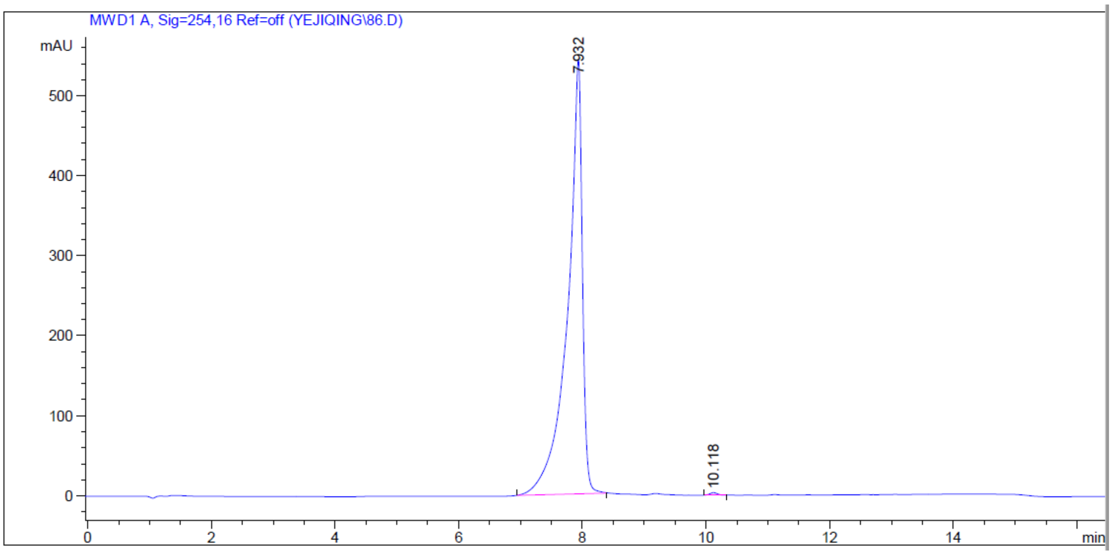

Peak #	RetTime [min]	Type	Width [min]	Area [mAU*s]	Height [mAU]	Area %
1	7.932	BB	0.2201	8949.56543	543.63312	99.7243
2	10.118	BB	0.1250	24.73917	3.10491	0.2757

Totals : 8974.30460 546.73803

87

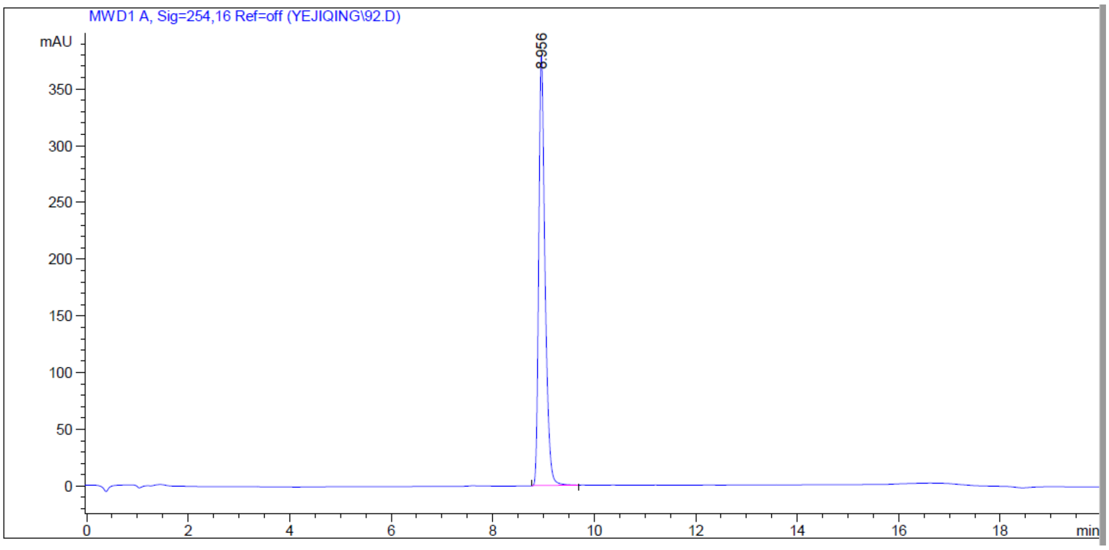

Peak #	RetTime [min]	Type	Width [min]	Area [mAU*s]	Height [mAU]	Area %
1	8.956	BB	0.1291	3292.24097	379.84518	100.0000

Totals : 3292.24097 379.84518

88

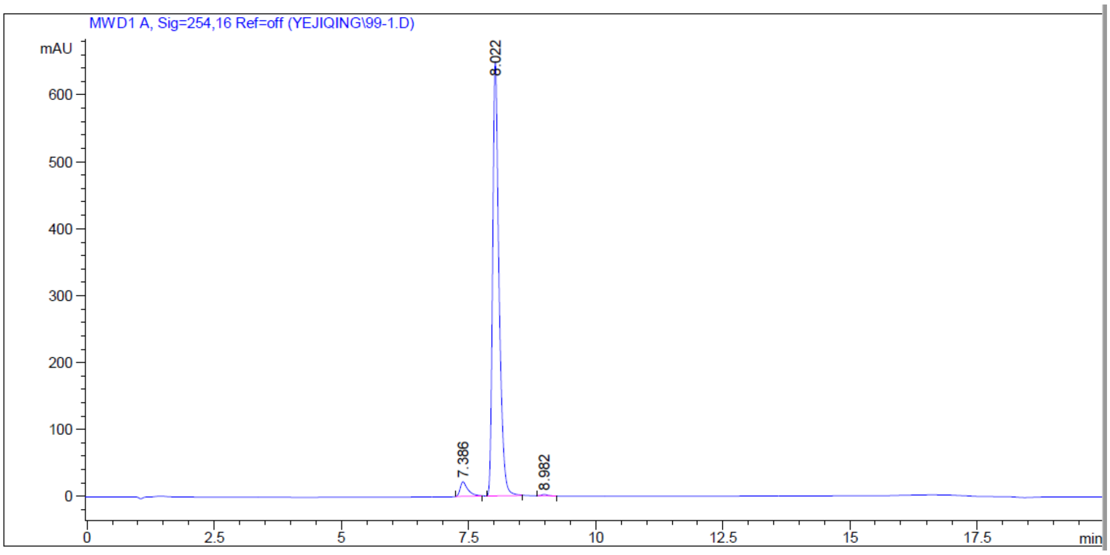

Peak #	RetTime [min]	Type	Width [min]	Area [mAU*s]	Height [mAU]	Area %
1	7.386	BB	0.1561	245.34422	22.34872	4.1260
2	8.022	BB	0.1300	5681.53271	649.87866	95.5464
3	8.982	BB	0.1265	19.48064	2.26081	0.3276

Totals : 5946.35758 674.48819

89

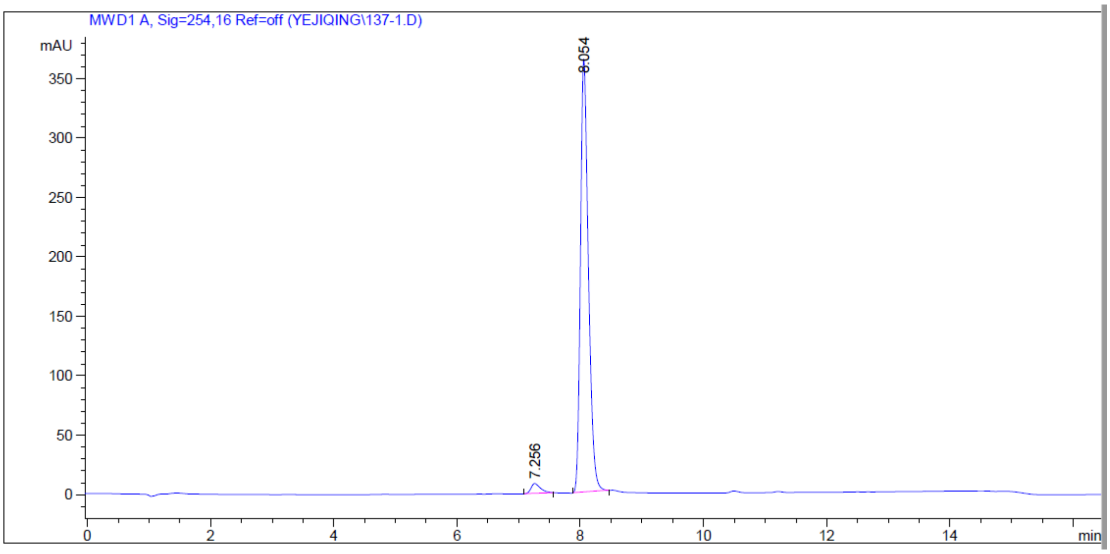

Peak #	RetTime [min]	Type	Width [min]	Area [mAU*s]	Height [mAU]	Area %
1	7.256	BB	0.1625	95.44690	8.52973	2.8418
2	8.054	BB	0.1344	3263.27393	364.79160	97.1582

Totals : 3358.72083 373.32133

90

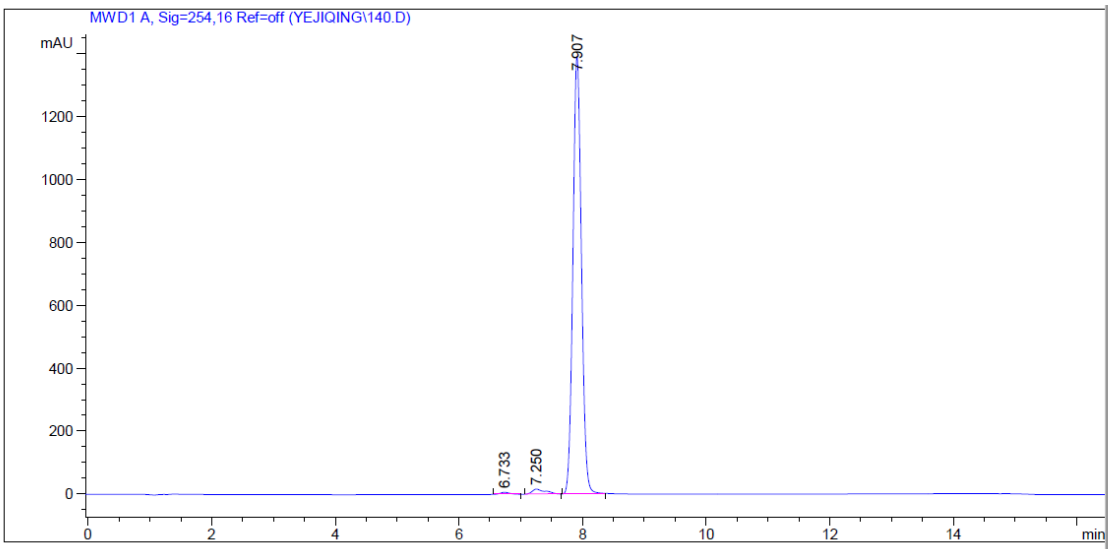

Peak #	RetTime [min]	Type	Width [min]	Area [mAU*s]	Height [mAU]	Area %
1	6.733	BB	0.1596	71.39988	6.74059	0.5251
2	7.250	BB	0.2094	244.78638	16.09445	1.8002
3	7.907	BB	0.1474	1.32814e4	1391.43933	97.6747

Totals : 1.35975e4 1414.27437

91

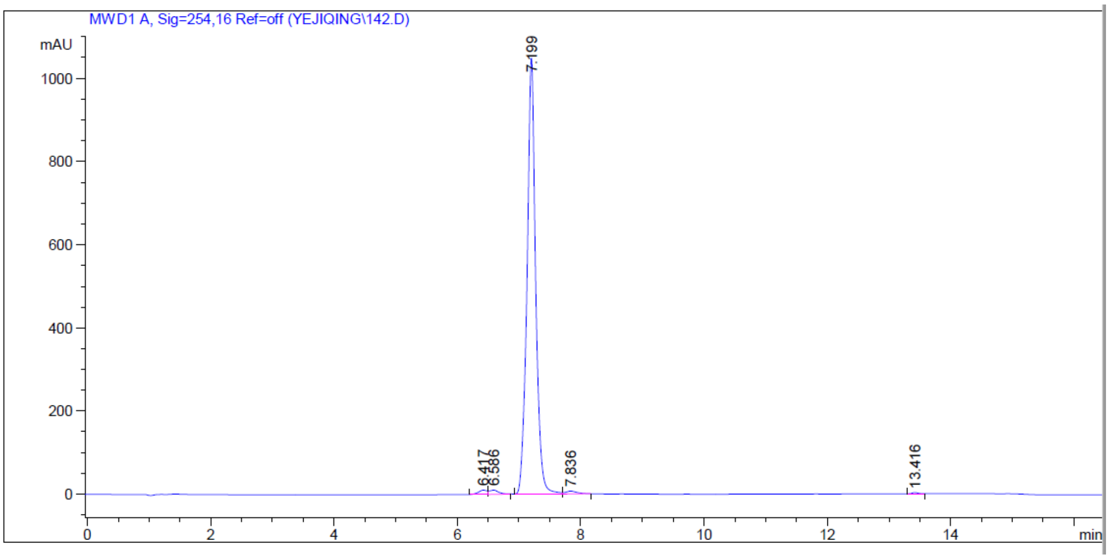

Peak #	RetTime [min]	Type	Width [min]	Area [mAU*s]	Height [mAU]	Area %
1	6.417	BV	0.1443	108.13895	10.66418	1.0267
2	6.586	VB	0.1531	114.61176	10.52213	1.0882
3	7.199	BB	0.1413	1.01930e4	1050.01892	96.7770
4	7.836	BB	0.1888	98.56856	7.24295	0.9359
5	13.416	BB	0.0975	18.14188	2.80240	0.1722

Totals : 1.05324e4 1081.25058

92

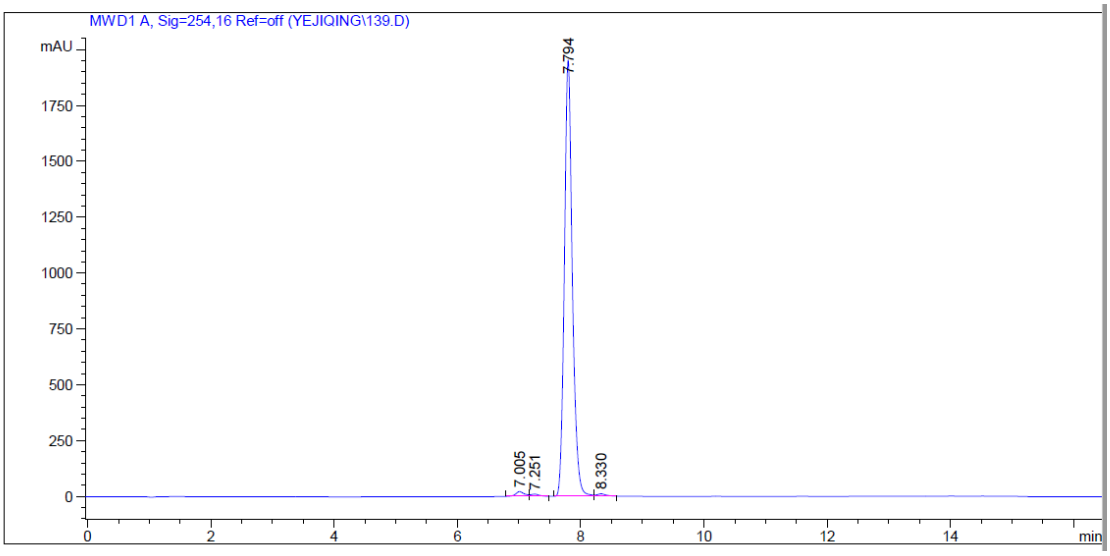

Peak #	RetTime [min]	Type	Width [min]	Area [mAU*s]	Height [mAU]	Area %
1	7.005	BV	0.1445	222.60387	21.91296	1.2397
2	7.251	VB	0.1407	103.47771	10.34506	0.5763
3	7.794	BV	0.1326	1.75176e4	1954.65161	97.5579
4	8.330	VB	0.1489	112.42438	10.84720	0.6261

Totals : 1.79561e4 1997.75683

93

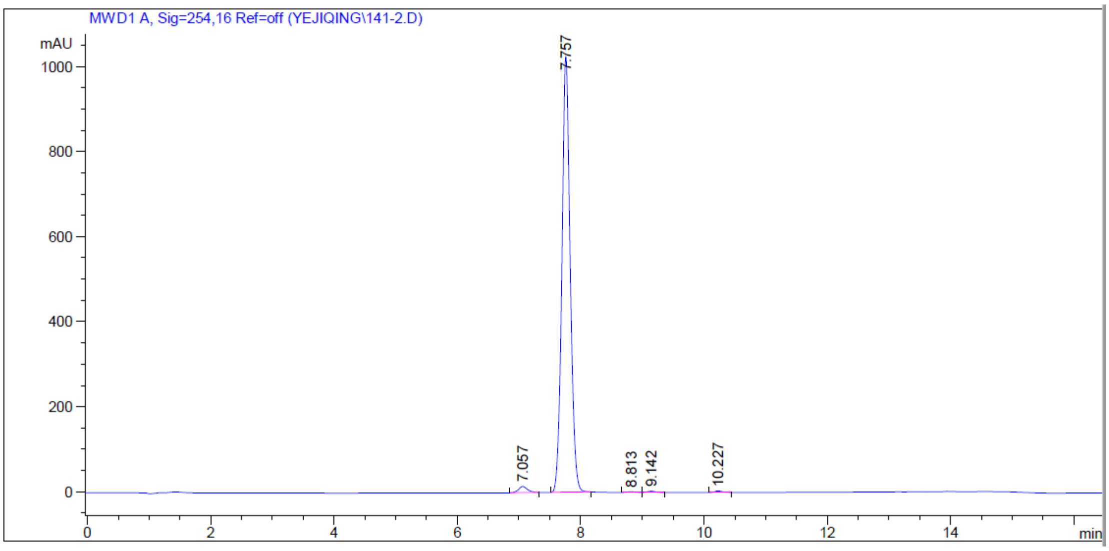

Peak #	RetTime [min]	Type	Width [min]	Area [mAU*s]	Height [mAU]	Area %
1	7.057	BB	0.1458	149.49164	14.80130	1.5296
2	7.757	BB	0.1366	9549.90234	1026.13940	97.7145
3	8.813	BV	0.1282	15.37492	1.82578	0.1573
4	9.142	VB	0.1282	27.65611	3.28657	0.2830
5	10.227	BB	0.1271	30.84468	3.70527	0.3156

Totals : 9773.26968 1049.75833

94

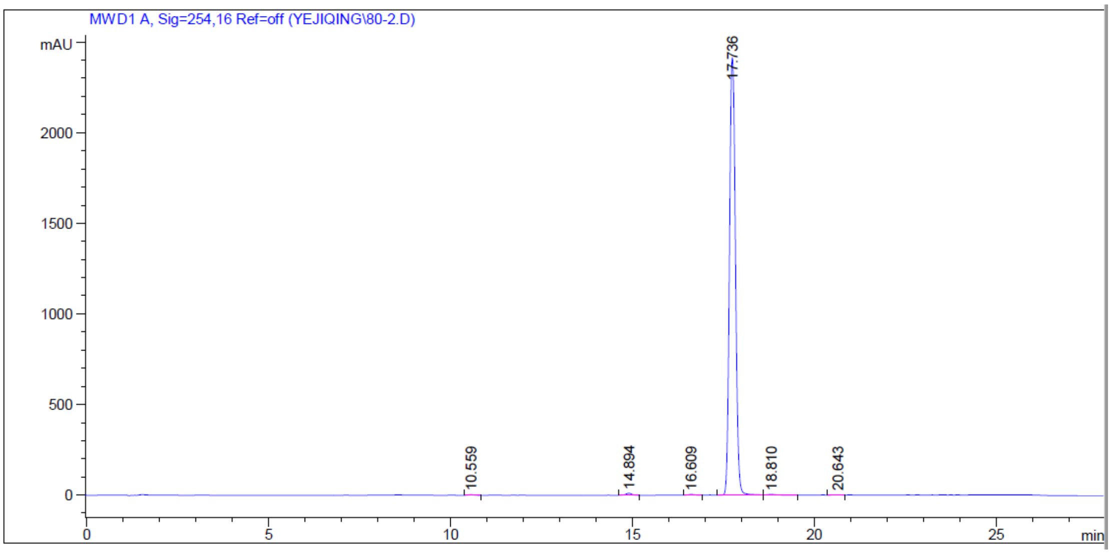

Peak #	RetTime [min]	Type	Width [min]	Area [mAU*s]	Height [mAU]	Area %
1	10.559	BB	0.1543	33.55360	3.36455	0.1205
2	14.894	BB	0.1743	129.72356	11.10168	0.4658
3	16.609	BB	0.1772	50.76182	4.25377	0.1823
4	17.736	VB	0.1809	2.75338e4	2416.37549	98.8706
5	18.810	BB	0.2365	70.77885	4.33360	0.2542
6	20.643	VV	0.2102	29.70411	2.03649	0.1067

Totals : 2.78483e4 2441.46557

95

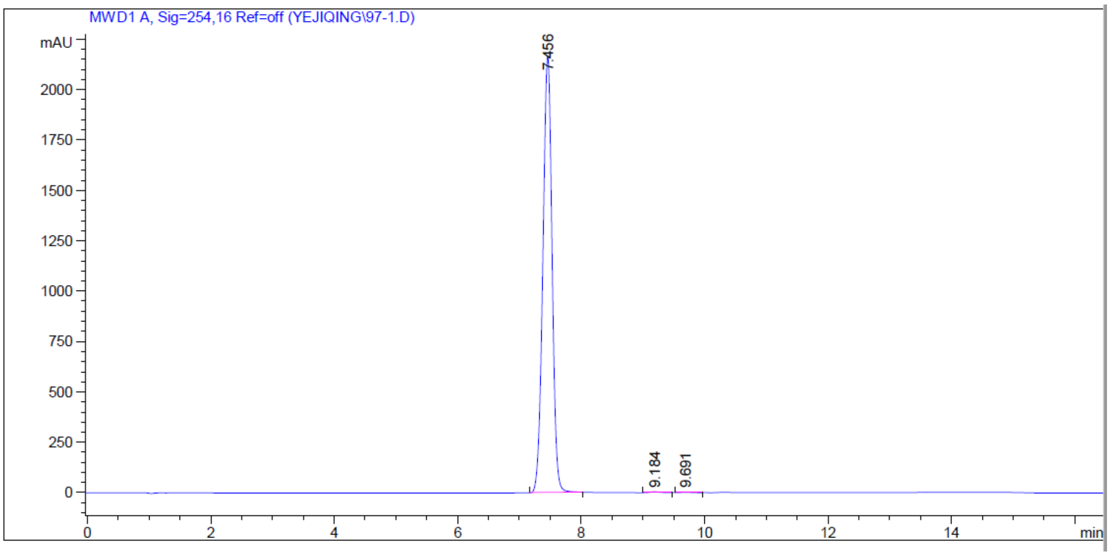

Peak #	RetTime [min]	Type	Width [min]	Area [mAU*s]	Height [mAU]	Area %
1	7.456	BB	0.1609	2.24511e4	2166.79468	99.7071
2	9.184	BB	0.1645	45.74073	4.15185	0.2031
3	9.691	BB	0.1482	20.22065	2.17958	0.0898

Totals : 2.25171e4 2173.12611

qNMR spectroscopic data

22

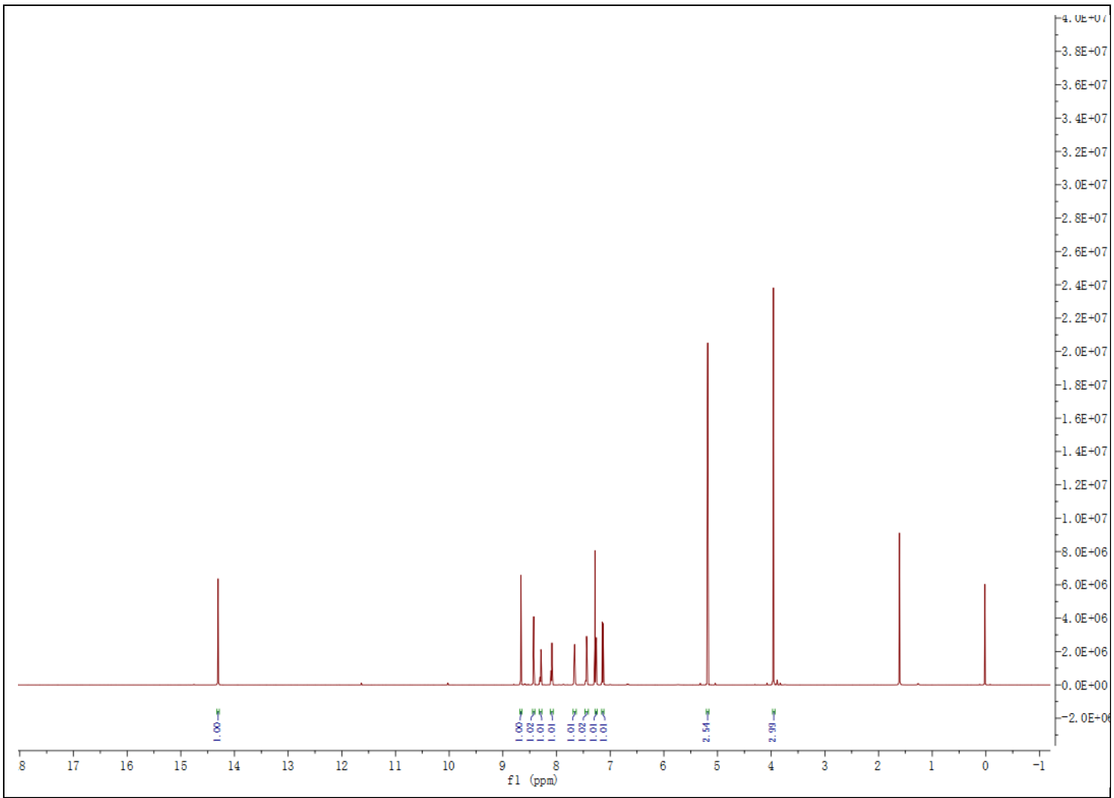

m_s = 5.23 mg, m_{IC} = 0.649 mg , P_{IC} = 99.66%
Int_t = 1.01, n_t = 1
Int_{IC} = 2.54, n_{IC} = 6
MW_t = 300.27 g/mol, MW_{IC} = 90.08 g/mol
$P\,[\%] = \frac{n_{IC} * Int_t * MW_t * m_{IC}}{n_t * Int_{IC} * MW_{IC} * m_s} * P_{IC} = 98.4\%$

24

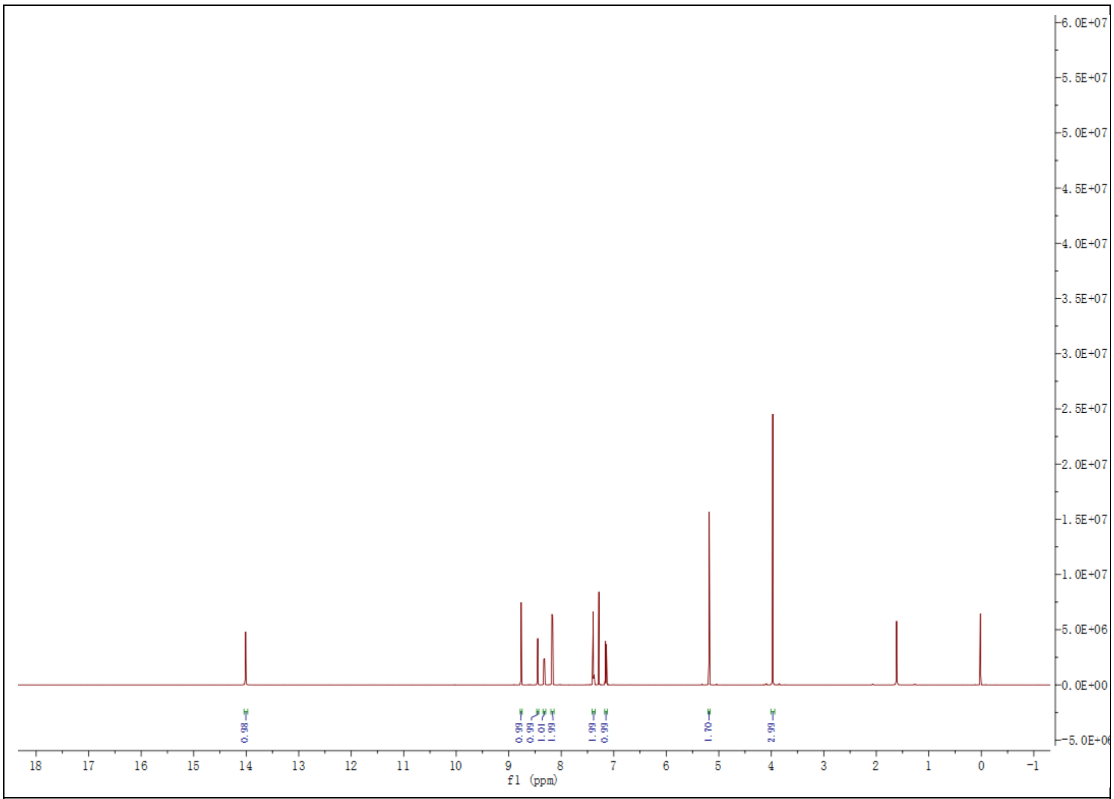

m_s = 5.27 mg, m_{IC} = 0.449 mg , P_{IC} = 99.66%
Int_t = 0.99, n_t = 1
Int_{IC} = 1.70, n_{IC} = 6
MW_t = 300.27 g/mol, MW_{IC} = 90.08 g/mol
$P\,[\%] = \frac{n_{IC} * Int_t * MW_t * m_{IC}}{n_t * Int_{IC} * MW_{IC} * m_s} * P_{IC} = 98.9\%$

27

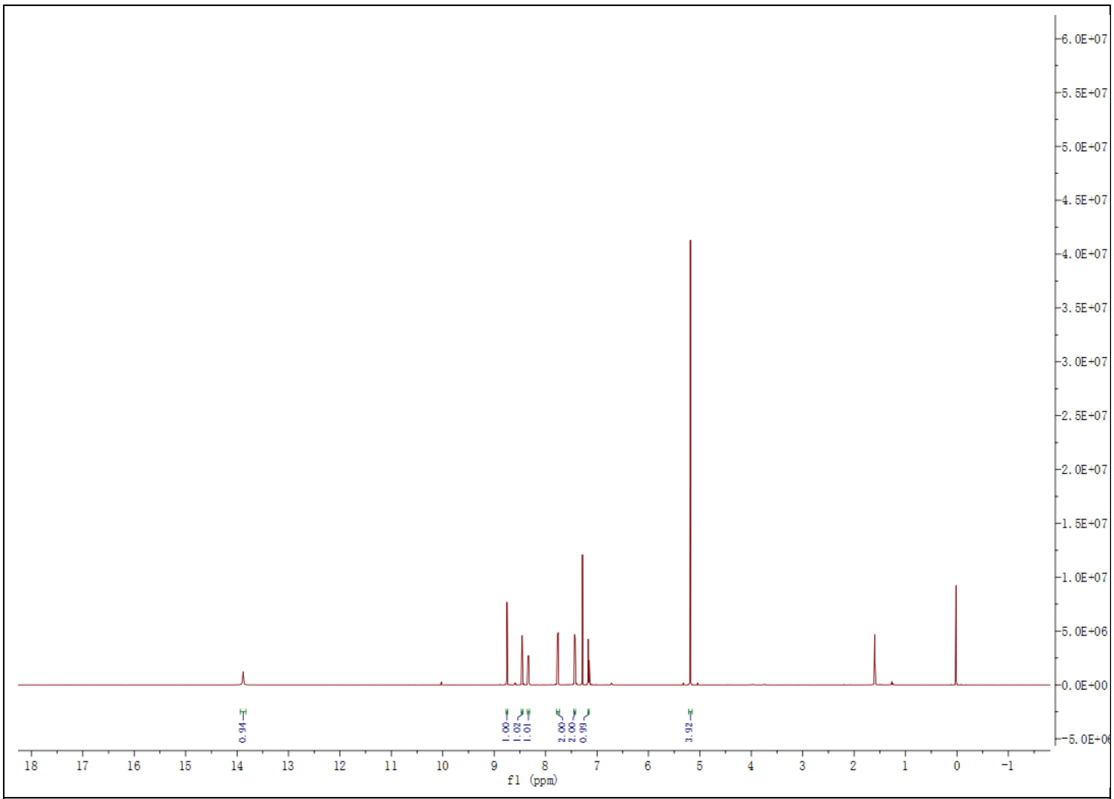

m_s = 5.12 mg, m_{IC} = 0.948 mg , P_{IC} = 99.66%
Int_t = 1.00, n_t = 1
Int_{IC} = 3.92, n_{IC} = 6
MW_t = 310.23 g/mol, MW_{IC} = 90.08 g/mol
$P\,[\%] = \frac{n_{IC} * Int_t * MW_t * m_{IC}}{n_t * Int_{IC} * MW_{IC} * m_s} * P_{IC} = 97.2994 = 97.3\%$

33

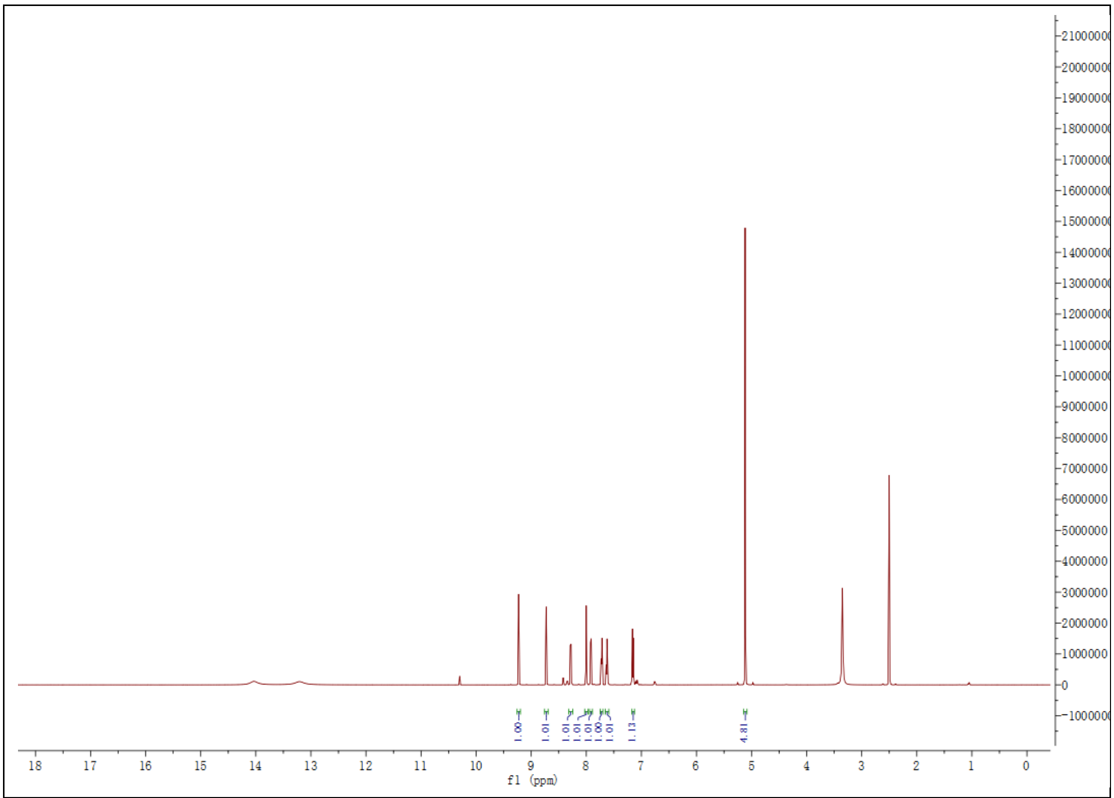

m_s = 4.86 mg, m_{IC} = 1.098 mg , P_{IC} = 99.66%
Int_t = 1.02, n_t = 1
Int_{IC} = 4.81, n_{IC} = 6
MW_t = 286.24 g/mol, MW_{IC} = 90.08 g/mol
$P\,[\%] = \frac{n_{IC} * Int_t * MW_t * m_{IC}}{n_t * Int_{IC} * MW_{IC} * m_s} * P_{IC} = 91.0\%$

74

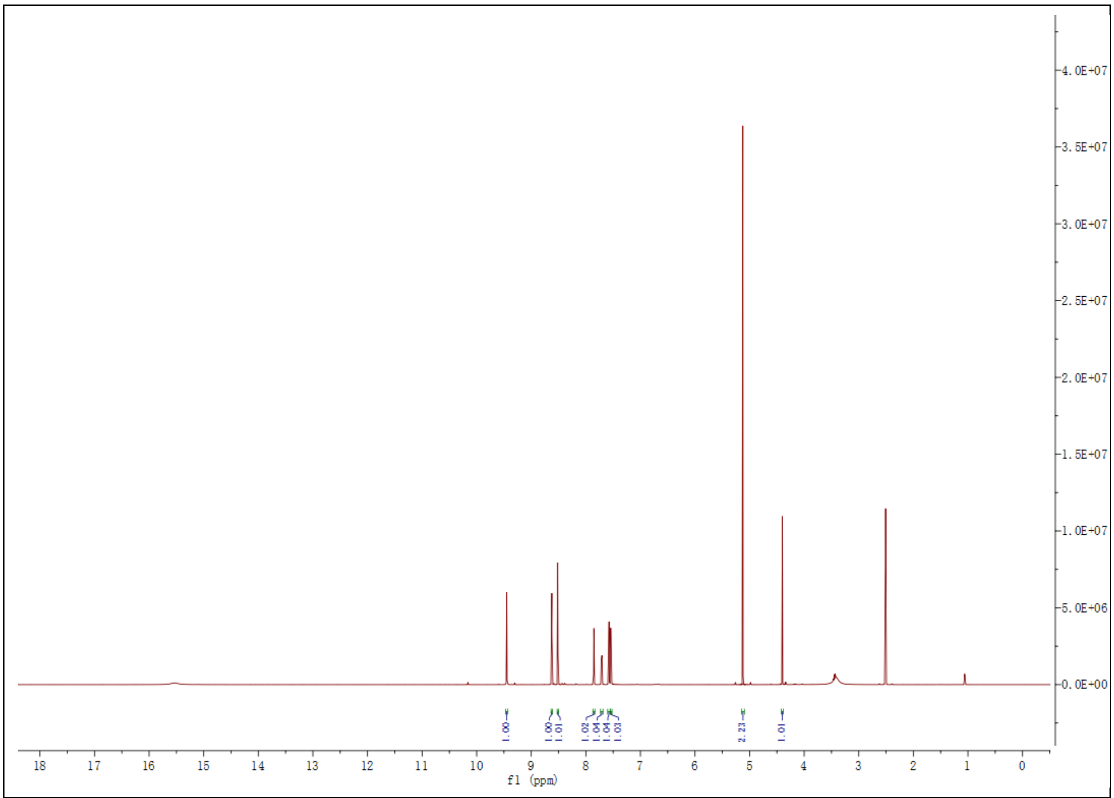

m_s = 5.89 mg, m_{IC} = 0.549 mg , P_{IC} = 99.66%
Int_t = 1.02, n_t = 1
Int_{IC} = 2.23, n_{IC} = 6
MW_t = 345.15 g/mol, MW_{IC} = 90.08 g/mol
$P\,[\%] = \frac{n_{IC} * Int_t * MW_t * m_{IC}}{n_t * Int_{IC} * MW_{IC} * m_s} * P_{IC} = 97.7\%$

HRMS profiles

1

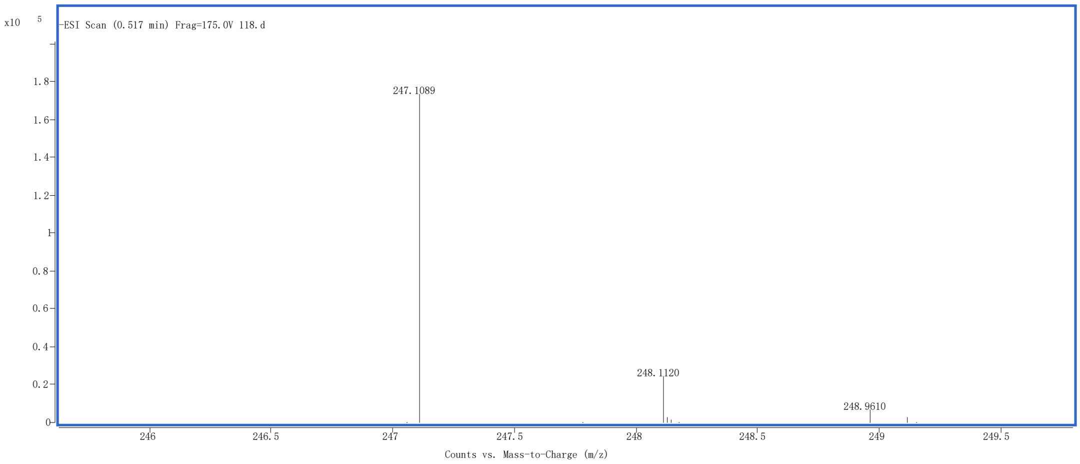

2

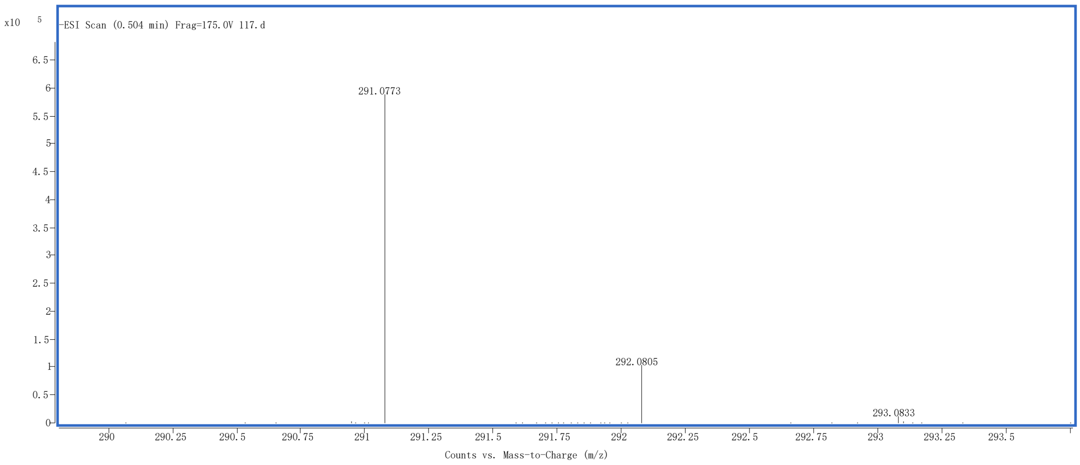

3

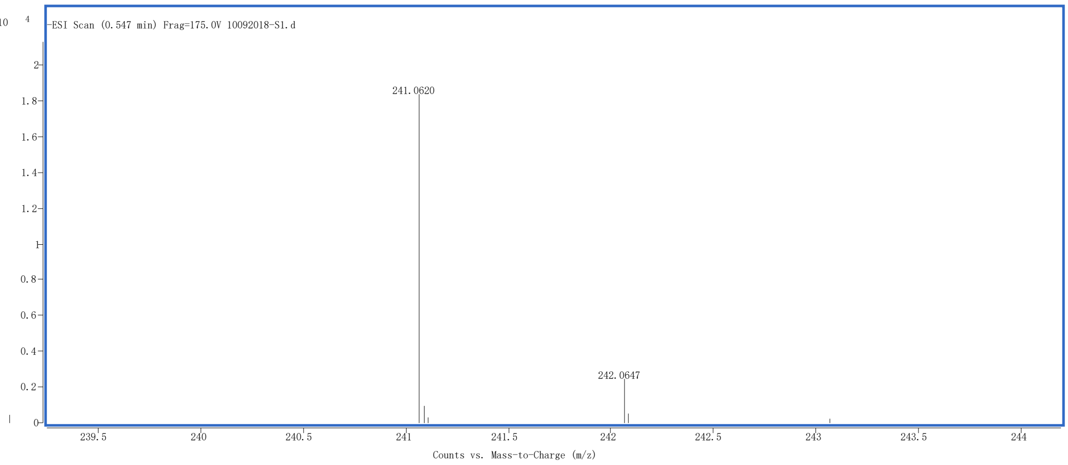

4

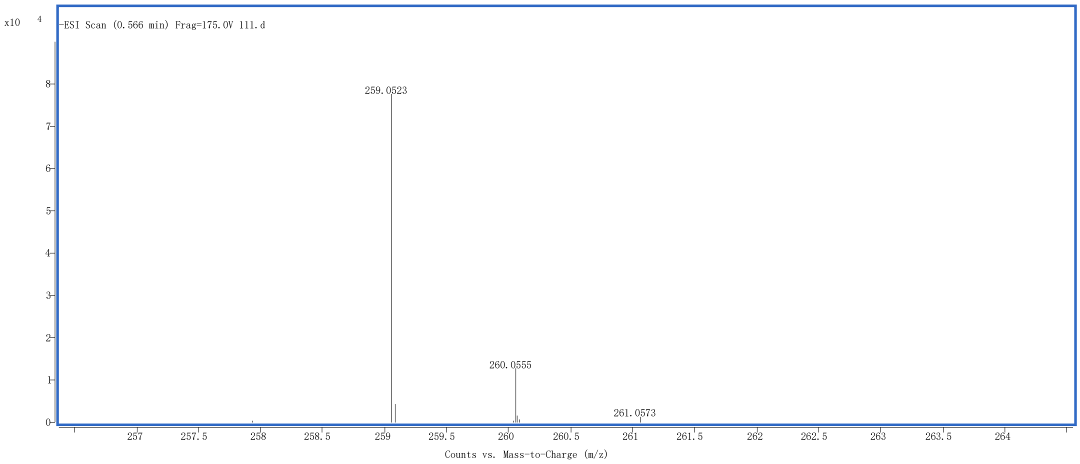
x10 4
ESI Scan (0.566 min) Frag=175.0V 111.d
259.0523
260.0555
261.0573
Counts vs. Mass-to-Charge (m/z)

5

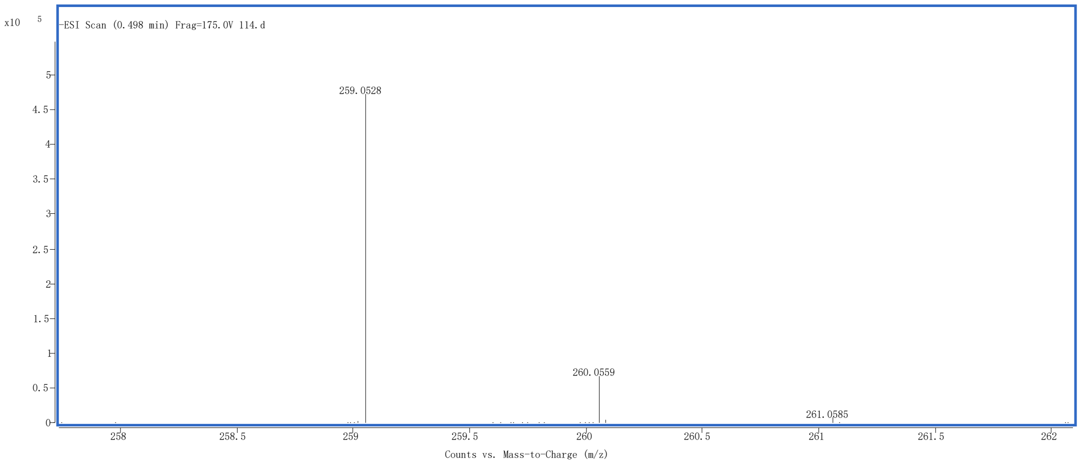
x10 5
ESI Scan (0.498 min) Frag=175.0V 114.d
259.0528
260.0559
261.0585
Counts vs. Mass-to-Charge (m/z)

6

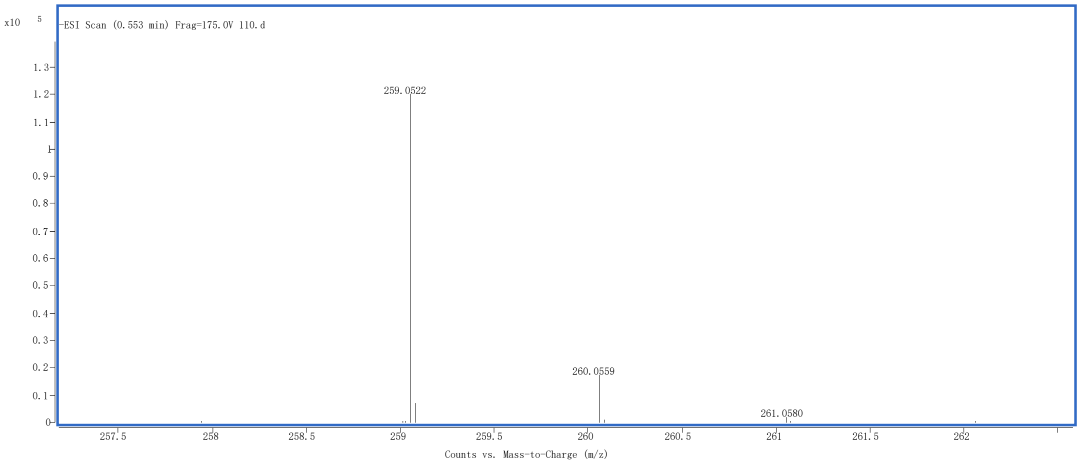
x10 5
ESI Scan (0.553 min) Frag=175.0V 110.d
259.0522
260.0559
261.0580
Counts vs. Mass-to-Charge (m/z)

7

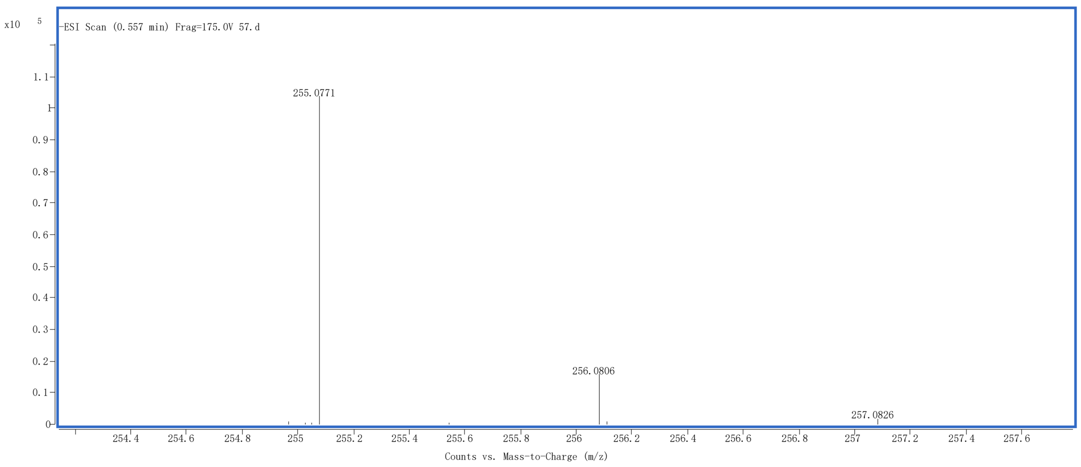
x10 5
ESI Scan (0.557 min) Frag=175.0V 57.d
255.0771
256.0806
257.0826
Counts vs. Mass-to-Charge (m/z)

8

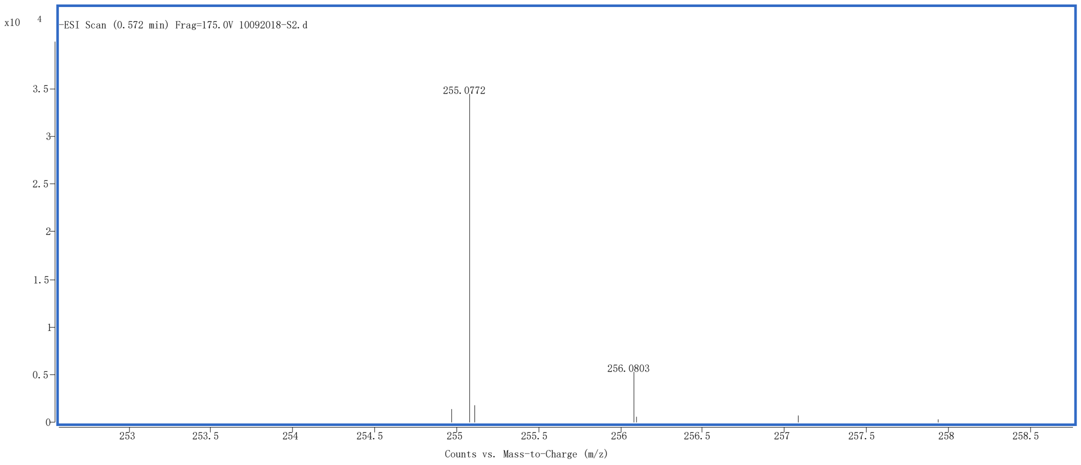
x10 4
ESI Scan (0.572 min) Frag=175.0V 10092018-S2.d
255.0772
256.0803
Counts vs. Mass-to-Charge (m/z)

9

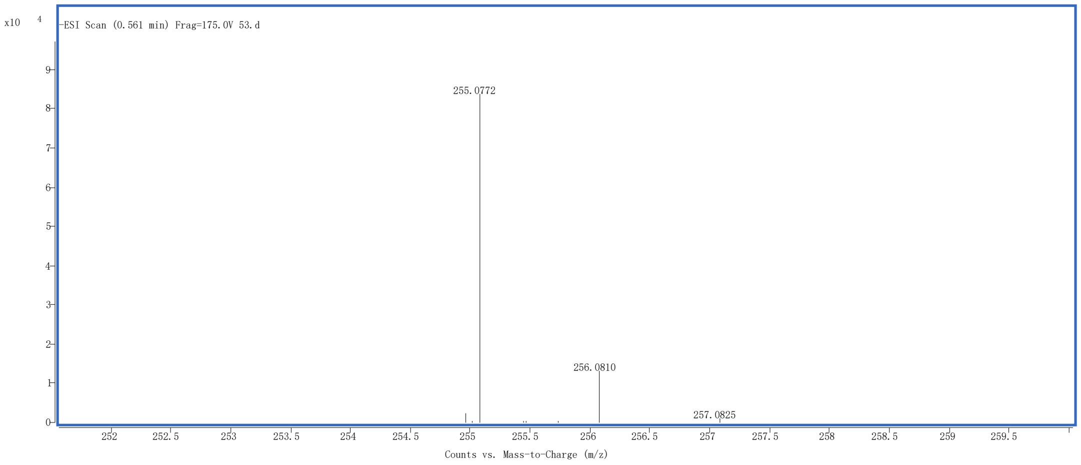
x10 4
ESI Scan (0.561 min) Frag=175.0V 53.d
255.0772
256.0810
257.0825
Counts vs. Mass-to-Charge (m/z)

10

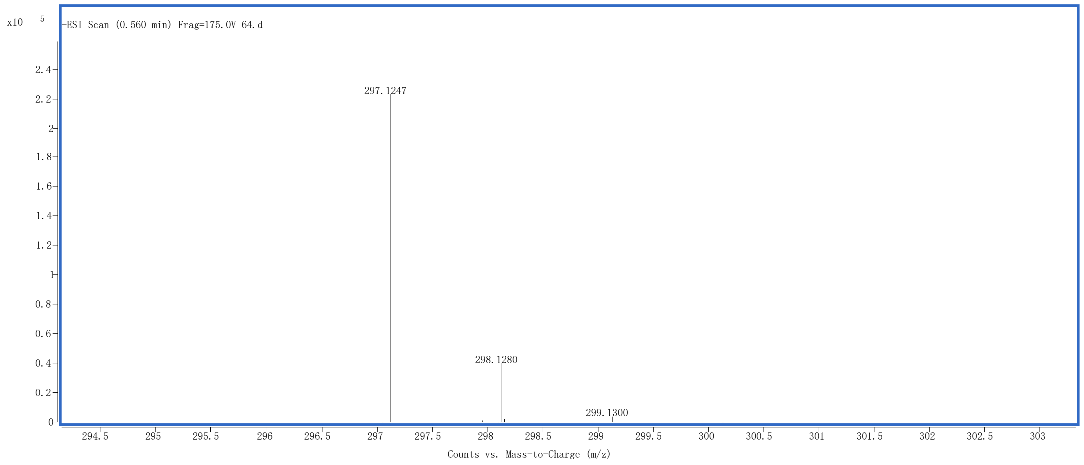
x10 5
ESI Scan (0.560 min) Frag=175.0V 64.d
297.1247
298.1280
299.1300
Counts vs. Mass-to-Charge (m/z)

11

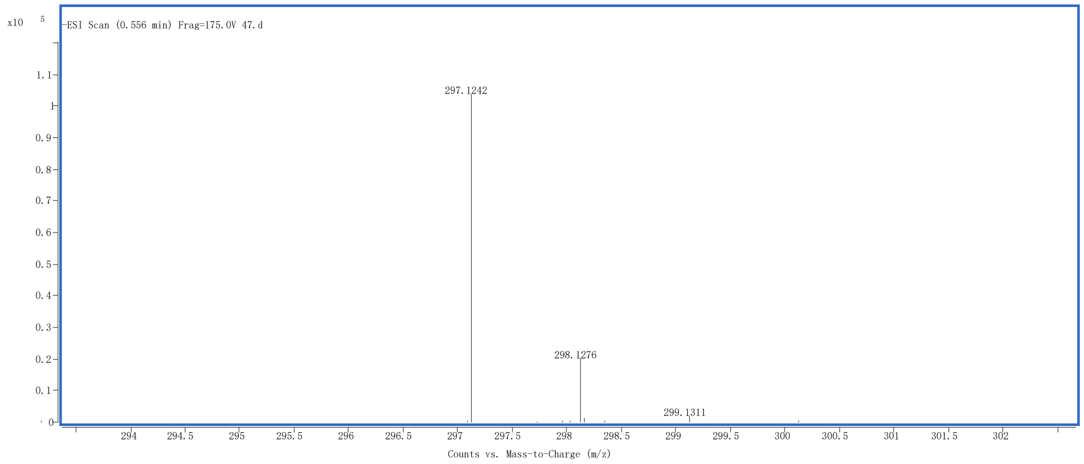
x10 5
ESI Scan (0.556 min) Frag=175.0V 47.d
297.1242
298.1276
299.1311
Counts vs. Mass-to-Charge (m/z)

12

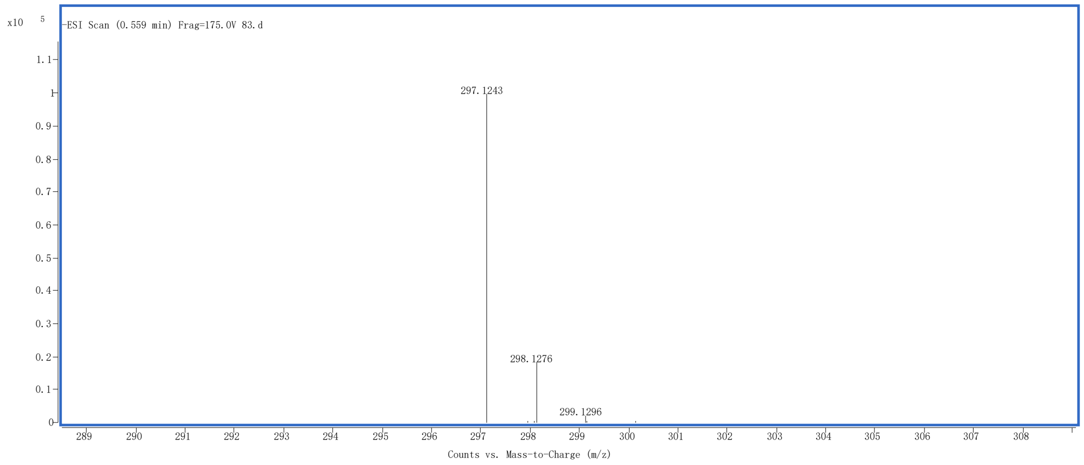
x10 5
ESI Scan (0.559 min) Frag=175.0V 83.d
297.1243
298.1276
299.1296
Counts vs. Mass-to-Charge (m/z)

13

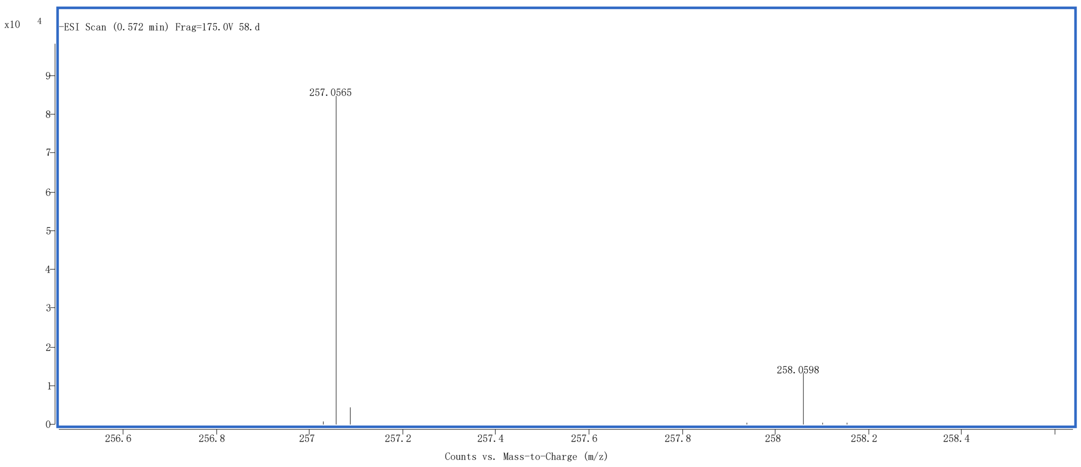
x10 4
ESI Scan (0.572 min) Frag=175.0V 58.d
257.0565
258.0598
Counts vs. Mass-to-Charge (m/z)

14

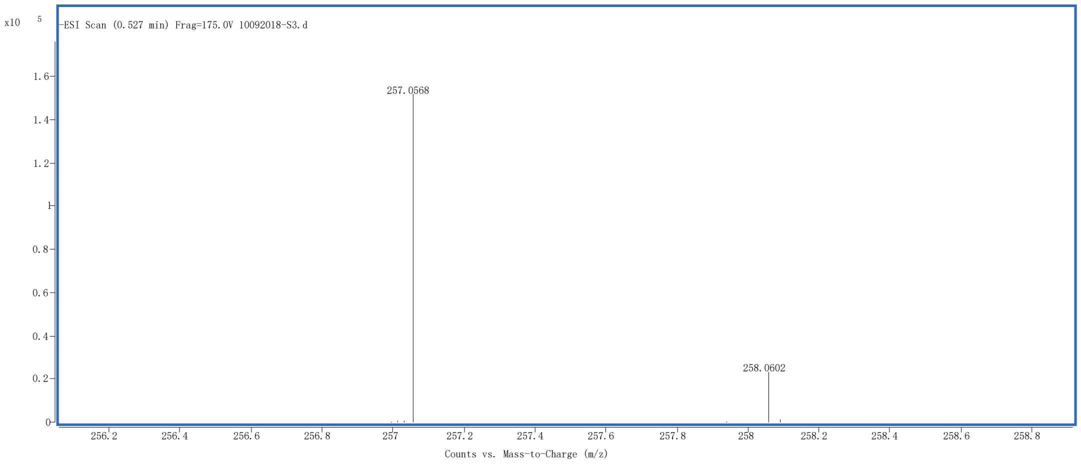
x10 5
ESI Scan (0.527 min) Frag=175.0V 10092018-S3.d
257.0568
258.0602
Counts vs. Mass-to-Charge (m/z)

15

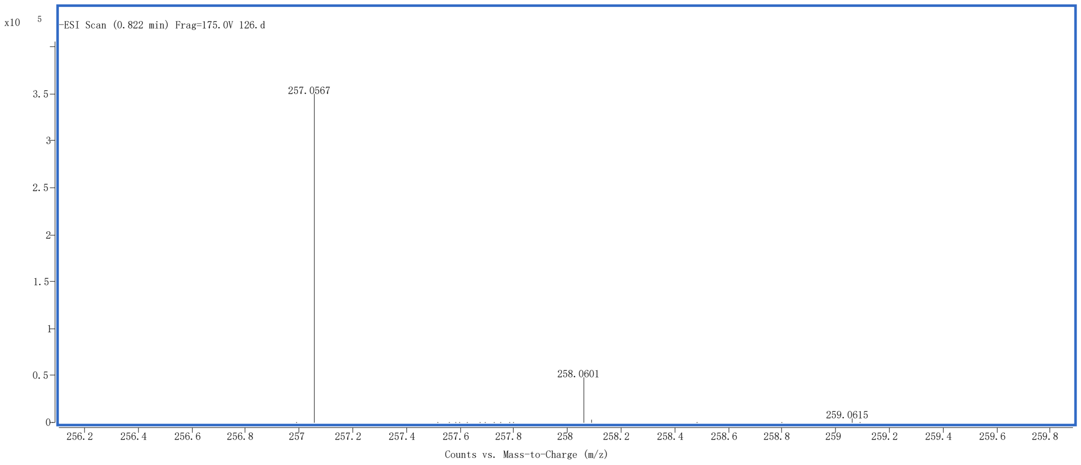
x10 5
ESI Scan (0.822 min) Frag=175.0V 126.d
257.0567
258.0601
259.0615
Counts vs. Mass-to-Charge (m/z)

16

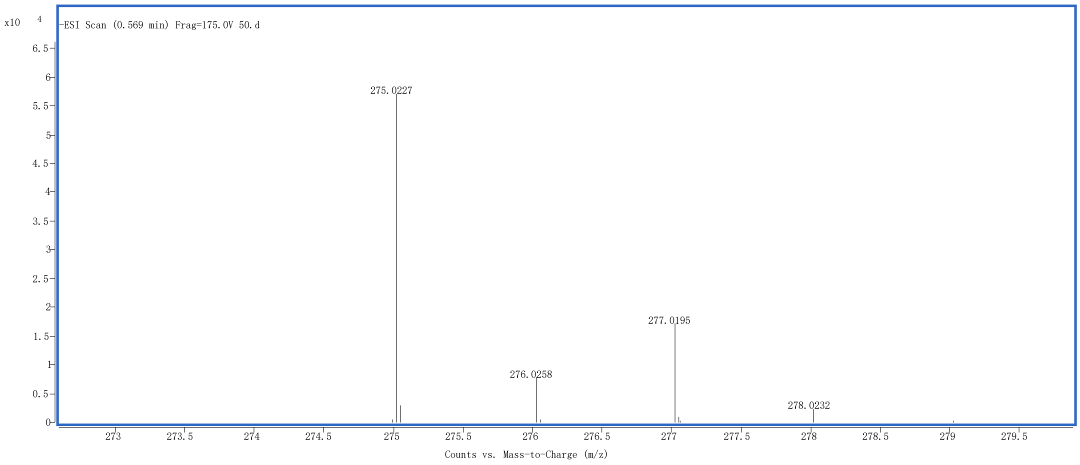
x10 4
ESI Scan (0.569 min) Frag=175.0V 50.d
275.0227
276.0258
277.0195
278.0232
Counts vs. Mass-to-Charge (m/z)

17

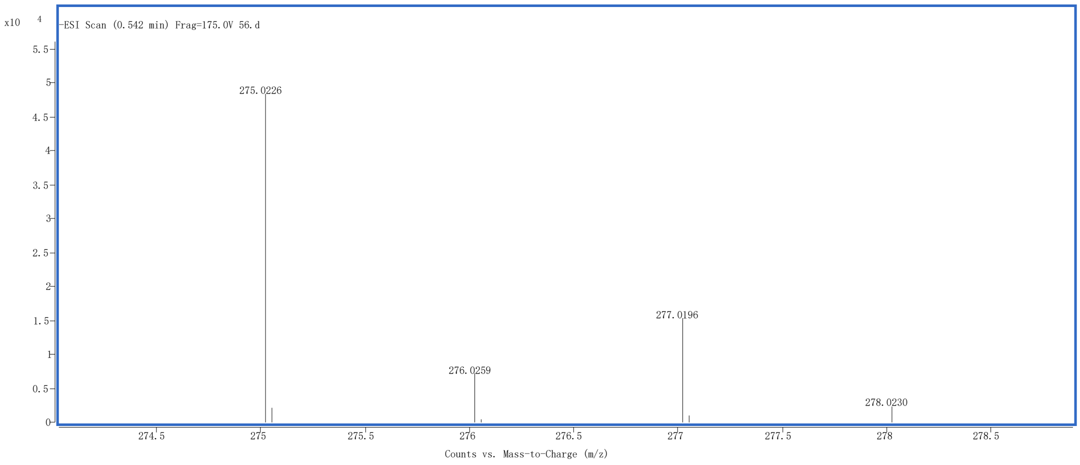
x10 4
ESI Scan (0.542 min) Frag=175.0V 56.d
275.0226
276.0259
277.0196
278.0230
Counts vs. Mass-to-Charge (m/z)

18

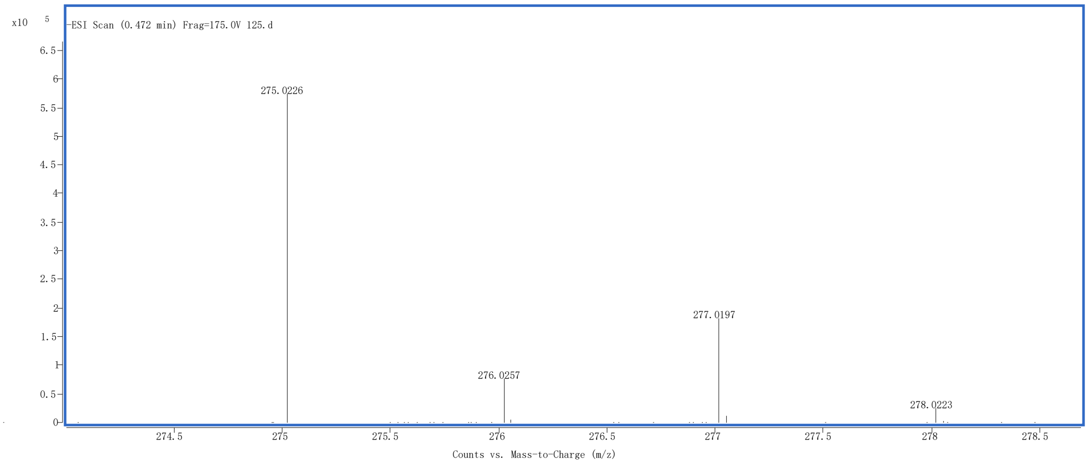
x10 5
ESI Scan (0.472 min) Frag=175.0V 125.d
275.0226
276.0257
277.0197
278.0223
Counts vs. Mass-to-Charge (m/z)

19

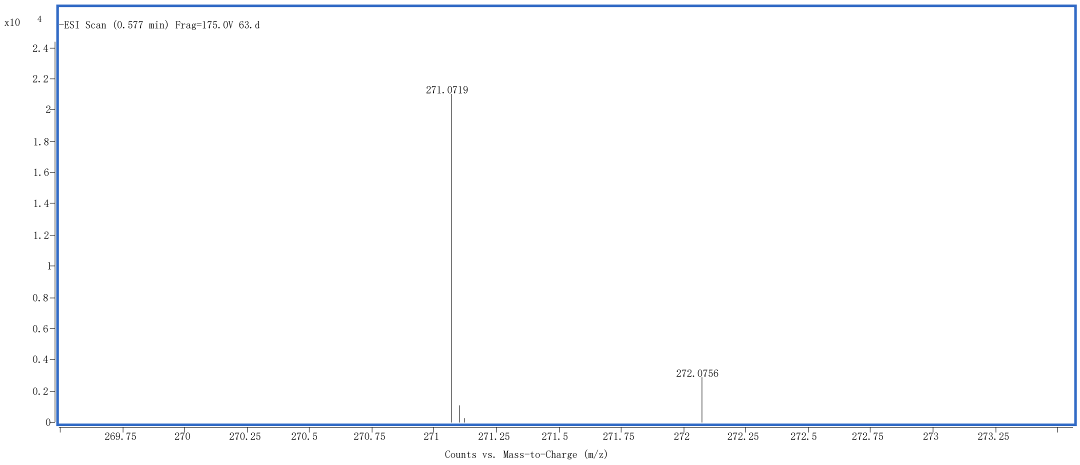
x10 4
ESI Scan (0.577 min) Frag=175.0V 63.d
271.0719
272.0756
Counts vs. Mass-to-Charge (m/z)

20

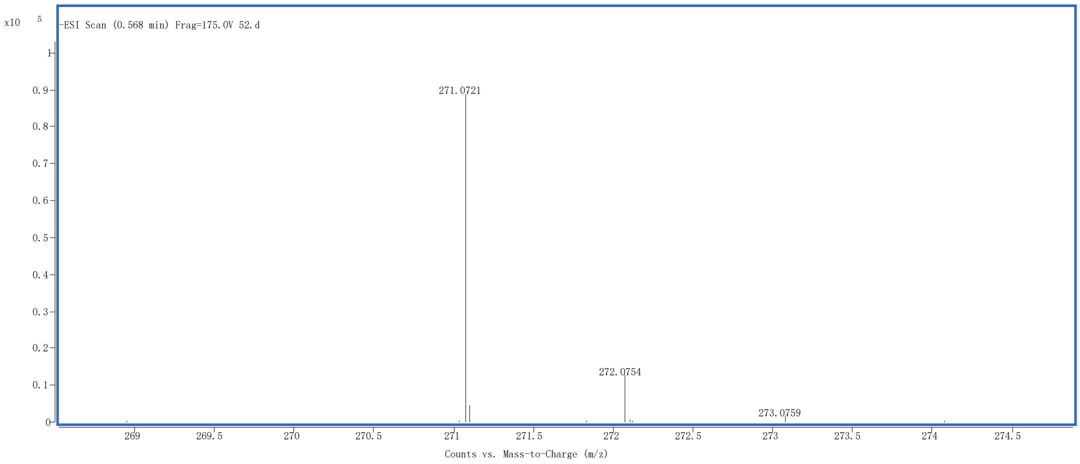
x10 5
ESI Scan (0.568 min) Frag=175.0V 52.d
271.0721
272.0754
273.0759
Counts vs. Mass-to-Charge (m/z)

21

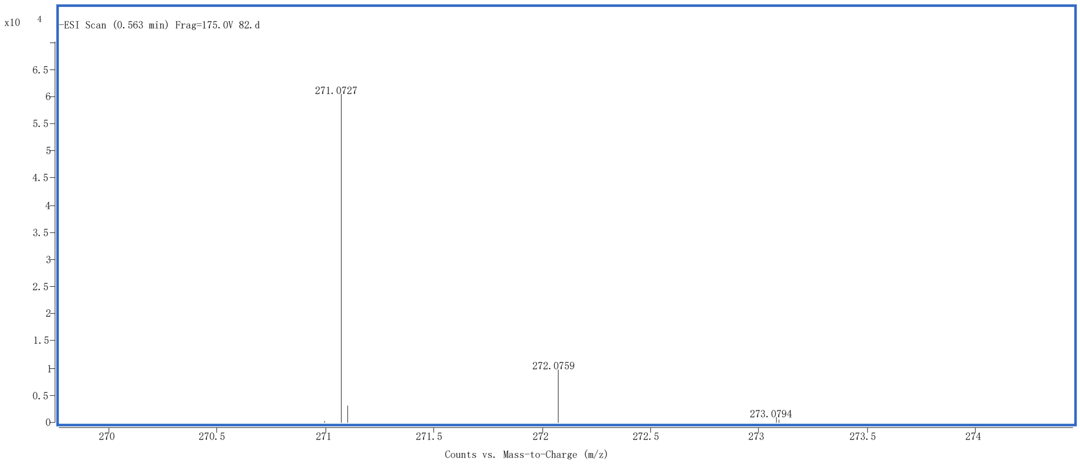
x10 4
ESI Scan (0.563 min) Frag=175.0V 82.d
271.0727
272.0759
273.0794
Counts vs. Mass-to-Charge (m/z)

22

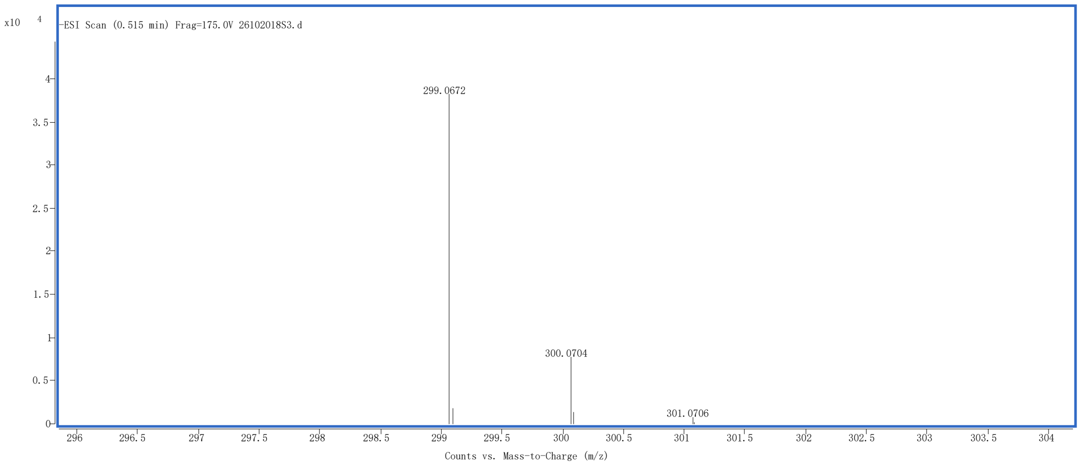
x10 4
-ESI Scan (0.515 min) Frag=175.0V 26102018S3.d
299.0672
300.0704
301.0706
Counts vs. Mass-to-Charge (m/z)

23

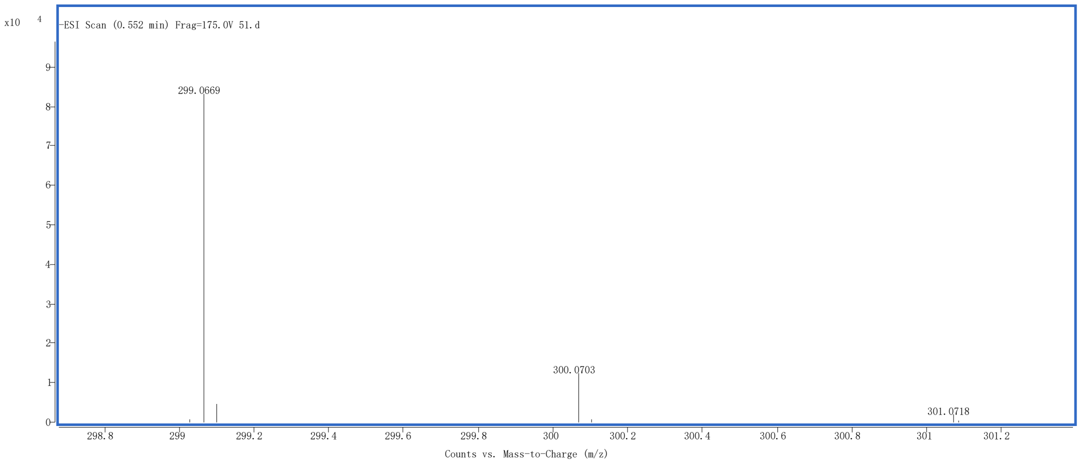
x10 4
-ESI Scan (0.552 min) Frag=175.0V 51.d
299.0669
300.0703
301.0718
Counts vs. Mass-to-Charge (m/z)

24

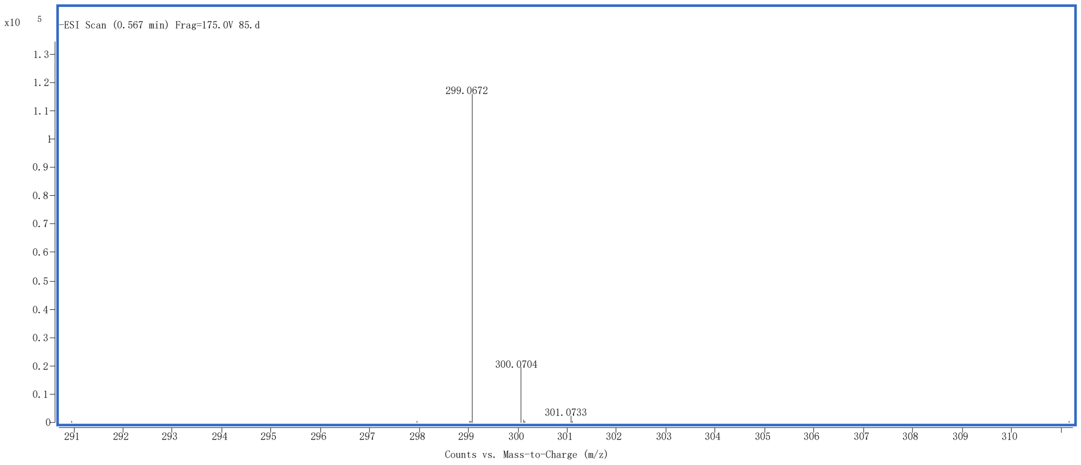
x10 5
-ESI Scan (0.567 min) Frag=175.0V 85.d
299.0672
300.0704
301.0733
Counts vs. Mass-to-Charge (m/z)

25

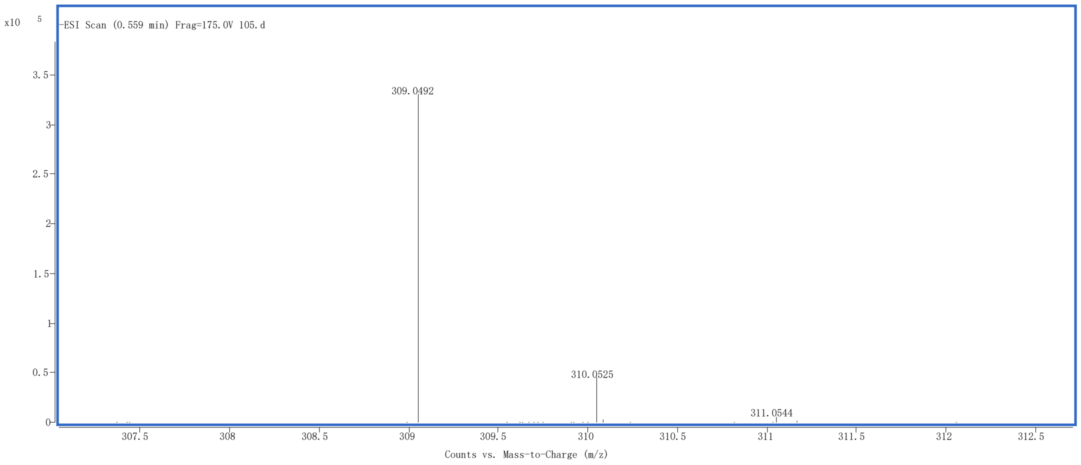
x10 5
ESI Scan (0.559 min) Frag=175.0V 105.d
309.0492
310.0525
311.0544
Counts vs. Mass-to-Charge (m/z)

26

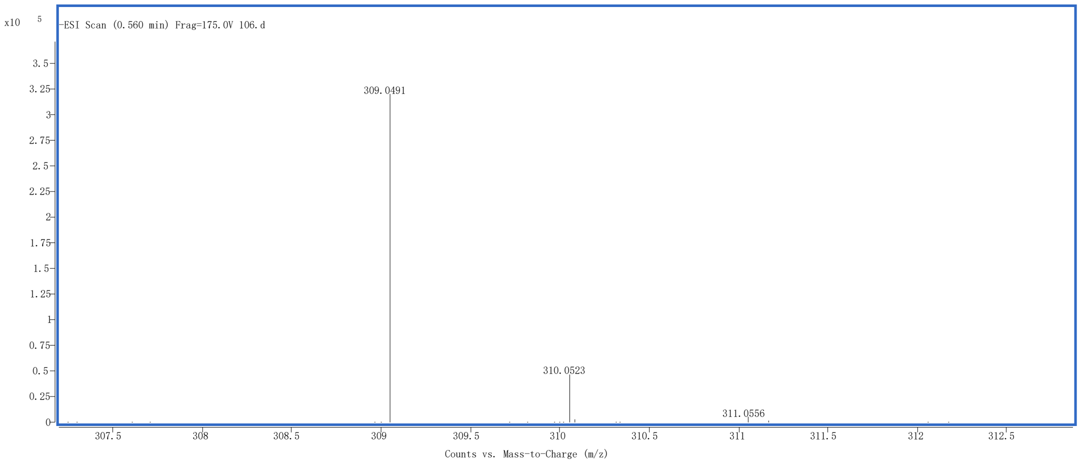
x10 5
ESI Scan (0.560 min) Frag=175.0V 106.d
309.0491
310.0523
311.0556
Counts vs. Mass-to-Charge (m/z)

27

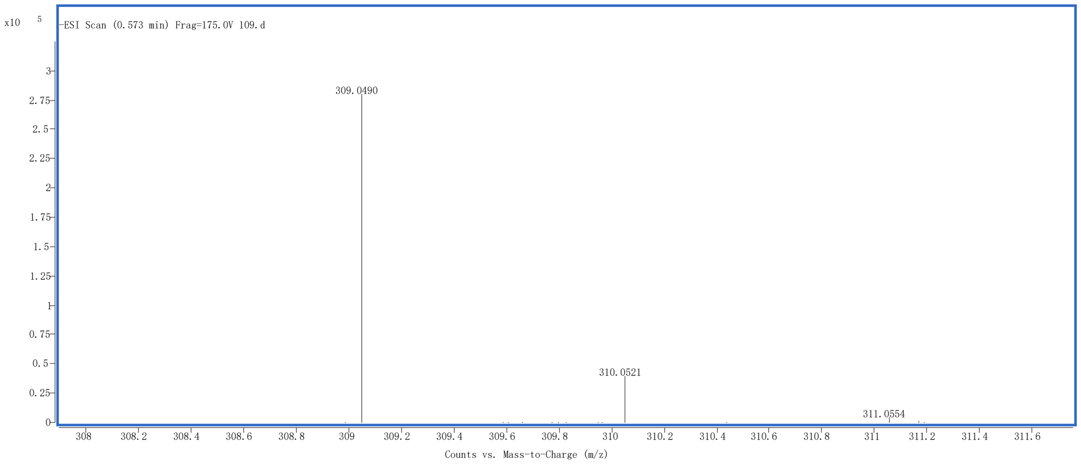
x10 5
ESI Scan (0.573 min) Frag=175.0V 109.d
309.0490
310.0521
311.0554
Counts vs. Mass-to-Charge (m/z)

28

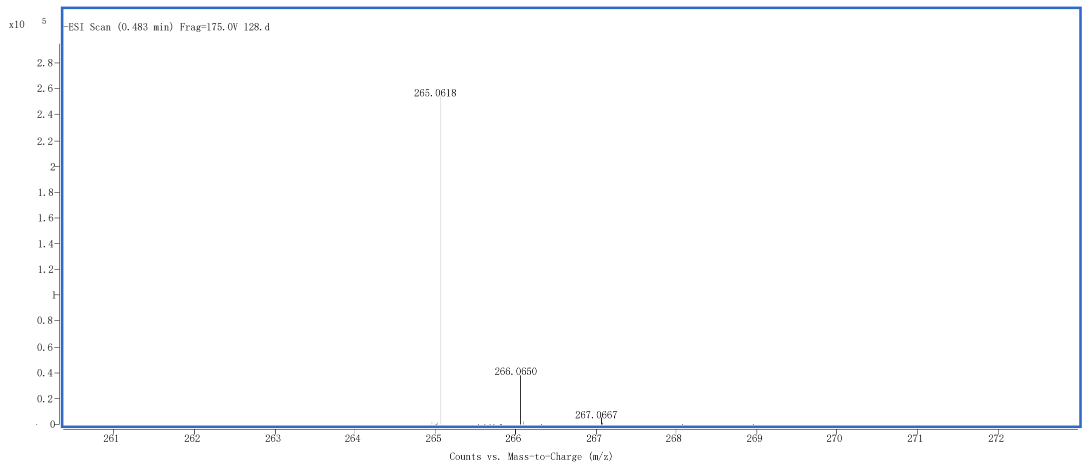
x10 5
ESI Scan (0.483 min) Frag=175.0V 128.d
265.0618
266.0650
267.0667
Counts vs. Mass-to-Charge (m/z)

29

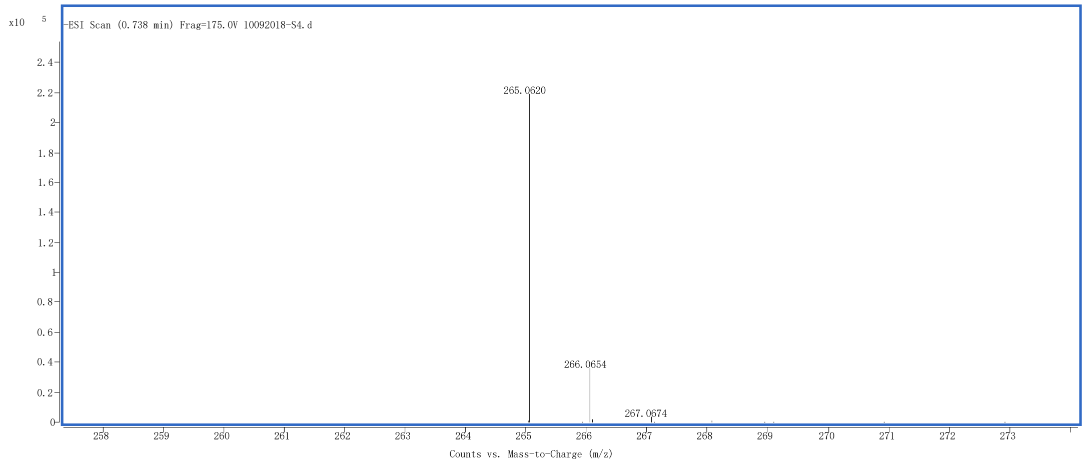
x10 5
ESI Scan (0.738 min) Frag=175.0V 10092018-S4.d
265.0620
266.0654
267.0674
Counts vs. Mass-to-Charge (m/z)

30

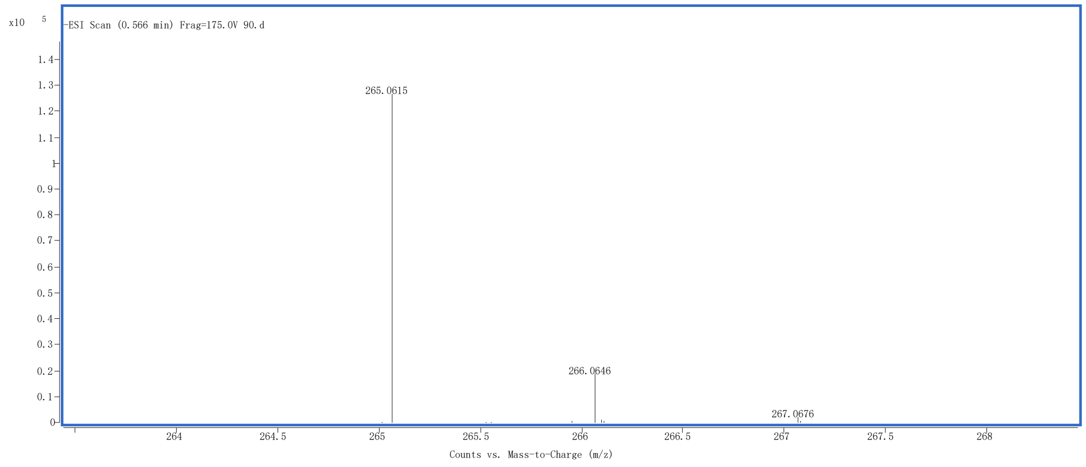
x10 5
ESI Scan (0.566 min) Frag=175.0V 90.d
265.0615
266.0646
267.0676
Counts vs. Mass-to-Charge (m/z)

31

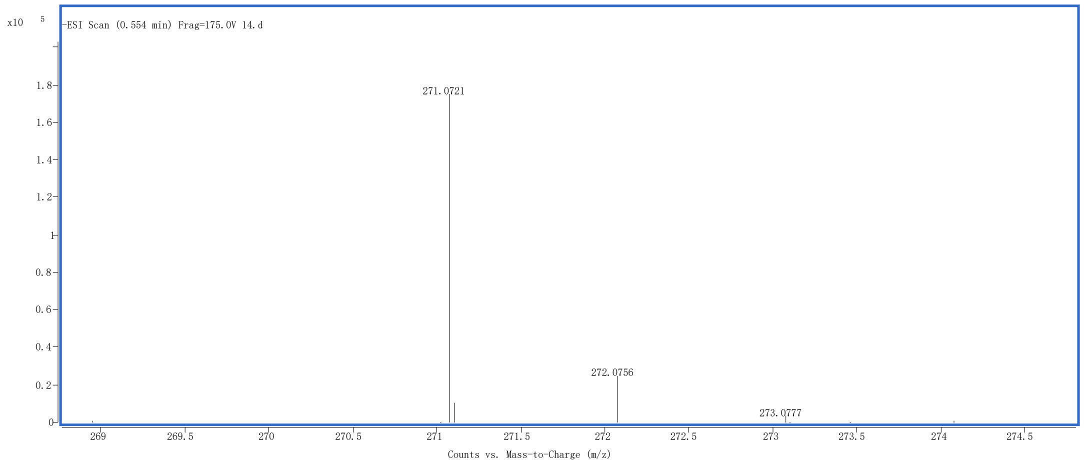
x10 5
-ESI Scan (0.554 min) Frag=175.0V 14.d
271.0721
272.0756
273.0777
Counts vs. Mass-to-Charge (m/z)

32

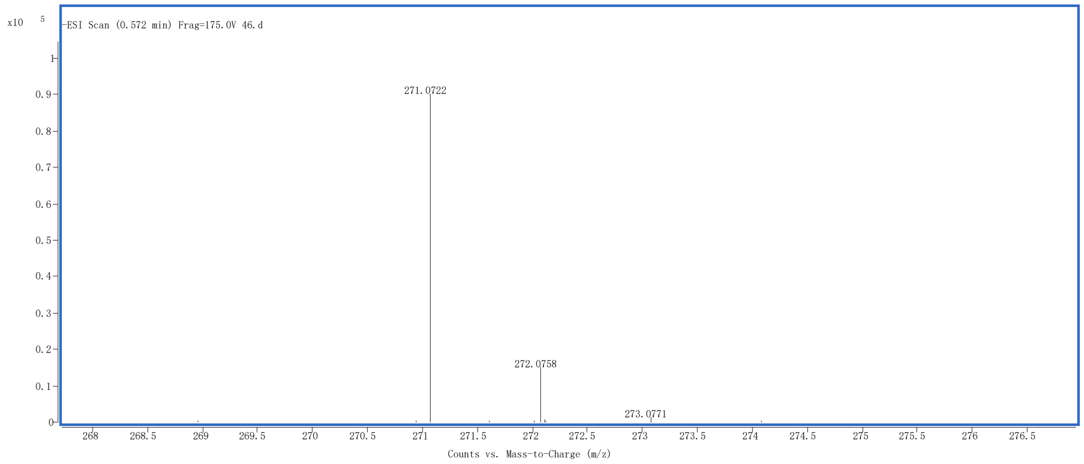
x10 5
-ESI Scan (0.572 min) Frag=175.0V 46.d
271.0722
272.0758
273.0771
Counts vs. Mass-to-Charge (m/z)

33

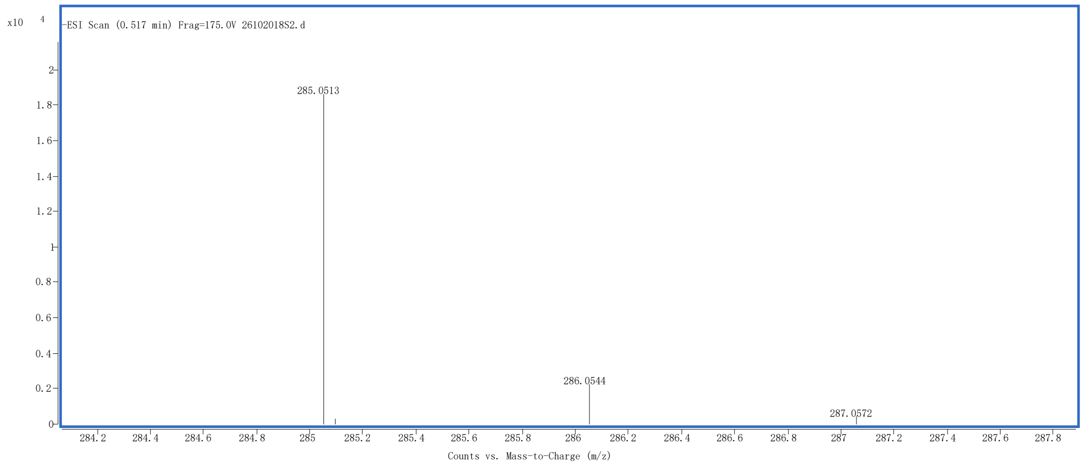
x10 4
-ESI Scan (0.517 min) Frag=175.0V 26102018S2.d
285.0513
286.0544
287.0572
Counts vs. Mass-to-Charge (m/z)

34

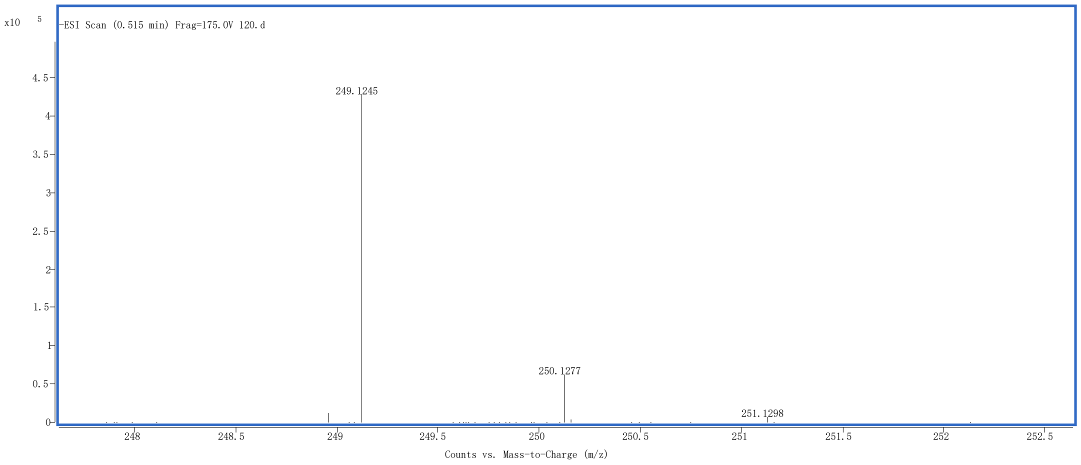
x10 5
ESI Scan (0.515 min) Frag=175.0V 120.d
249.1245
250.1277
251.1298
Counts vs. Mass-to-Charge (m/z)

35

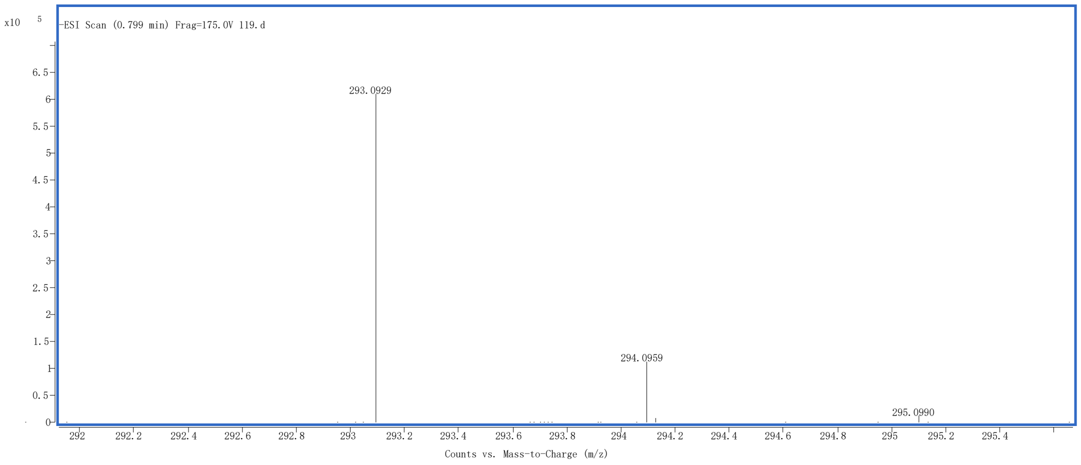
x10 5
ESI Scan (0.799 min) Frag=175.0V 119.d
293.0929
294.0959
295.0990
Counts vs. Mass-to-Charge (m/z)

36

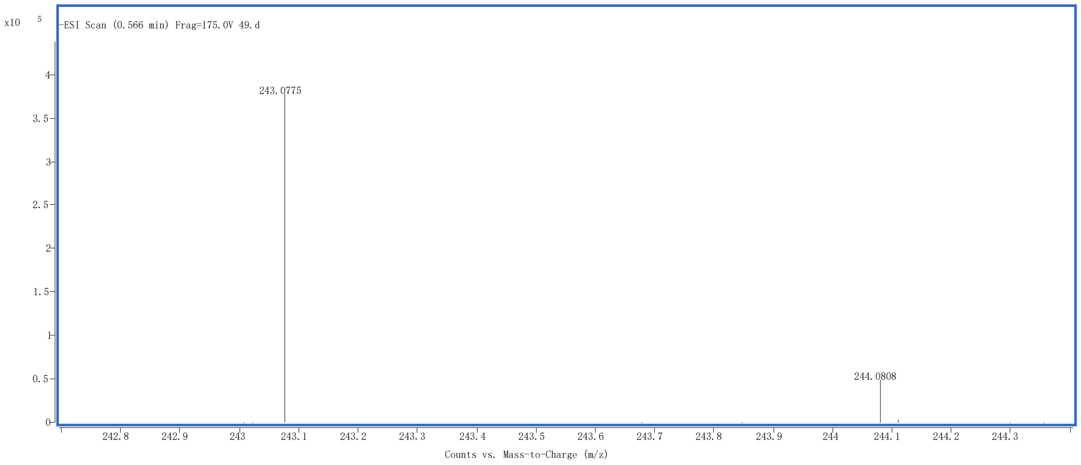
x10 5
ESI Scan (0.566 min) Frag=175.0V 49.d
243.0775
244.0808
Counts vs. Mass-to-Charge (m/z)

37

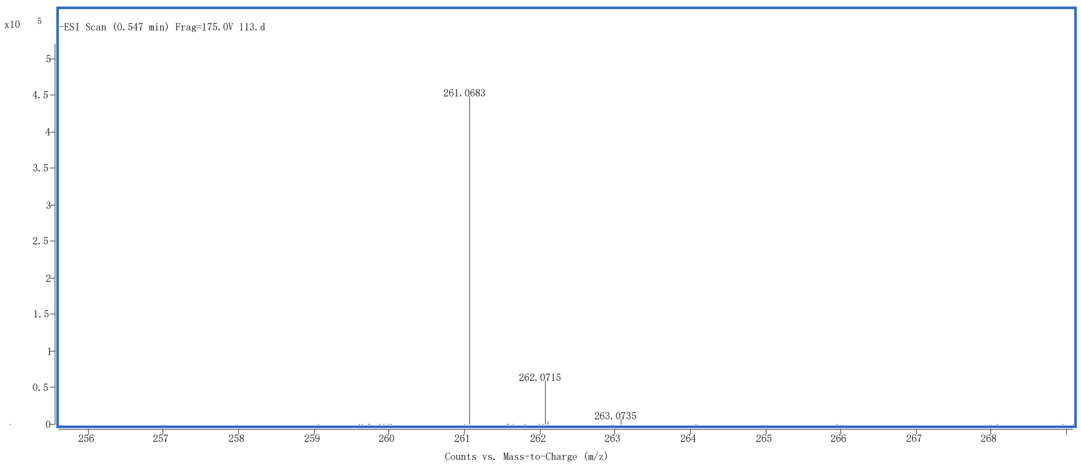
x10 5
-ESI Scan (0.547 min) Frag=175.0V 113.d
261.0683
262.0715
263.0735
Counts vs. Mass-to-Charge (m/z)

38

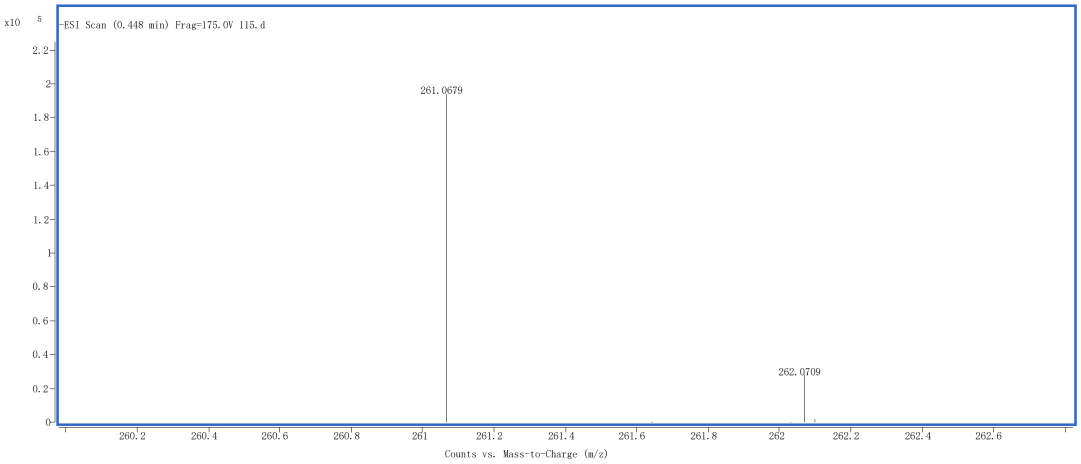
x10 5
-ESI Scan (0.448 min) Frag=175.0V 115.d
261.0679
262.0709
Counts vs. Mass-to-Charge (m/z)

39

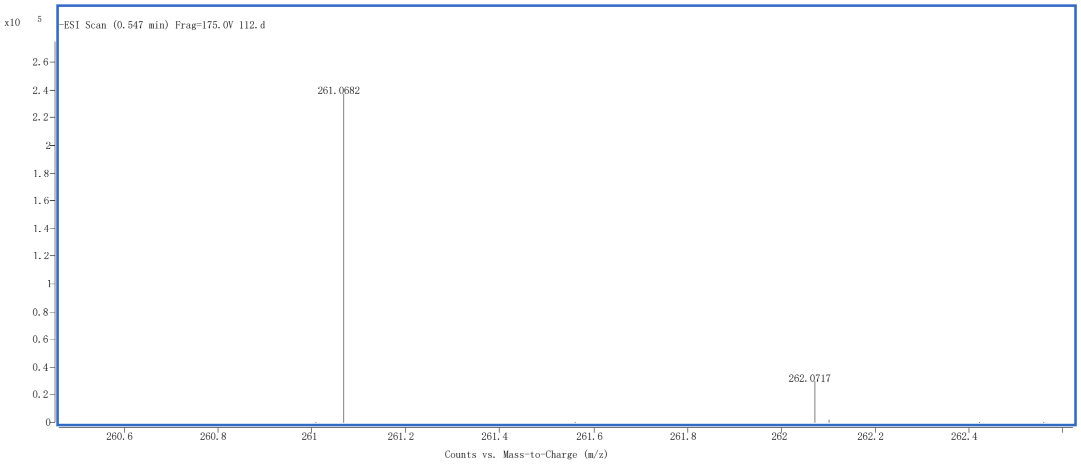
x10 5
-ESI Scan (0.547 min) Frag=175.0V 112.d
261.0682
262.0717
Counts vs. Mass-to-Charge (m/z)

40

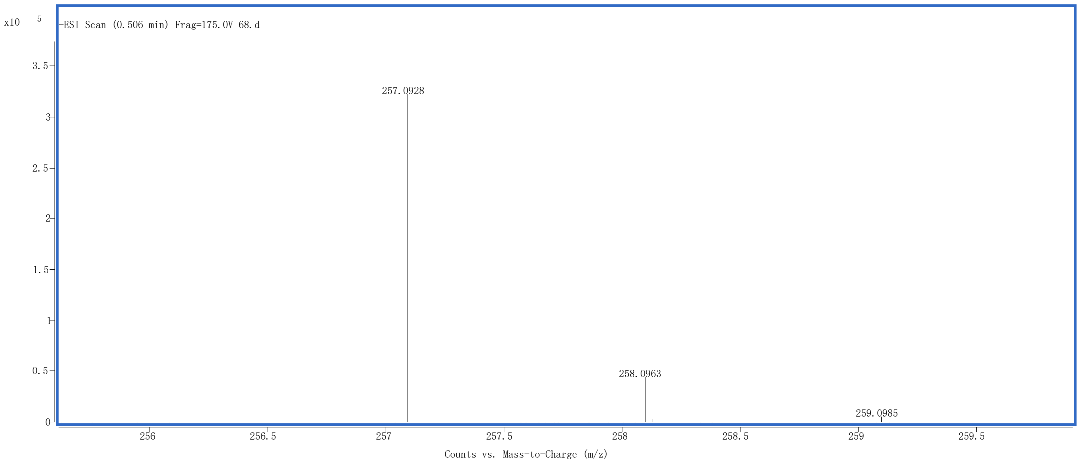
x10 5
ESI Scan (0.506 min) Frag=175.0V 68.d
257.0928
258.0963
259.0985
Counts vs. Mass-to-Charge (m/z)

41

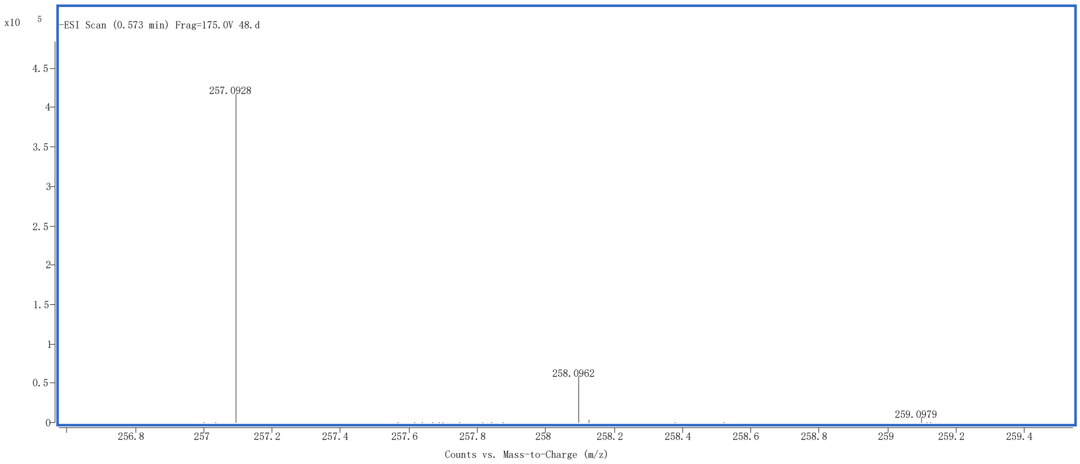
x10 5
ESI Scan (0.573 min) Frag=175.0V 48.d
257.0928
258.0962
259.0979
Counts vs. Mass-to-Charge (m/z)

42

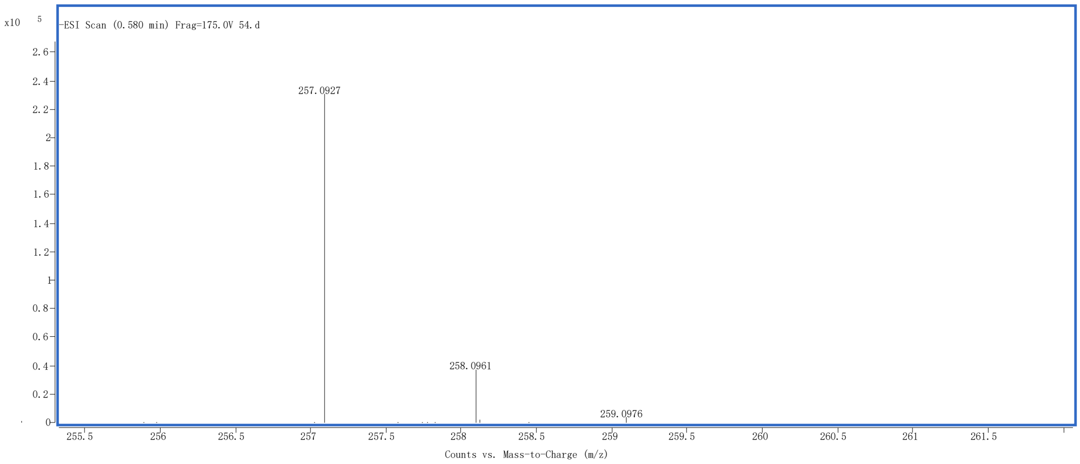
x10 5
ESI Scan (0.580 min) Frag=175.0V 54.d
257.0927
258.0961
259.0976
Counts vs. Mass-to-Charge (m/z)

43

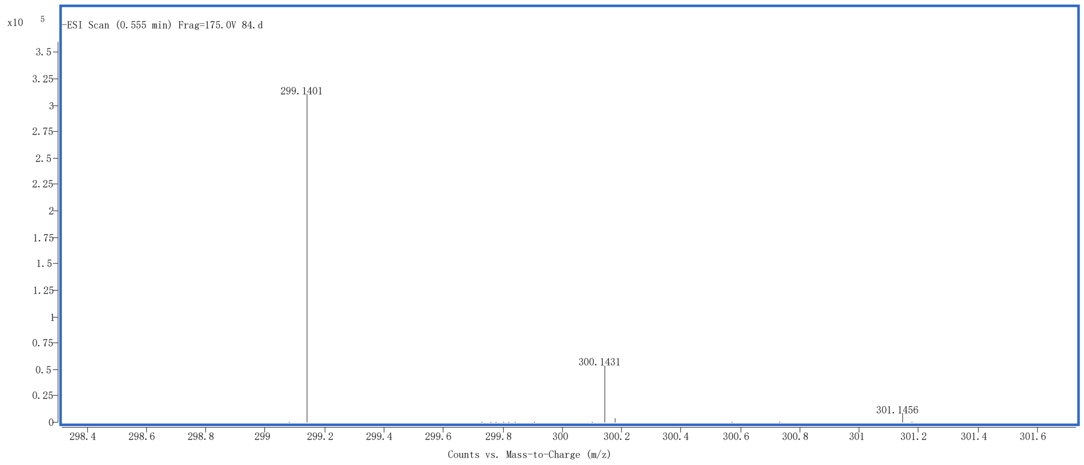
x10 5
-ESI Scan (0.555 min) Frag=175.0V 84.d
299.1401
300.1431
301.1456
Counts vs. Mass-to-Charge (m/z)

44

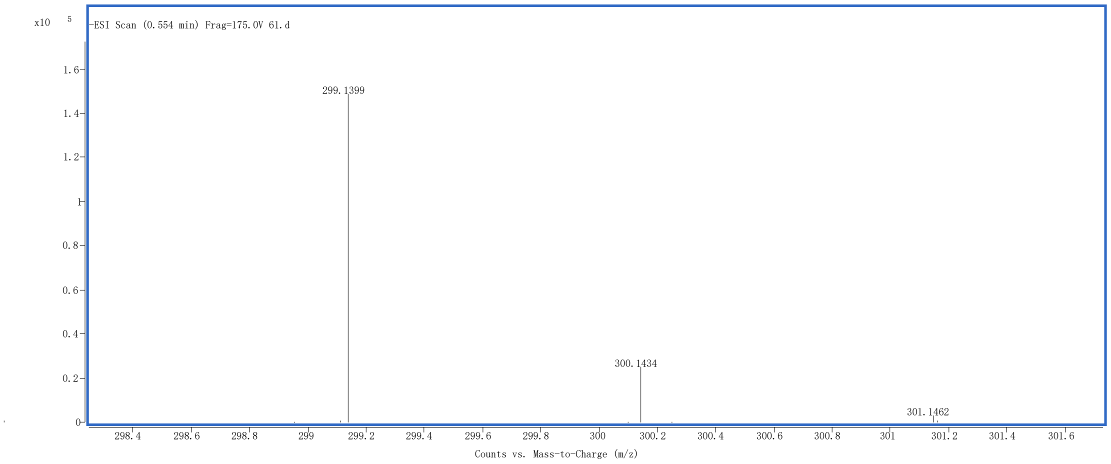
x10 5
-ESI Scan (0.554 min) Frag=175.0V 61.d
299.1399
300.1434
301.1462
Counts vs. Mass-to-Charge (m/z)

45

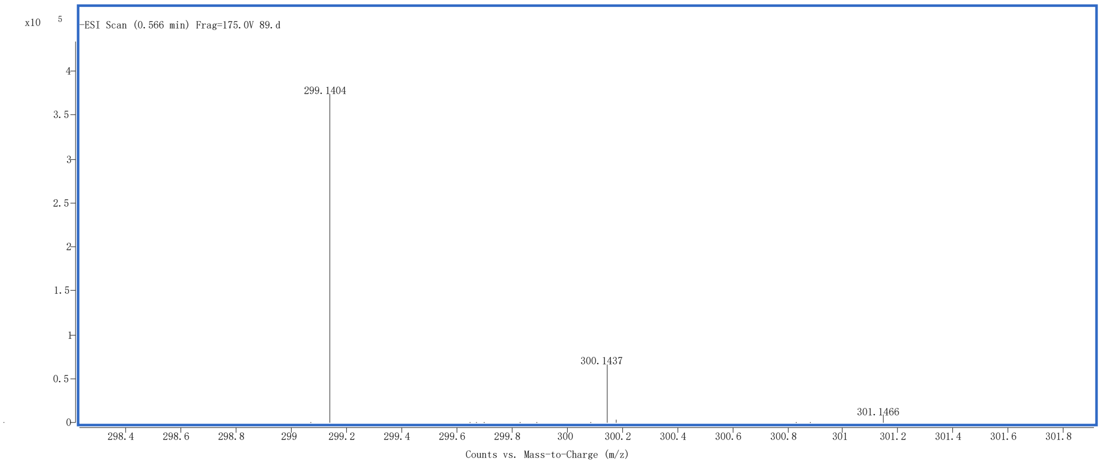
x10 5
-ESI Scan (0.566 min) Frag=175.0V 89.d
299.1404
300.1437
301.1466
Counts vs. Mass-to-Charge (m/z)

46

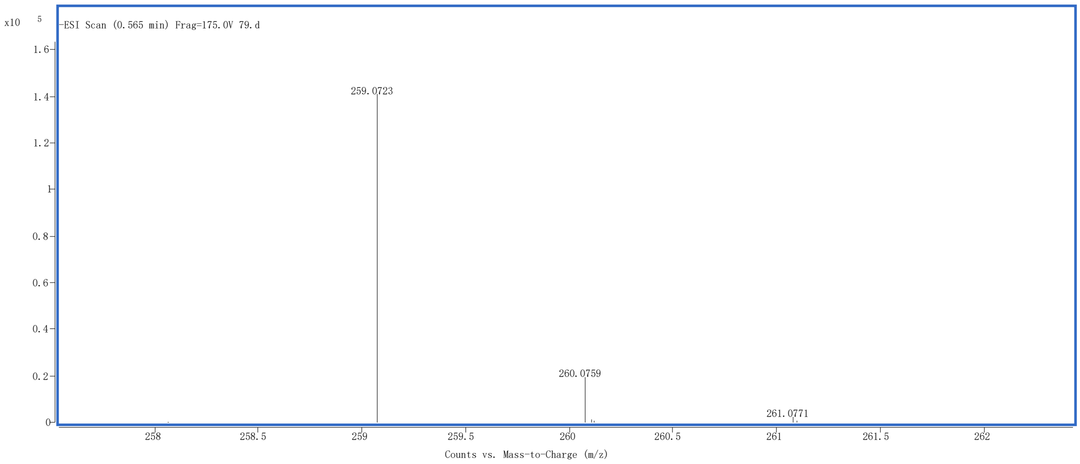

x10 5
ESI Scan (0.565 min) Frag=175.0V 79.d
259.0723
260.0759
261.0771
Counts vs. Mass-to-Charge (m/z)

47

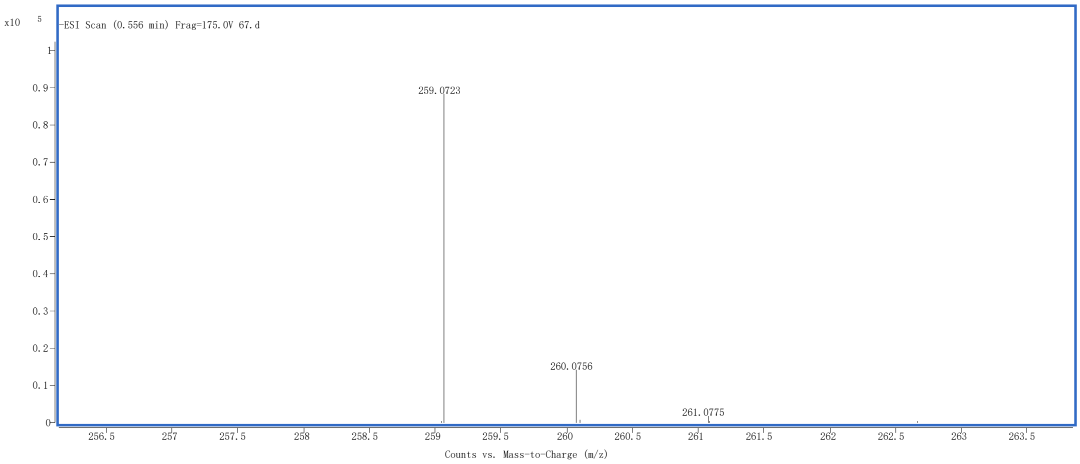

x10 5
ESI Scan (0.556 min) Frag=175.0V 67.d
259.0723
260.0756
261.0775
Counts vs. Mass-to-Charge (m/z)

48

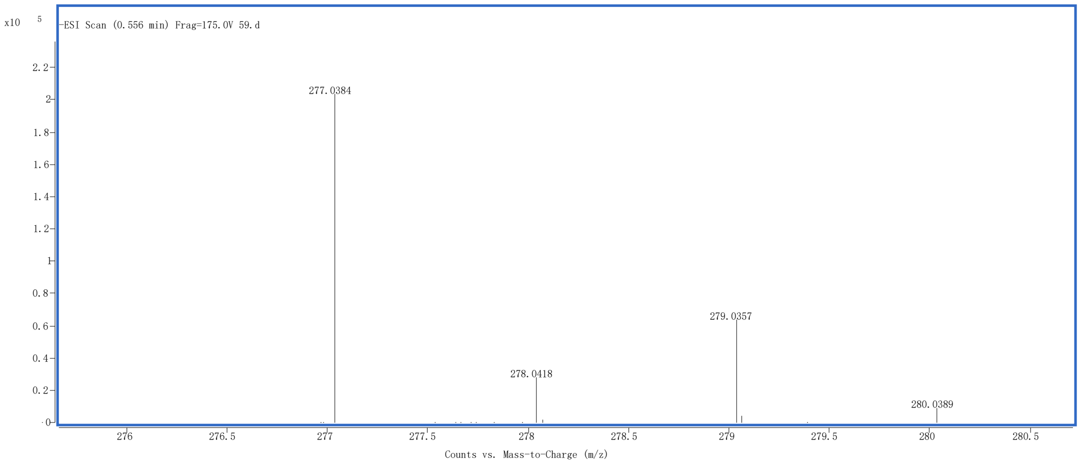

x10 5
ESI Scan (0.556 min) Frag=175.0V 59.d
277.0384
278.0418
279.0357
280.0389
Counts vs. Mass-to-Charge (m/z)

49

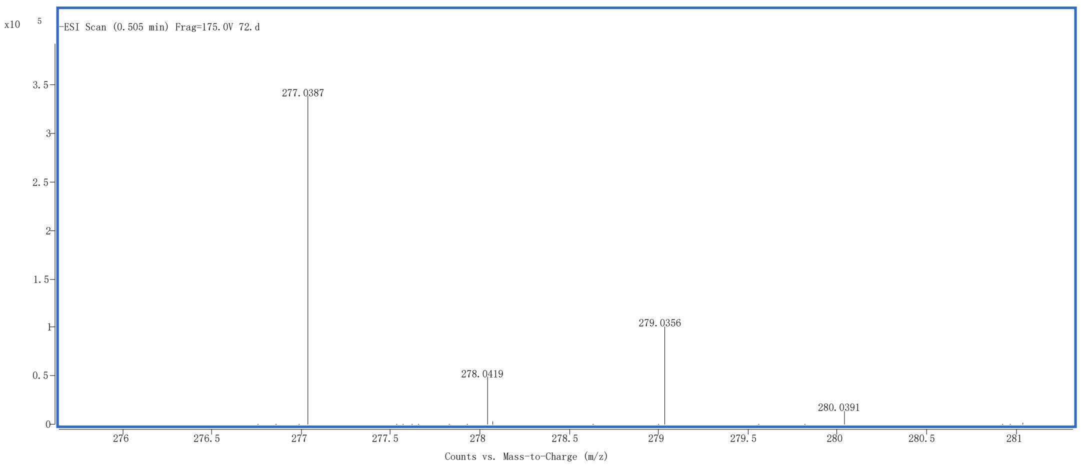
x10 5
ESI Scan (0.505 min) Frag=175.0V 72.d
277.0387
278.0419
279.0356
280.0391
Counts vs. Mass-to-Charge (m/z)

50

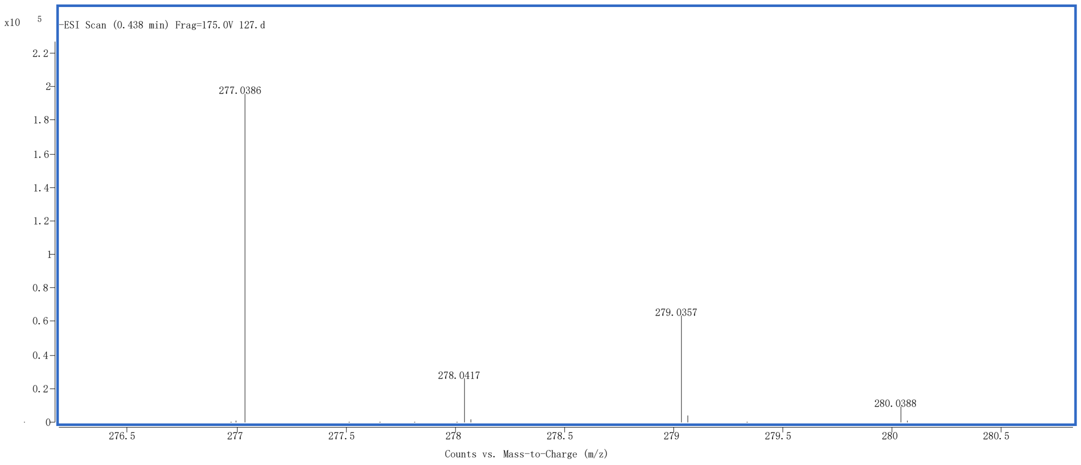
x10 5
ESI Scan (0.438 min) Frag=175.0V 127.d
277.0386
278.0417
279.0357
280.0388
Counts vs. Mass-to-Charge (m/z)

51

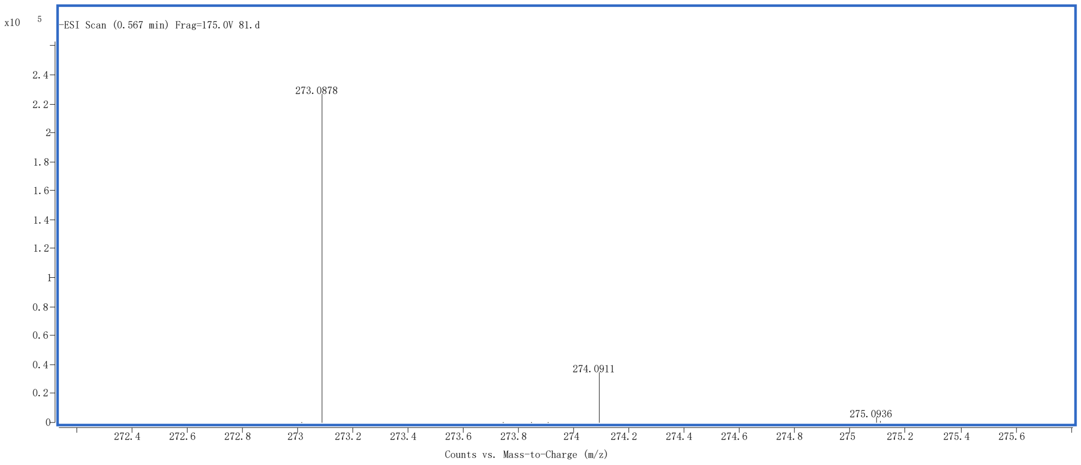
x10 5
ESI Scan (0.567 min) Frag=175.0V 81.d
273.0878
274.0911
275.0936
Counts vs. Mass-to-Charge (m/z)

52

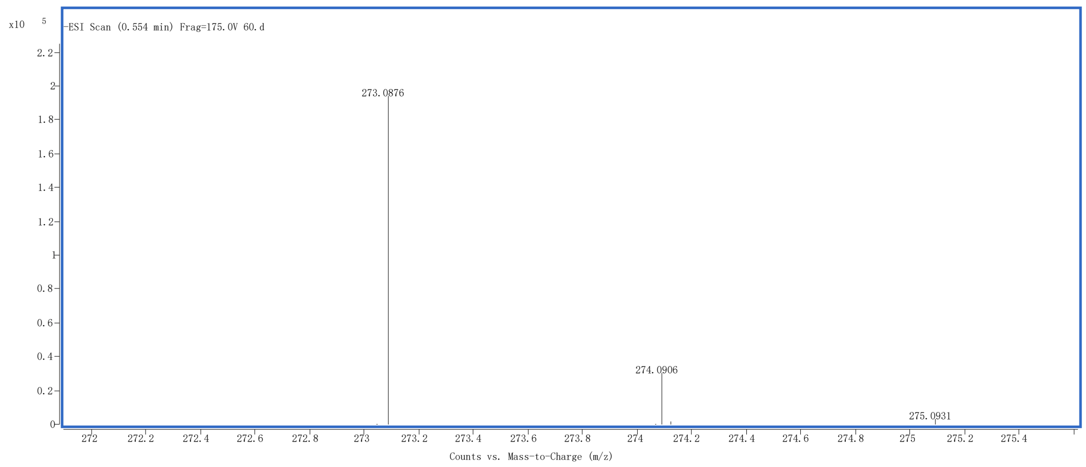
x10 5
-ESI Scan (0.554 min) Frag=175.0V 60.d
273.0876
274.0906
275.0931
Counts vs. Mass-to-Charge (m/z)

53

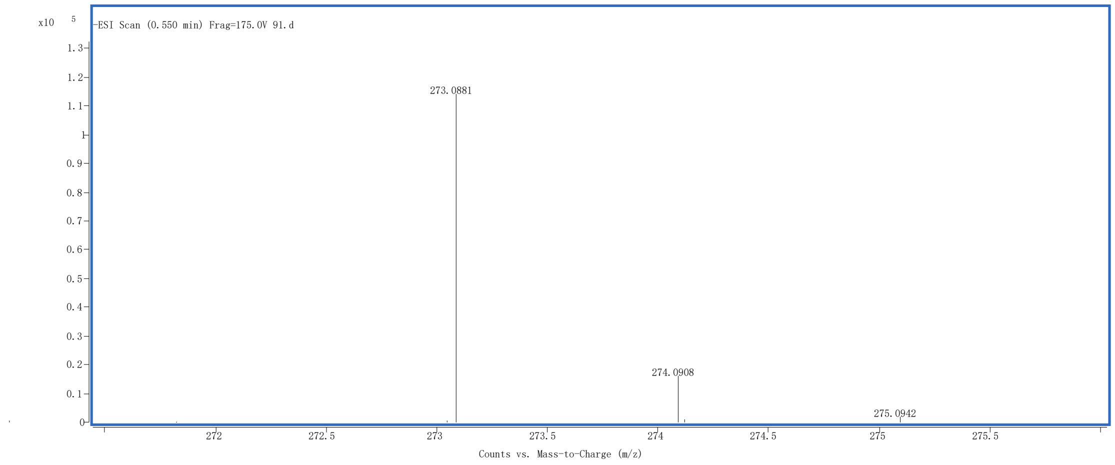
x10 5
-ESI Scan (0.550 min) Frag=175.0V 91.d
273.0881
274.0908
275.0942
Counts vs. Mass-to-Charge (m/z)

54

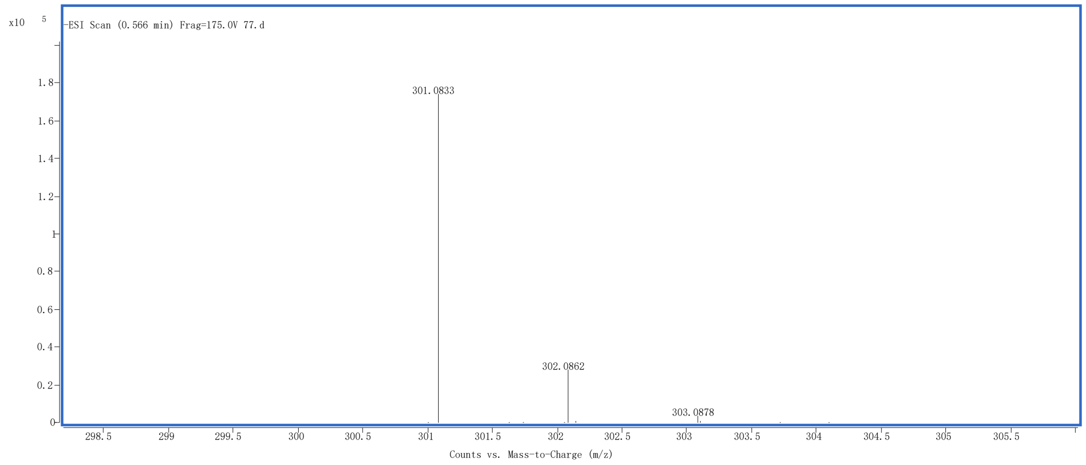
x10 5
-ESI Scan (0.566 min) Frag=175.0V 77.d
301.0833
302.0862
303.0878
Counts vs. Mass-to-Charge (m/z)

55

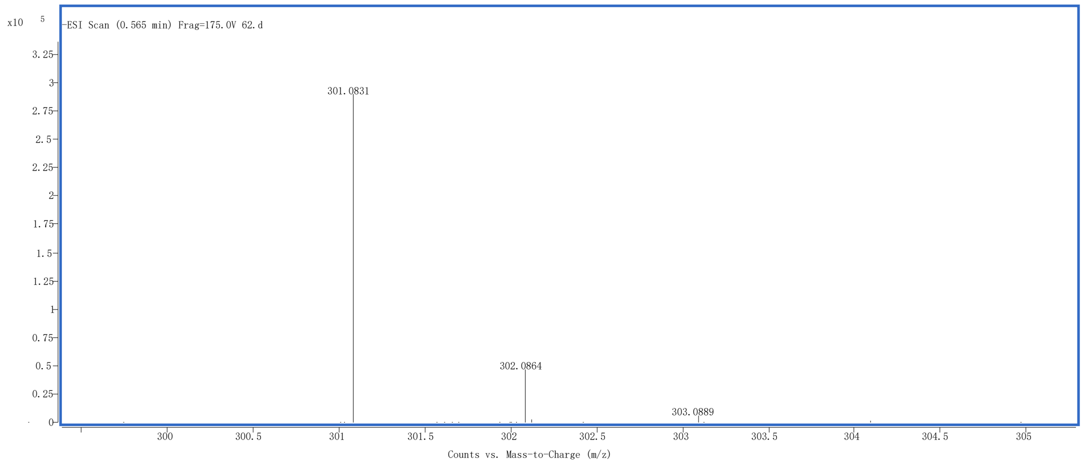
x10 5
ESI Scan (0.565 min) Frag=175.0V 62.d
301.0831
302.0864
303.0889
Counts vs. Mass-to-Charge (m/z)

56

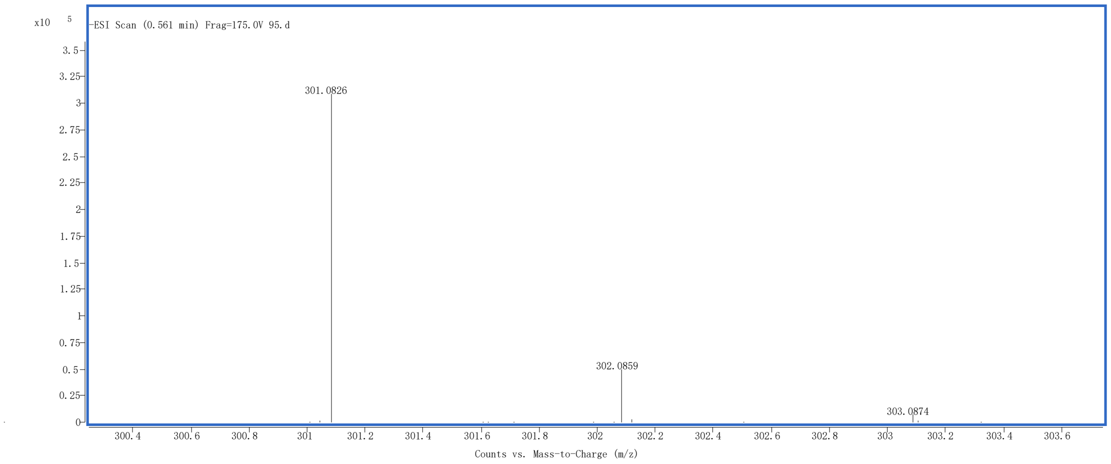
x10 5
ESI Scan (0.561 min) Frag=175.0V 95.d
301.0826
302.0859
303.0874
Counts vs. Mass-to-Charge (m/z)

57

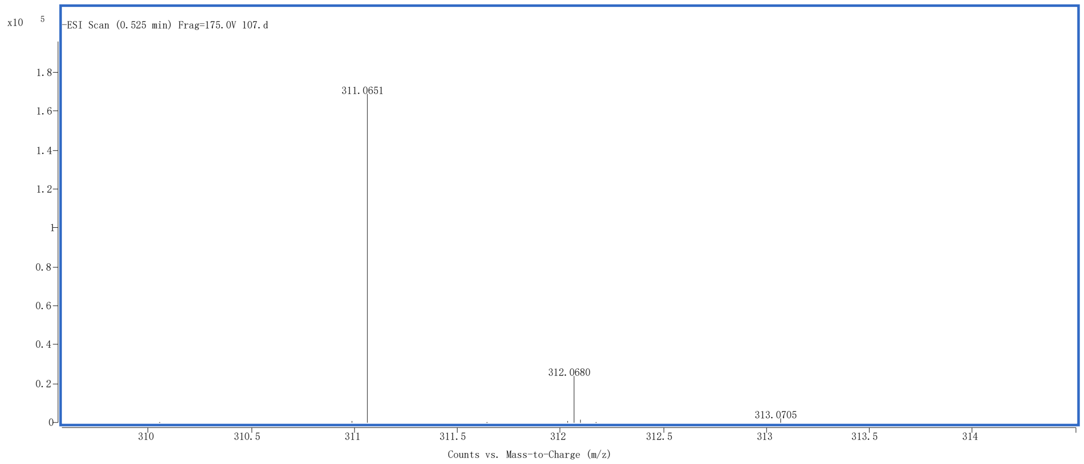
x10 5
ESI Scan (0.525 min) Frag=175.0V 107.d
311.0651
312.0680
313.0705
Counts vs. Mass-to-Charge (m/z)

58

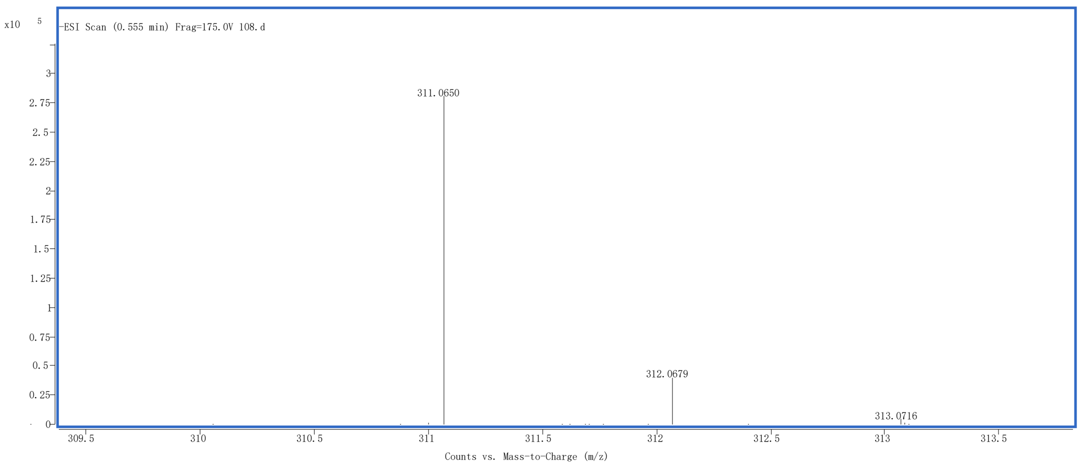
x10 5
ESI Scan (0.555 min) Frag=175.0V 108.d
311.0650
312.0679
313.0716
Counts vs. Mass-to-Charge (m/z)

59

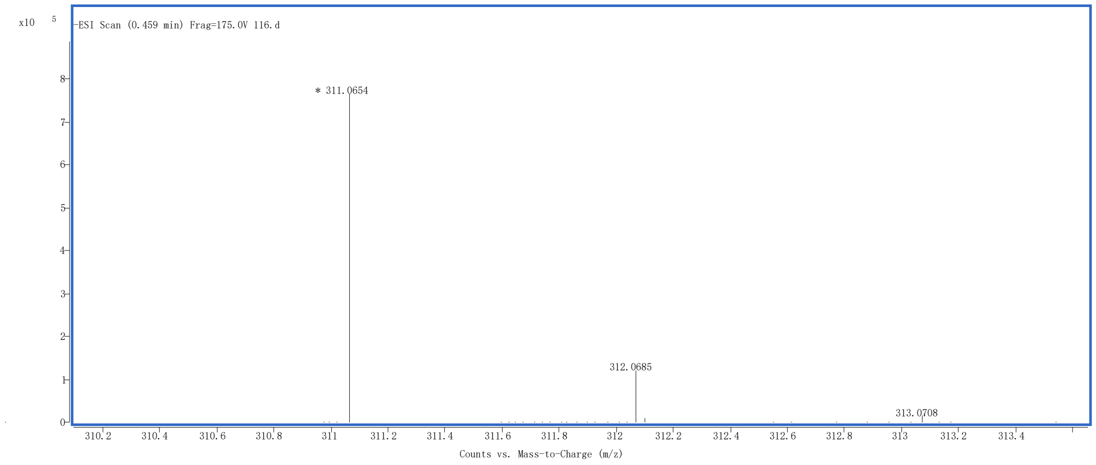
x10 5
ESI Scan (0.459 min) Frag=175.0V 116.d
* 311.0654
312.0685
313.0708
Counts vs. Mass-to-Charge (m/z)

60

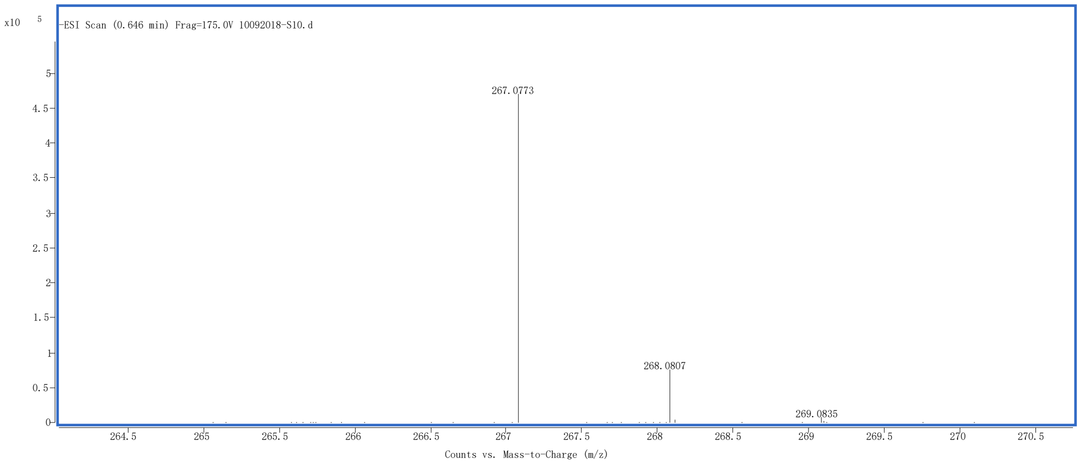
x10 5
ESI Scan (0.646 min) Frag=175.0V 10092018-S10.d
267.0773
268.0807
269.0835
Counts vs. Mass-to-Charge (m/z)

61

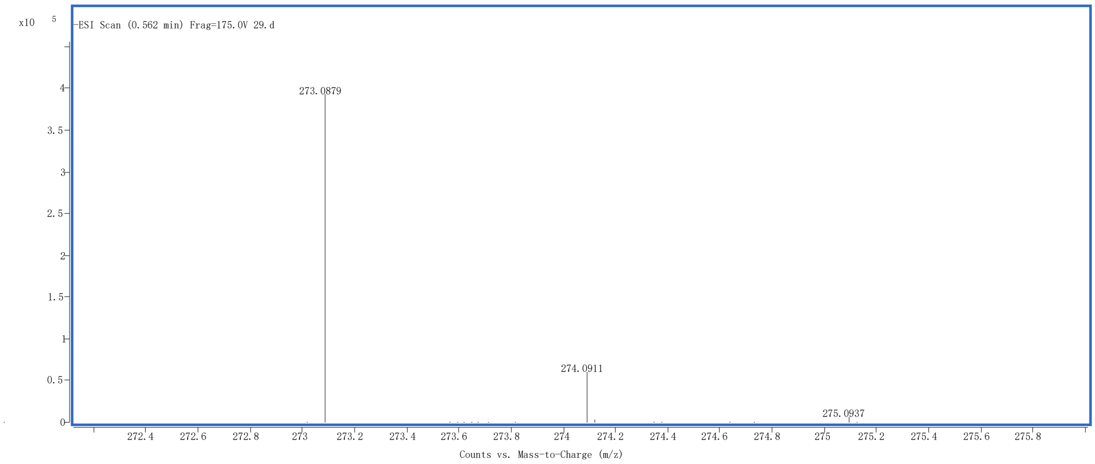
x10 5
ESI Scan (0.562 min) Frag=175.0V 29.d
273.0879
274.0911
275.0937
Counts vs. Mass-to-Charge (m/z)

62

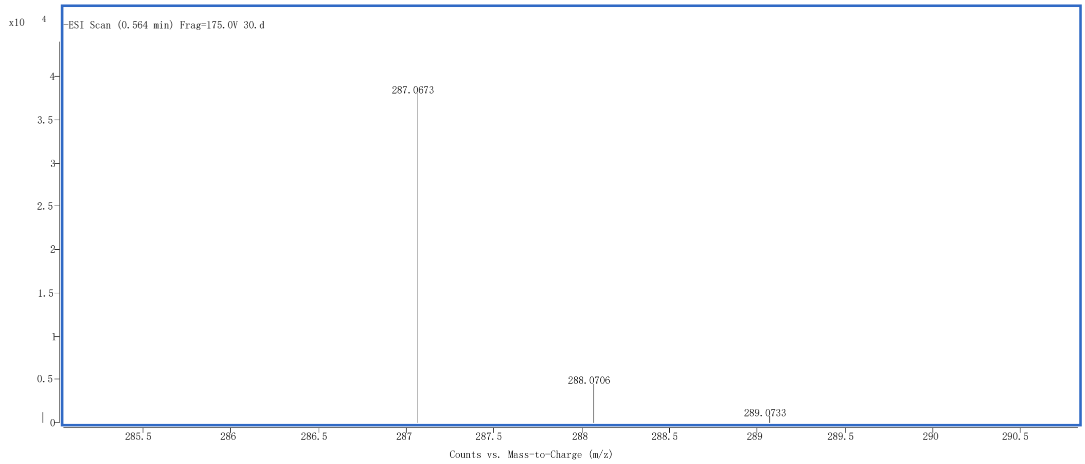
x10 4
ESI Scan (0.564 min) Frag=175.0V 30.d
287.0673
288.0706
289.0733
Counts vs. Mass-to-Charge (m/z)

63

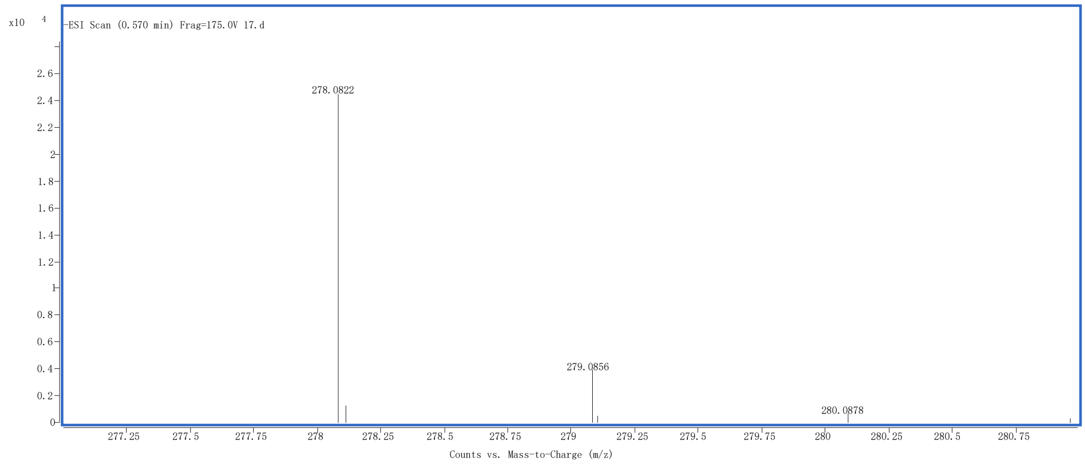
x10 4
ESI Scan (0.570 min) Frag=175.0V 17.d
278.0822
279.0856
280.0878
Counts vs. Mass-to-Charge (m/z)

64

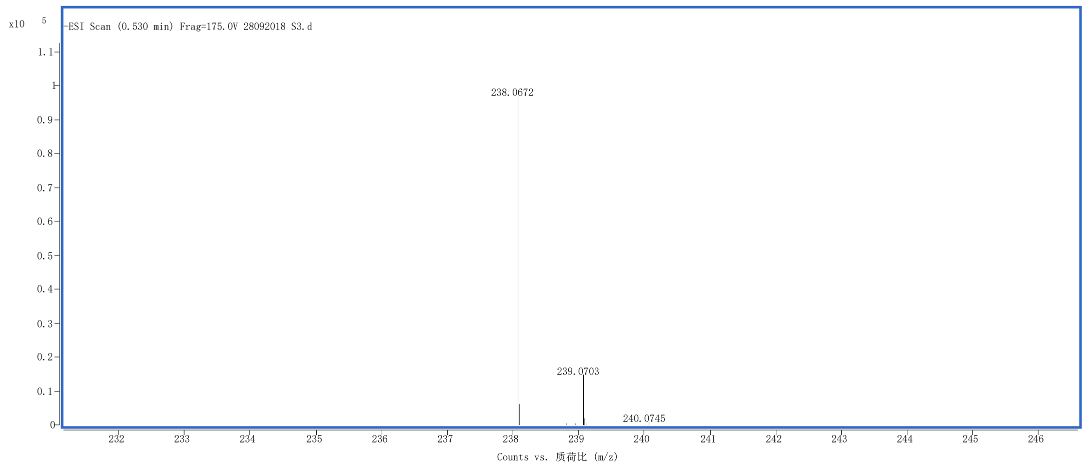
x10 5
-ESI Scan (0.530 min) Frag=175.0V 28092018 S3.d
238.0672
239.0703
240.0745
Counts vs. 质荷比 (m/z)

65

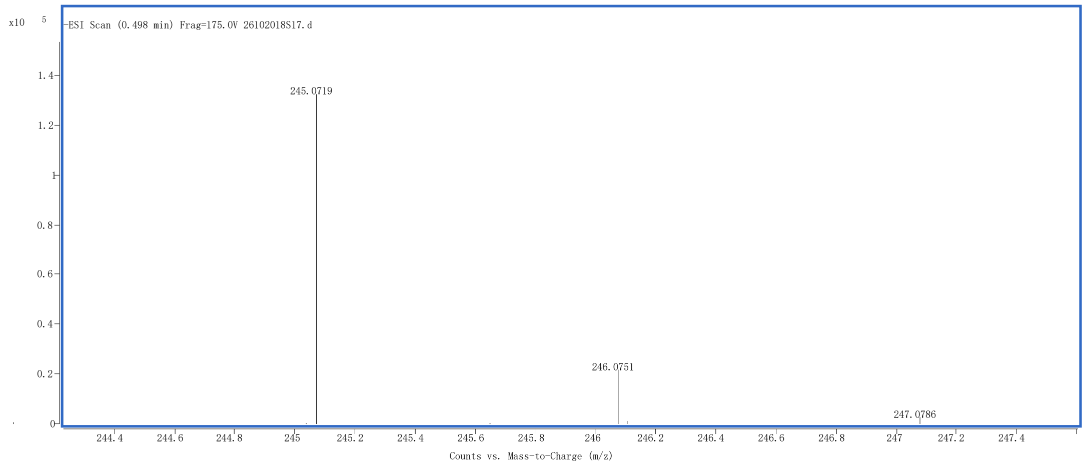
x10 5
-ESI Scan (0.498 min) Frag=175.0V 26102018S17.d
245.0719
246.0751
247.0786
Counts vs. Mass-to-Charge (m/z)

66

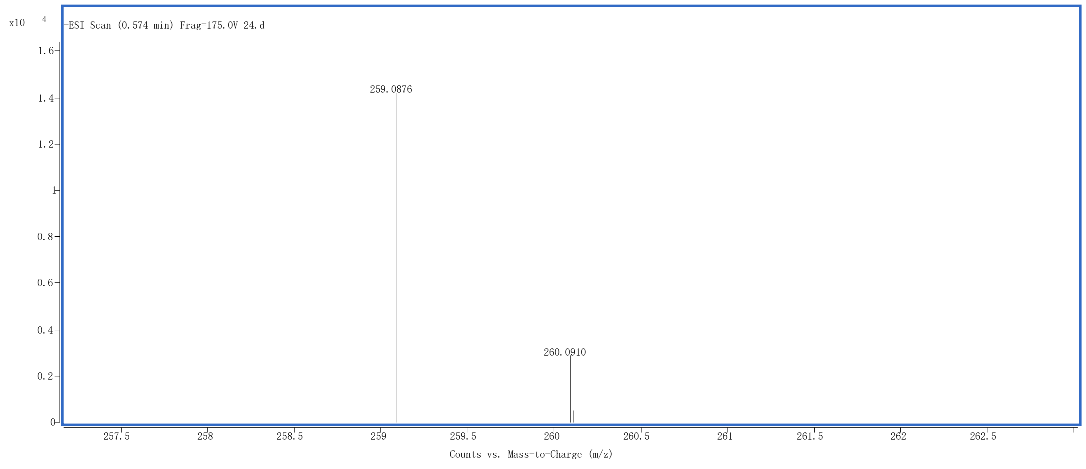
x10 4
-ESI Scan (0.574 min) Frag=175.0V 24.d
259.0876
260.0910
Counts vs. Mass-to-Charge (m/z)

67

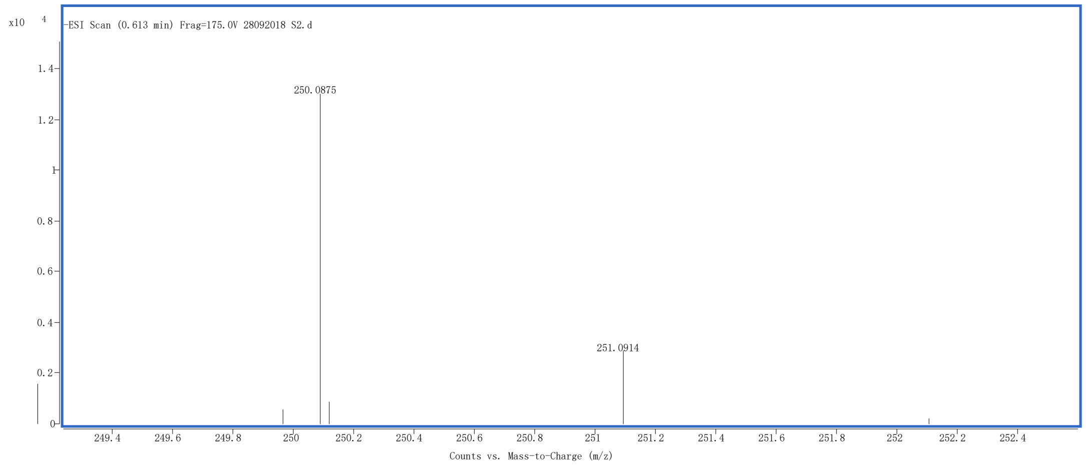
x10 4
-ESI Scan (0.613 min) Frag=175.0V 28092018 S2.d
250.0875
251.0914
Counts vs. Mass-to-Charge (m/z)

68

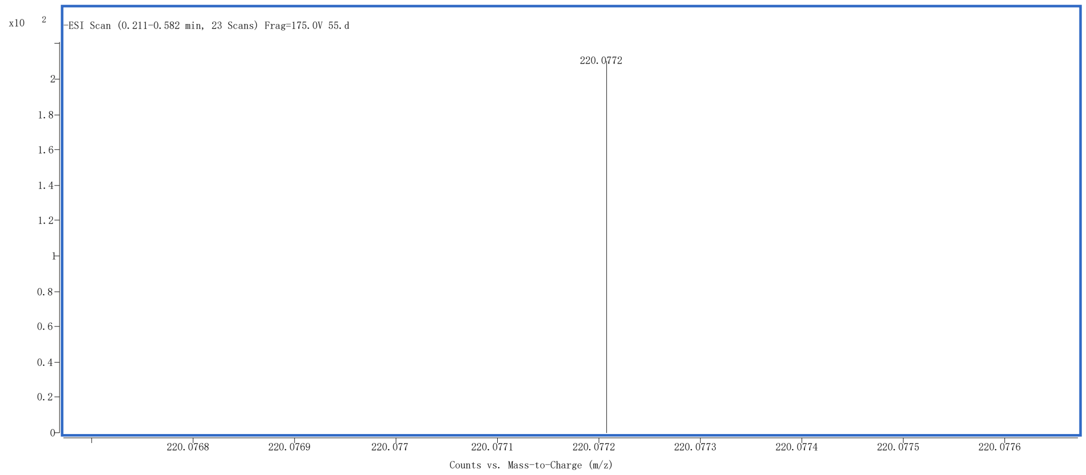
x10 2
-ESI Scan (0.211-0.582 min, 23 Scans) Frag=175.0V 55.d
220.0772
Counts vs. Mass-to-Charge (m/z)

69

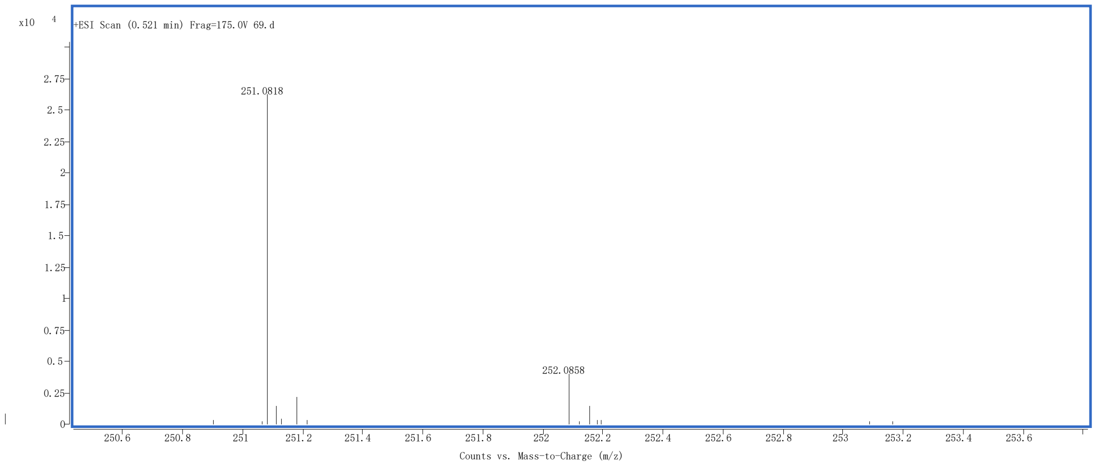
x10 4
+ESI Scan (0.521 min) Frag=175.0V 69.d
251.0818
252.0858
Counts vs. Mass-to-Charge (m/z)

70

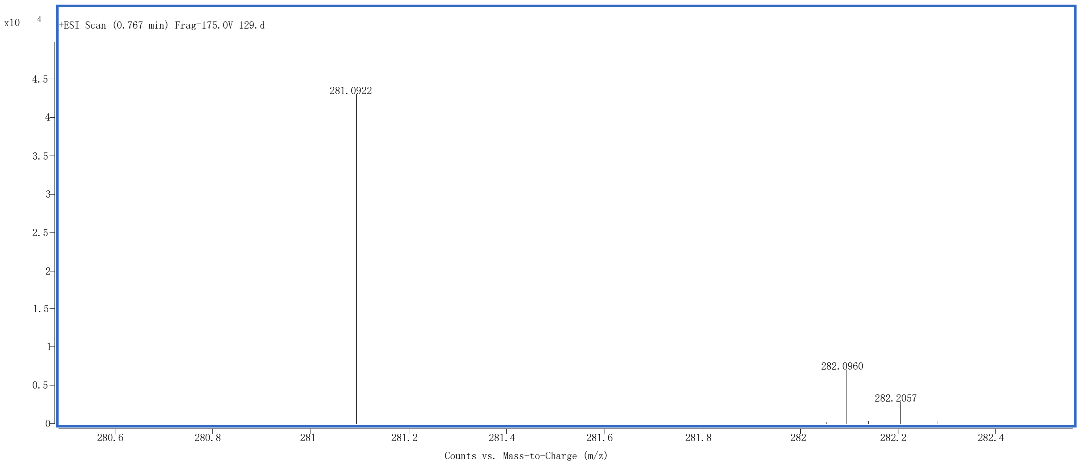
x10 4
+ESI Scan (0.767 min) Frag=175.0V 129.d
281.0922
282.0960
282.2057
Counts vs. Mass-to-Charge (m/z)

71

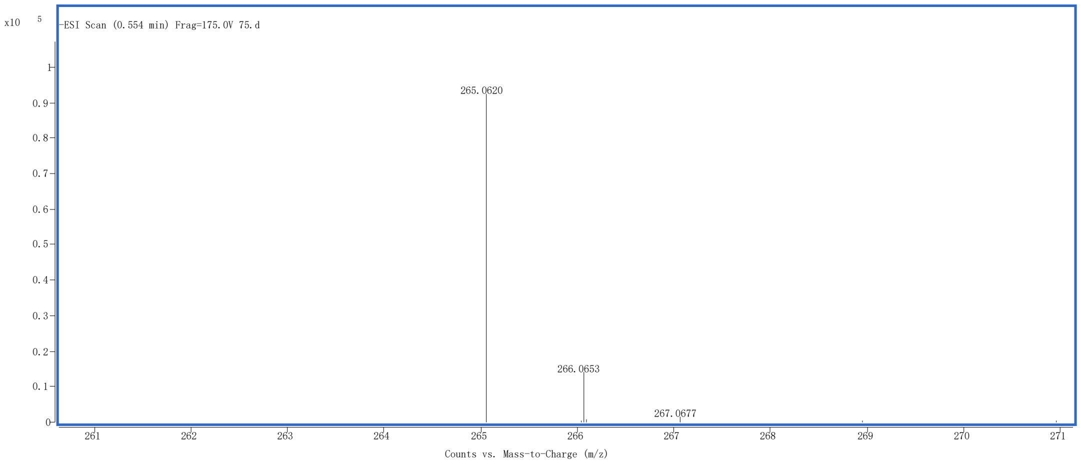
x10 5
-ESI Scan (0.554 min) Frag=175.0V 75.d
265.0620
266.0653
267.0677
Counts vs. Mass-to-Charge (m/z)

72

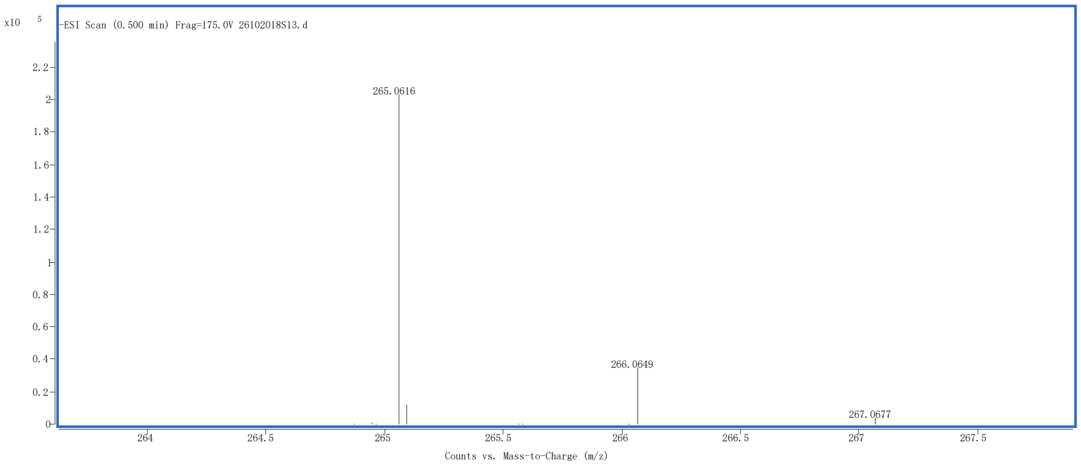
x10 5
-ESI Scan (0.500 min) Frag=175.0V 26102018S13.d
265.0616
266.0649
267.0677
Counts vs. Mass-to-Charge (m/z)

73

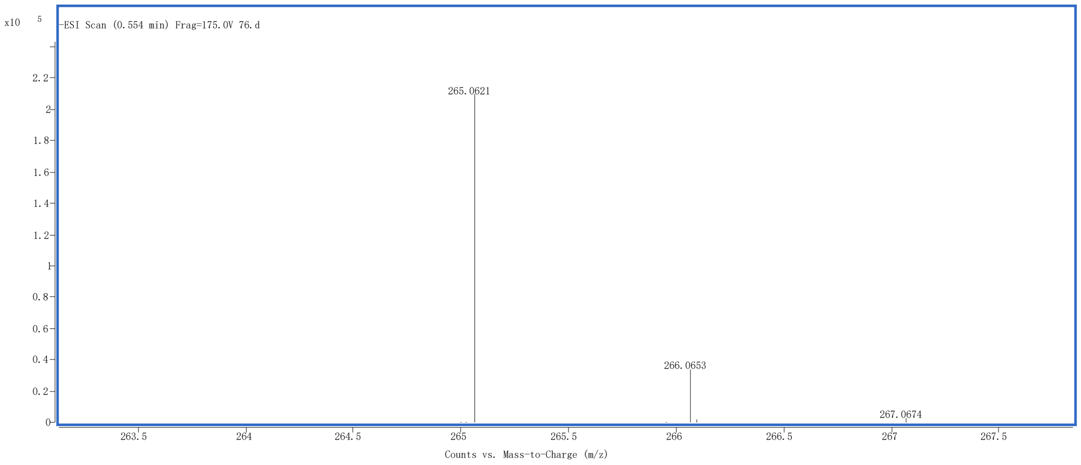
x10 5
-ESI Scan (0.554 min) Frag=175.0V 76.d
265.0621
266.0653
267.0674
Counts vs. Mass-to-Charge (m/z)

74

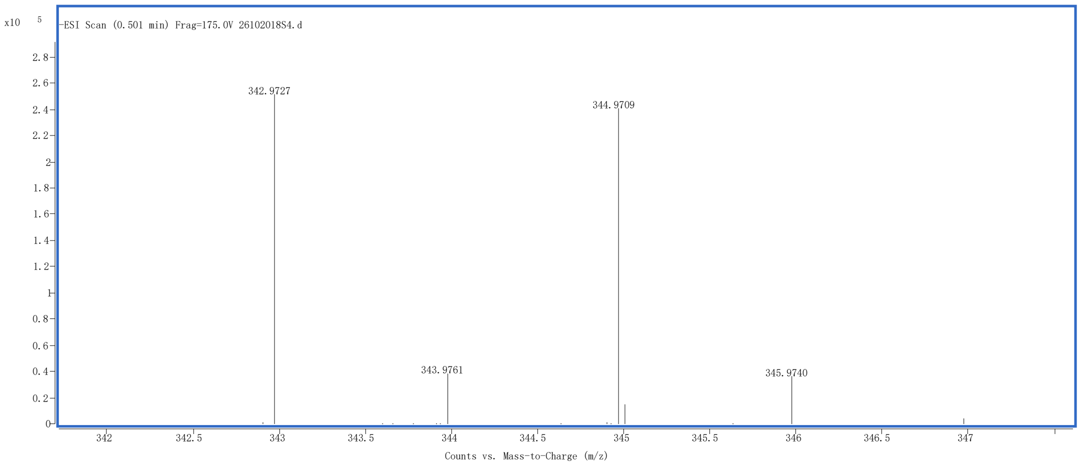
x10 5
-ESI Scan (0.501 min) Frag=175.0V 26102018S4.d
342.9727
344.9709
343.9761
345.9740
Counts vs. Mass-to-Charge (m/z)

75

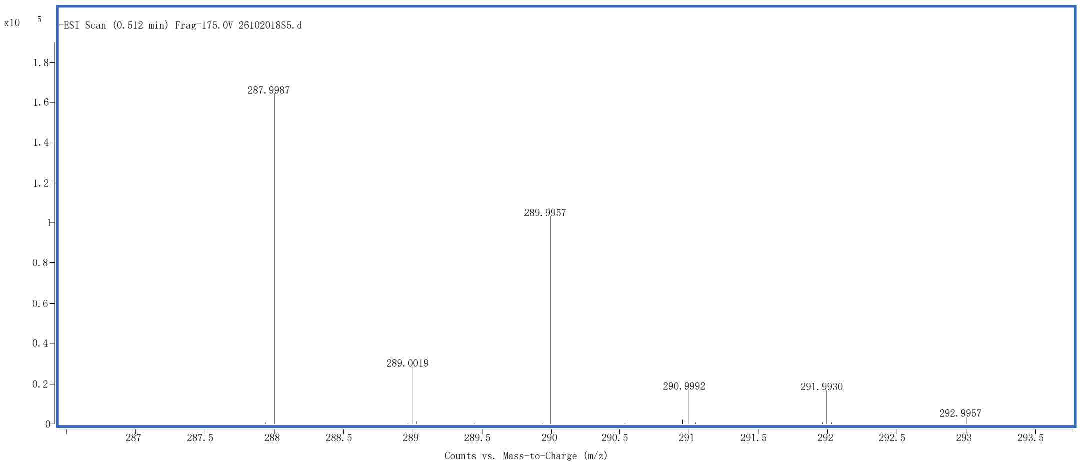
x10 5
-ESI Scan (0.512 min) Frag=175.0V 26102018S5.d
287.9987
289.9957
289.0019
290.9992
291.9930
292.9957
Counts vs. Mass-to-Charge (m/z)

76

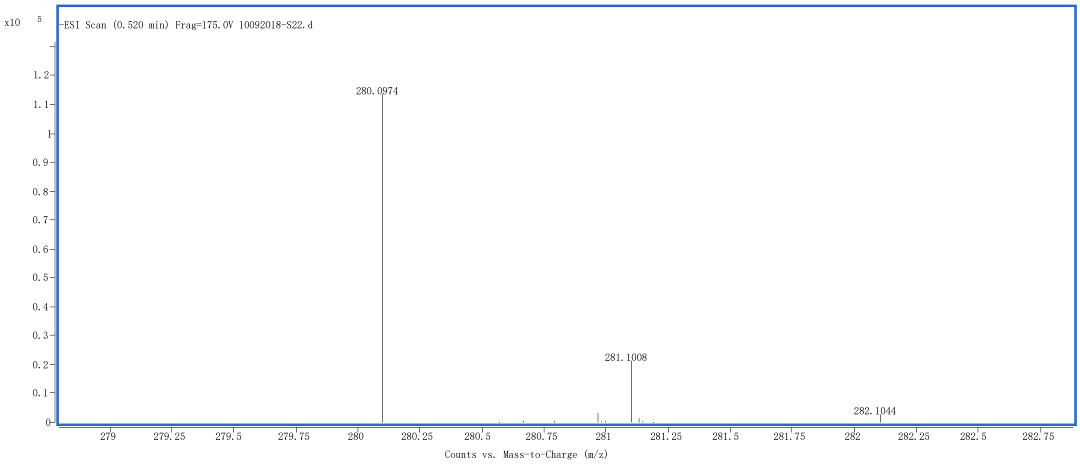
x10 5
ESI Scan (0.520 min) Frag=175.0V 10092018-S22.d
280.0974
281.1008
282.1044
Counts vs. Mass-to-Charge (m/z)

77

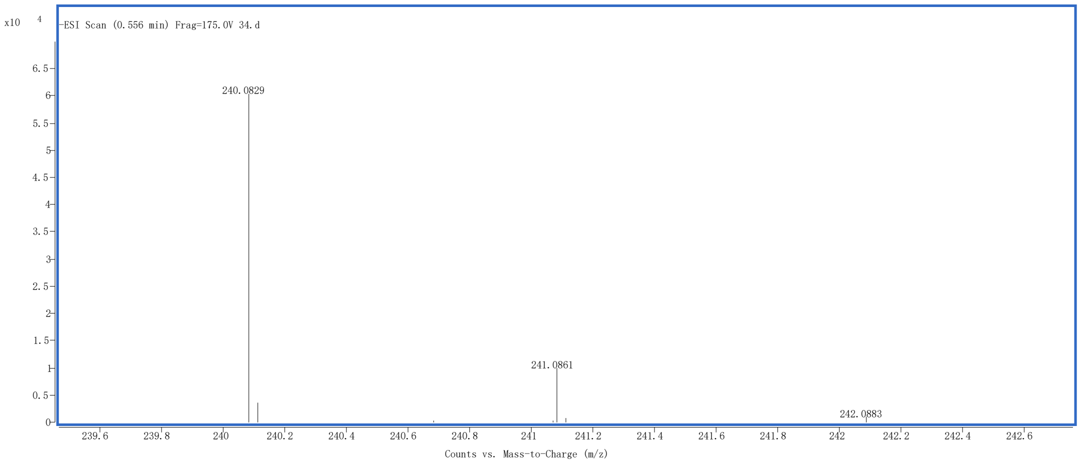
x10 4
ESI Scan (0.556 min) Frag=175.0V 34.d
240.0829
241.0861
242.0883
Counts vs. Mass-to-Charge (m/z)

78

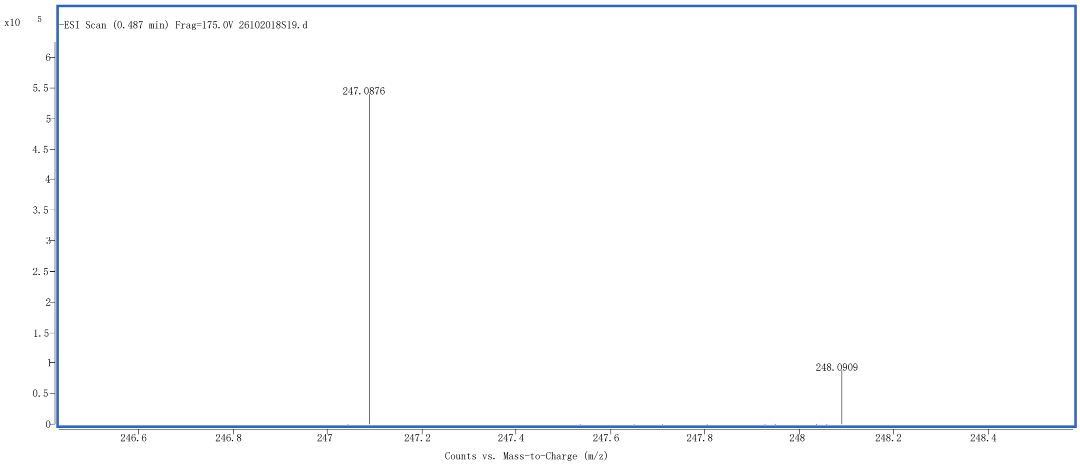
x10 5
ESI Scan (0.487 min) Frag=175.0V 26102018S19.d
247.0876
248.0909
Counts vs. Mass-to-Charge (m/z)

79

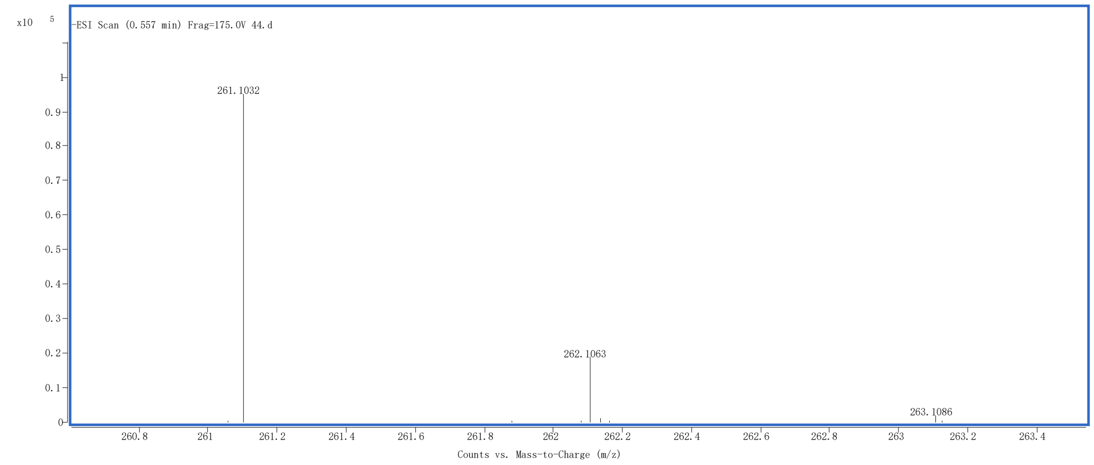
x10 5
ESI Scan (0.557 min) Frag=175.0V 44.d
261.1032
262.1063
263.1086
Counts vs. Mass-to-Charge (m/z)

80

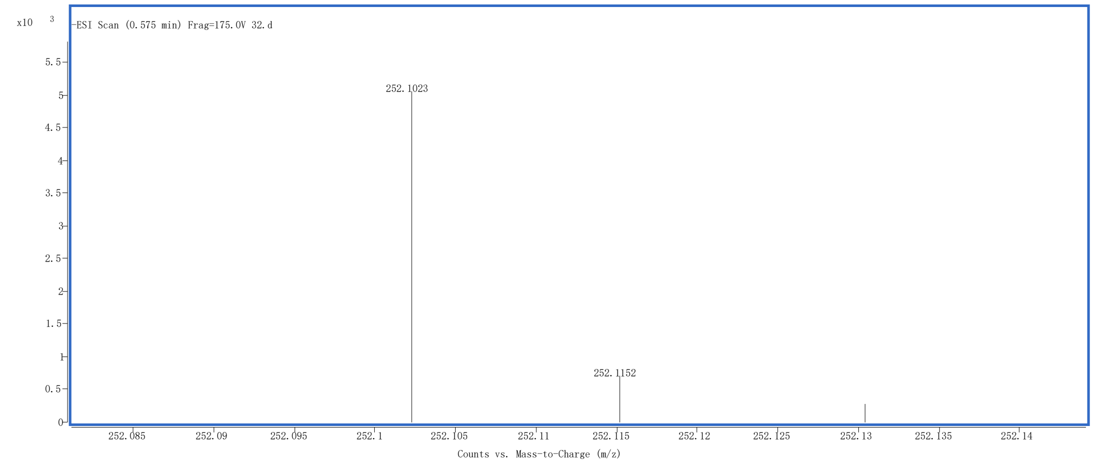
x10 3
ESI Scan (0.575 min) Frag=175.0V 32.d
252.1023
252.1152
Counts vs. Mass-to-Charge (m/z)

81

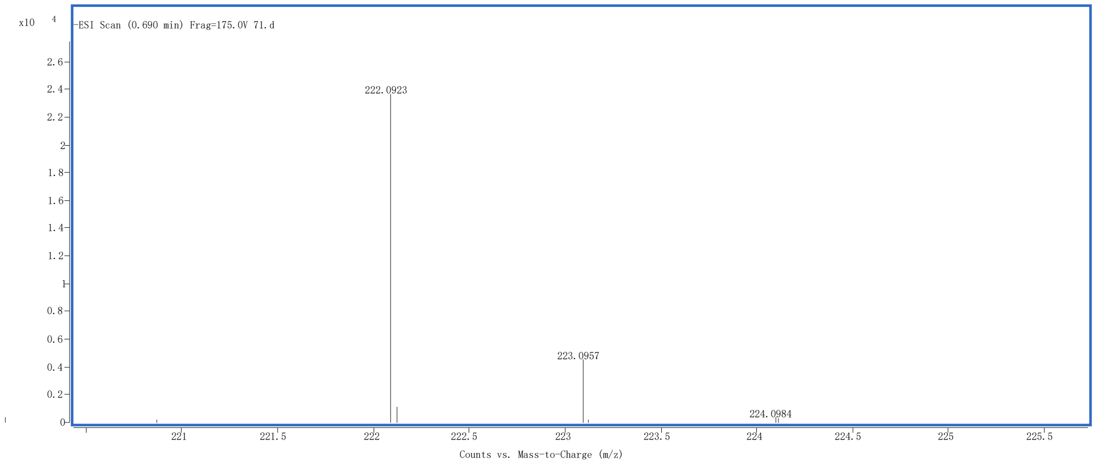
x10 4
ESI Scan (0.690 min) Frag=175.0V 71.d
222.0923
223.0957
224.0984
Counts vs. Mass-to-Charge (m/z)

82

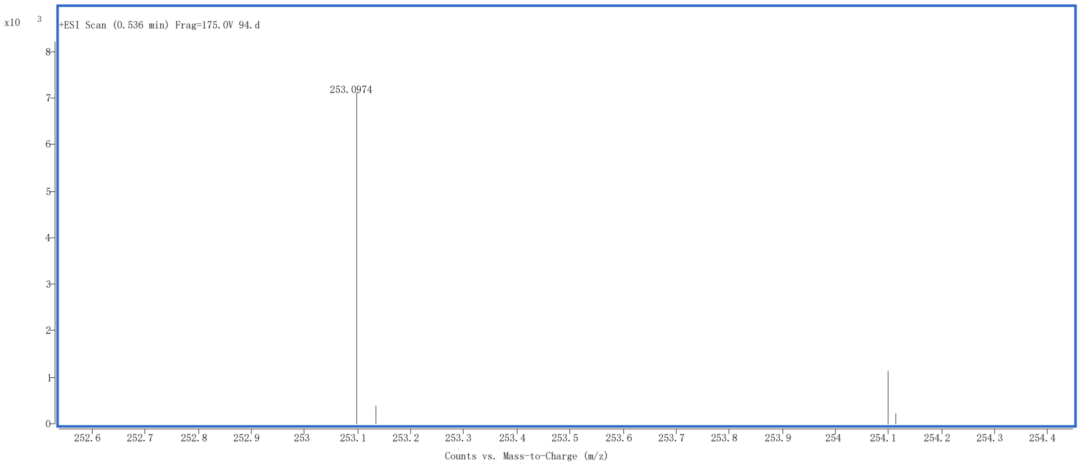
x10 3
+ESI Scan (0.536 min) Frag=175.0V 94.d
253.0974
Counts vs. Mass-to-Charge (m/z)

83

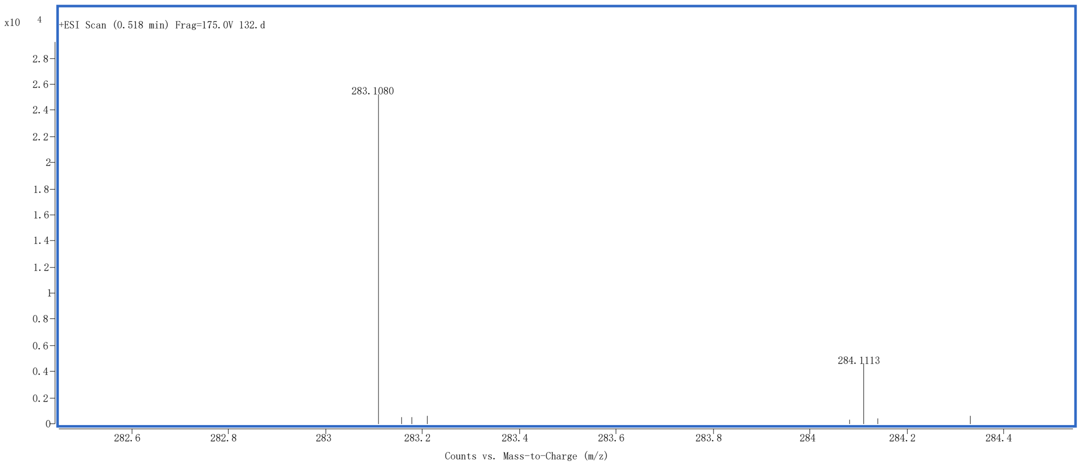
x10 4
+ESI Scan (0.518 min) Frag=175.0V 132.d
283.1080
284.1113
Counts vs. Mass-to-Charge (m/z)

84

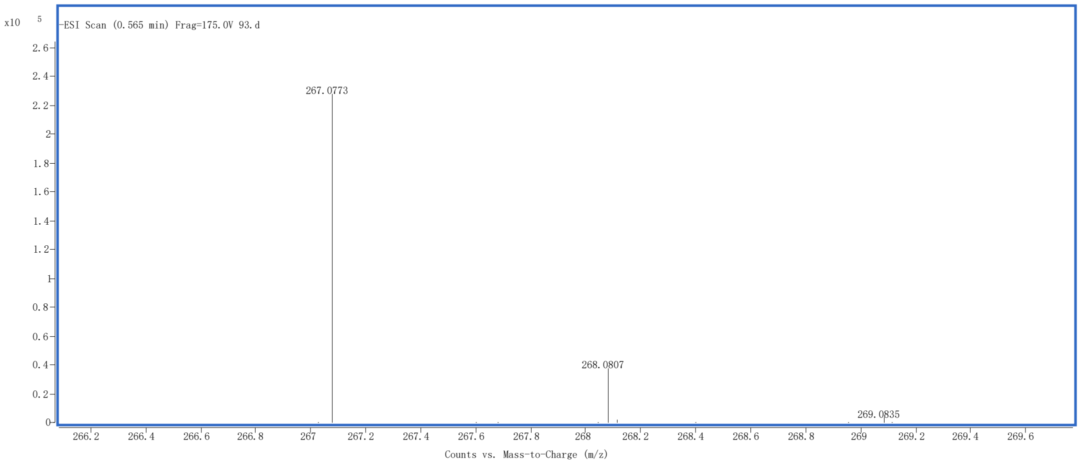
x10 5
-ESI Scan (0.565 min) Frag=175.0V 93.d
267.0773
268.0807
269.0835
Counts vs. Mass-to-Charge (m/z)

85

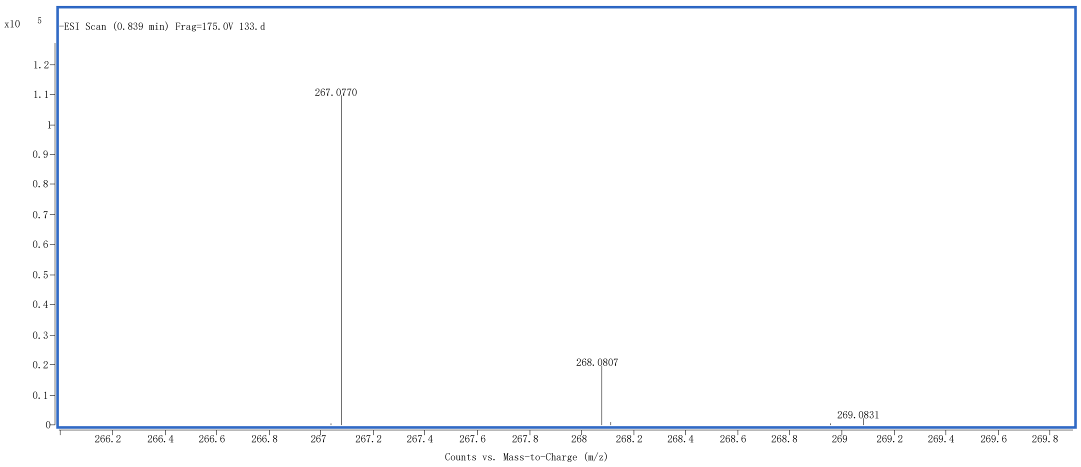
x10 5
ESI Scan (0.839 min) Frag=175.0V 133.d
267.0770
268.0807
269.0831
Counts vs. Mass-to-Charge (m/z)

86

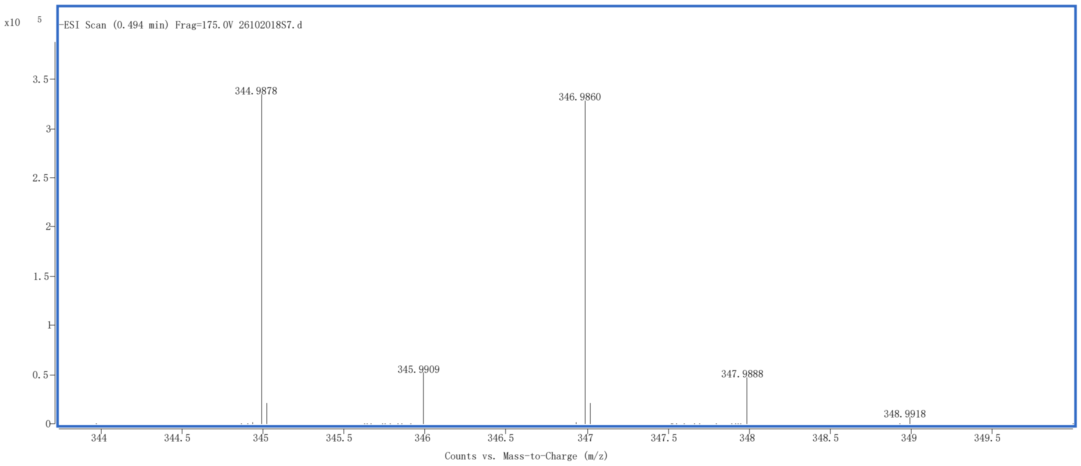
x10 5
ESI Scan (0.494 min) Frag=175.0V 26102018S7.d
344.9878
346.9860
345.9909
347.9888
348.9918
Counts vs. Mass-to-Charge (m/z)

87

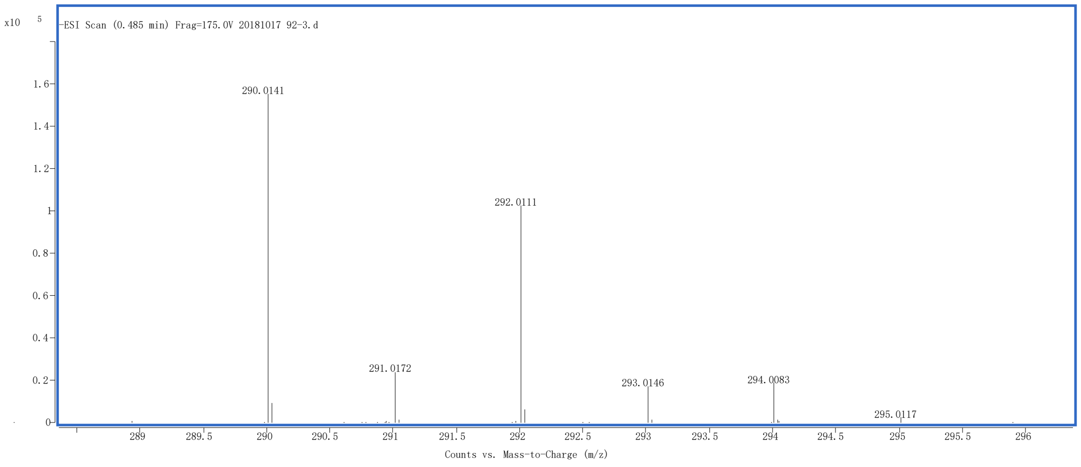
x10 5
ESI Scan (0.485 min) Frag=175.0V 20181017 92-3.d
290.0141
292.0111
291.0172
293.0146
294.0083
295.0117
Counts vs. Mass-to-Charge (m/z)

88

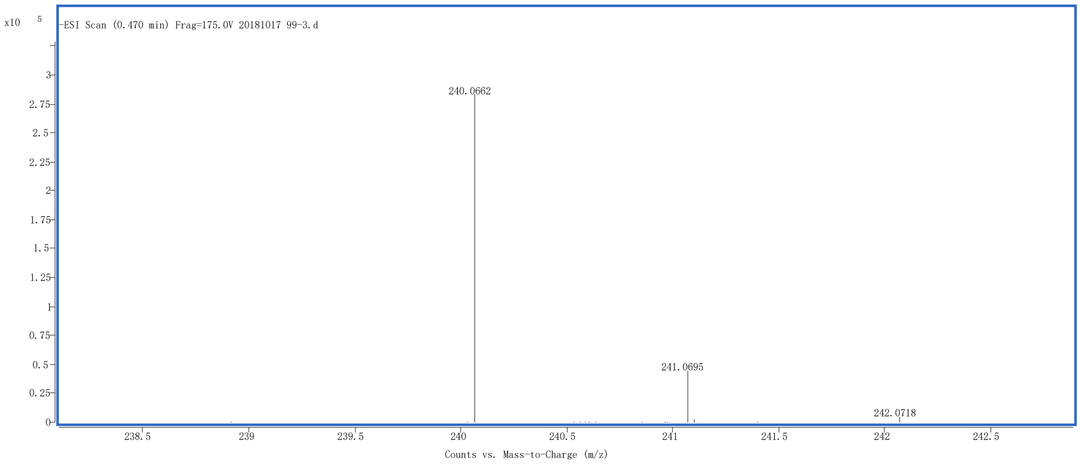
x10 5
ESI Scan (0.470 min) Frag=175.0V 20181017 99-3.d
240.0662
241.0695
242.0718
Counts vs. Mass-to-Charge (m/z)

89

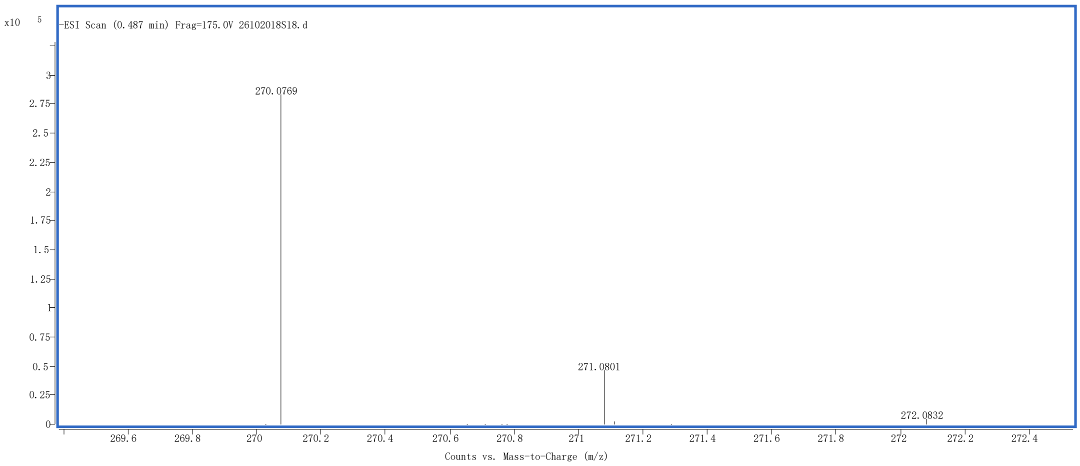
x10 5
ESI Scan (0.487 min) Frag=175.0V 26102018S18.d
270.0769
271.0801
272.0832
Counts vs. Mass-to-Charge (m/z)

90

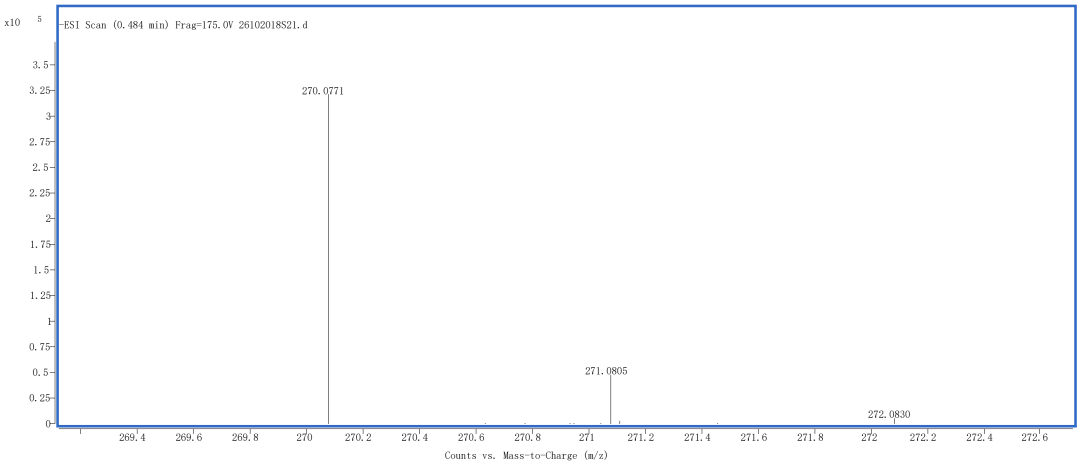
x10 5
ESI Scan (0.484 min) Frag=175.0V 26102018S21.d
270.0771
271.0805
272.0830
Counts vs. Mass-to-Charge (m/z)

91

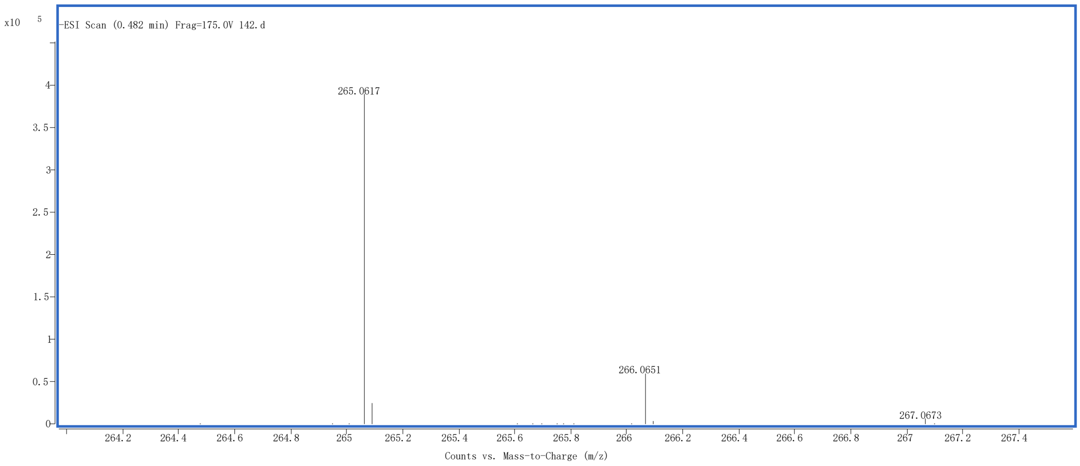
x10 5
ESI Scan (0.482 min) Frag=175.0V 142.d
265.0617
266.0651
267.0673
Counts vs. Mass-to-Charge (m/z)

92

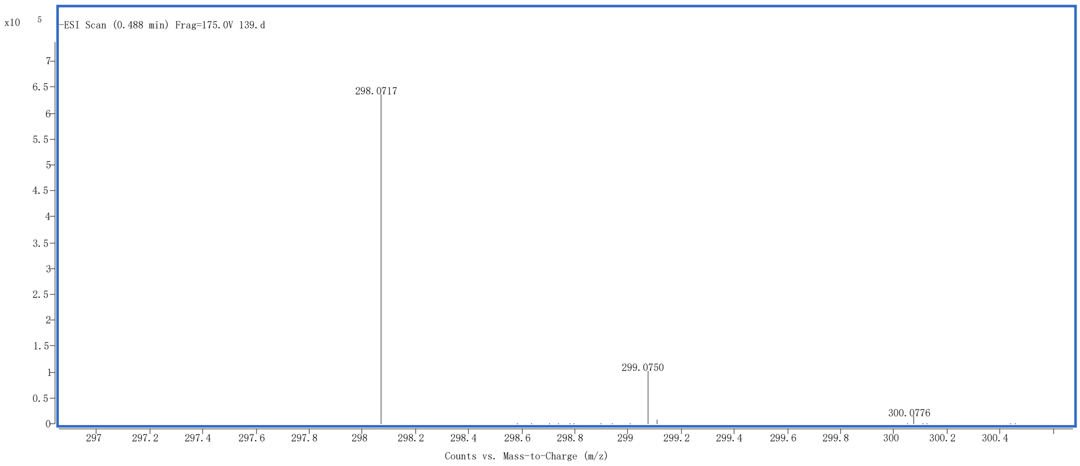
x10 5
ESI Scan (0.488 min) Frag=175.0V 139.d
298.0717
299.0750
300.0776
Counts vs. Mass-to-Charge (m/z)

93

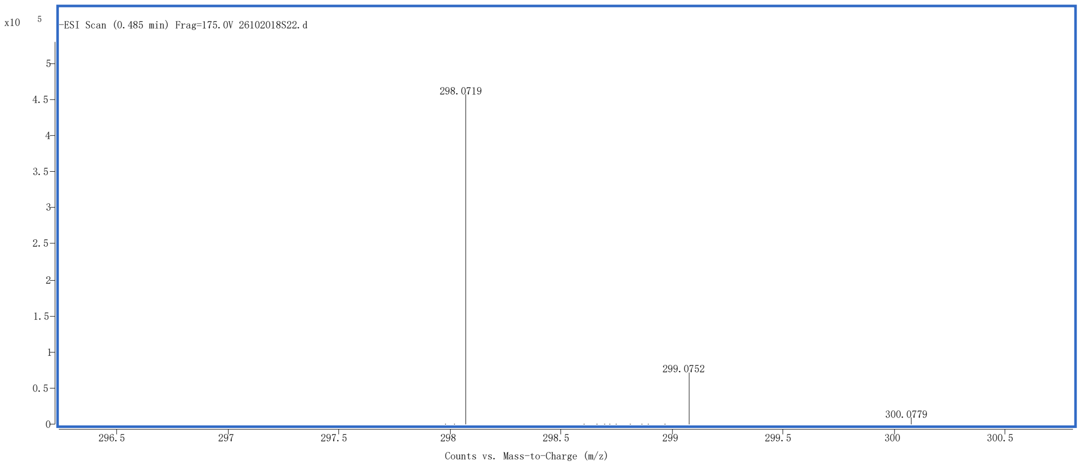
x10 5
ESI Scan (0.485 min) Frag=175.0V 26102018S22.d
298.0719
299.0752
300.0779
Counts vs. Mass-to-Charge (m/z)

94

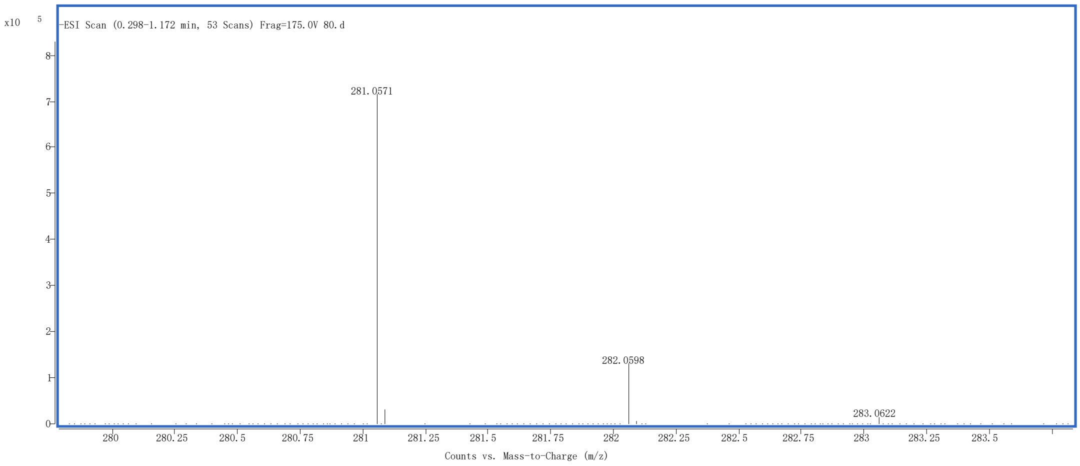
x10 5
ESI Scan (0.298-1.172 min, 53 Scans) Frag=175.0V 80.d
281.0571
282.0598
283.0622
Counts vs. Mass-to-Charge (m/z)

95

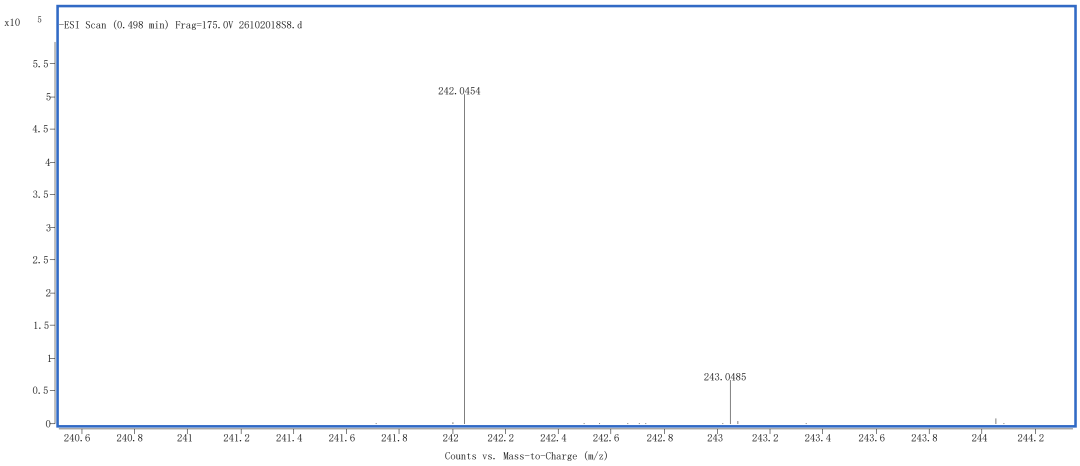
x10 5
ESI Scan (0.498 min) Frag=175.0V 26102018S8.d
242.0454
243.0485
Counts vs. Mass-to-Charge (m/z)
